# RRM1 promotes homologous recombination and radio/chemo-sensitivity via enhancing USP11 and E2F1-mediated RAD51AP1 transcription

**DOI:** 10.1038/s41420-024-02267-x

**Published:** 2024-12-18

**Authors:** Shuai Yang, Ruru Wang, Lingling Liu, Feng Xu, Xipeng Zhao, Zhicheng Yao, Jie Zhang, Xu Cheng, An Xu, Lijun Wu, Guoping Zhao

**Affiliations:** 1 Information Materials and Intelligent Sensing Laboratory of Anhui Province, Institutes of Physical Science and Information Technology, Anhui University, Hefei, Anhui China; 2 High Magnetic Field Laboratory, Key Laboratory of High Magnetic Field and Ion Beam Physical Biology, Chinese Academy of Sciences; Anhui Province Key Laboratory of Environmental Toxicology and Pollution Control Technology, Hefei Institutes of Physical Science, Chinese Academy of Sciences, Hefei, Anhui China; 3 University of Science and Technology of China, Hefei, Anhui China; 4 Anhui Province Key Laboratory of Medical Physics and Technology, Institute of Health and Medical Technology, Hefei Institutes of Physical Science, Chinese Academy of Sciences, Hefei, Anhui China; 5 Hefei Cancer Hospital, Chinese Academy of Sciences, Hefei, China

**Keywords:** Radiotherapy, Cancer therapeutic resistance

## Abstract

Ribonucleotide reductase M1 (RRM1), the catalytic subunit of ribonucleotide reductase, plays a pivotal role in converting ribonucleotides (NTP) into deoxyribonucleotides (dNTP), essential for DNA replication and repair. Elevated RRM1 expression is associated with various human cancers, correlating with poorer prognosis and reduced overall survival rates. Our previous study found that RRM1 will enter the nucleus to promote DNA damage repair. However, the underlying mechanism remains elusive. Here, we unveil a novel role of RRM1 in promoting homologous recombination (HR) by upregulating the expression of RAD51AP1, a critical HR factor, in an E2F1-dependent manner. We demonstrate that RRM1 interacts with USP11 in the cytoplasm, and the recruitment of RRM1 to LaminB1 induced by ionizing radiation (IR) facilitates the binding of USP11 to the nuclear pore complex (NPC), promoting USP11 entry into the nucleus. Upon nuclear translocation, USP11 binds to E2F1 and inhibits the ubiquitin-mediated degradation of E2F1, thereby enhancing the transcriptional expression of RAD51AP1. Moreover, a specific RRM1 mutant lacking amino acids 731–793, crucial for its interaction with USP11 and recruitment to LaminB1, exhibits a dominant-negative effect on RAD51AP1 expression and HR. Truncations of RRM1 fail to inhibit the ubiquitin-mediated degradation of E2F1 and cannot promote the E2F1-mediated transactivation of RAD51AP1. Lastly, the full length of RRM1, not truncations, enhances tumor cells’ sensitivity to IR, underscoring its importance in radiotherapy resistance. Collectively, our results suggest a novel function of RRM1 in promoting HR-mediated DSB repair through positive regulation of RAD51AP1 transcription by direct interaction with USP11 and promoting subsequent USP11-mediated deubiquitination of E2F1. Our findings elucidate a previously unknown mechanism whereby RRM1 promotes HR-mediated DNA repair, presenting a potential therapeutic target for cancer treatment.

## Introduction

Understanding how tumor cells respond to radiation is a critical aspect of precision radiotherapy in cancer treatment. Despite notable progress in this field, further research is essential to comprehensively grasp the mechanisms underlying radiation resistance and sensitivity in tumor cells.

Changes in the expression or activity of DNA damage response proteins can profoundly affect the sensitivity of tumor cells to IR [1–3]. Among different forms of DNA damage, double-strand breaks (DSBs) represent the most critical threat to genomic stability [4, 5]. DSBs can originate from replication stress as well as exposure to IR and specific chemotherapeutic agents [6]. Inadequate repair of DSBs may result in genomic instability, influencing cellular outcomes such as cell death or senescence [7–9]. In human cells, two principal pathways are involved in repairing DSBs: homologous recombination (HR) and non-homologous end joining (NHEJ) [10]. HR represents a relatively accurate and efficient repair mechanism, reliant on the availability of sister chromatid DNA, primarily occurring during the S and G2 phases of the cell cycle. Conversely, while NHEJ remains effective, it is prone to inaccuracies and may induce DNA rearrangements [11].

Ribonucleotide Reductase (RR) catalyzes the conversion of ribonucleotides to deoxyribonucleotides [12], providing the necessary building blocks (dNTPs) for DNA synthesis and repair. RR enzymes typically form heterodimeric tetramers, comprising RRM1 as the catalytic subunit and RRM2 as the regulatory subunit [12]. While RRM1 is constitutively expressed throughout the cell cycle, the expression of RRM2 is cell cycle-dependent [13]. This suggests that RRM1 may have additional cellular functions beyond its role in RR activity. RRM1 expression is elevated in various cancer tissues compared to normal tissues [14]. Consequently, RRM1 has emerged as a prognostic marker in several cancer types, including non-small cell lung cancer, pancreatic cancer, breast cancer, and biliary tract cancer [15]. Notably, patients with lower RRM1 expression tend to exhibit more favorable prognoses and longer overall survival rates than those with higher RRM1 expression levels [16].

Current research indicates that RRM1 plays a significant role in DNA damage repair. For instance, studies have shown that knocking down RRM1 leads to the upregulation of DNA damage response genes, resulting in the inhibition of tumor cell growth [17]. Additionally, RRM1 has been found to induce G2 cell cycle arrest, thereby enhancing the efficiency of DNA damage repair [18]. Individuals with high RRM1 expression levels may exhibit better protection against DNA damage induced by carcinogens [18]. Our study also has revealed that knocking down RRM1 increases DNA damage caused by IR, while IR itself promotes the expression of RRM1 and induces its migration from the cytoplasm to the nucleus [19]. Furthermore, downregulating RRM1 can modulate the ubiquitination level of p53 by affecting the interaction between p53 and USP11, thereby enhancing the radiation sensitivity of tumor cells by impeding DSB repair [19]. Despite these findings, the precise mechanisms underlying RRM1’s involvement in DNA damage repair remain unclear. Further research is warranted to elucidate the specific repair pathway to which RRM1 belongs and how it executes its repair functions.

In our study, we made the novel discovery that increasing RRM1 expression can stimulate the transcription and expression of RAD51AP1, leading to enhanced HR efficiency and increased radiation resistance in tumor cells. Moreover, our further investigations unveiled a mechanism by which IR triggers the translocation of USP11 with RRM1 into the nucleus. This allows USP11 to bind to E2F1 in the nucleus and inhibit E2F1 ubiquitination. Consequently, the elevated levels of E2F1 in the nucleus promote the transcription of RAD51AP1, thus enhancing HR-mediated DNA damage repair and ultimately improving tumor cell survival post-IR exposure. These findings shed light on the specific role of RRM1 in DNA damage repair pathways and suggest its potential as a therapeutic target for cancer treatment.

## Results

### RRM1 promotes radiation resistance in tumor cells by enhancing homologous recombination repair

**Fig. 1:**
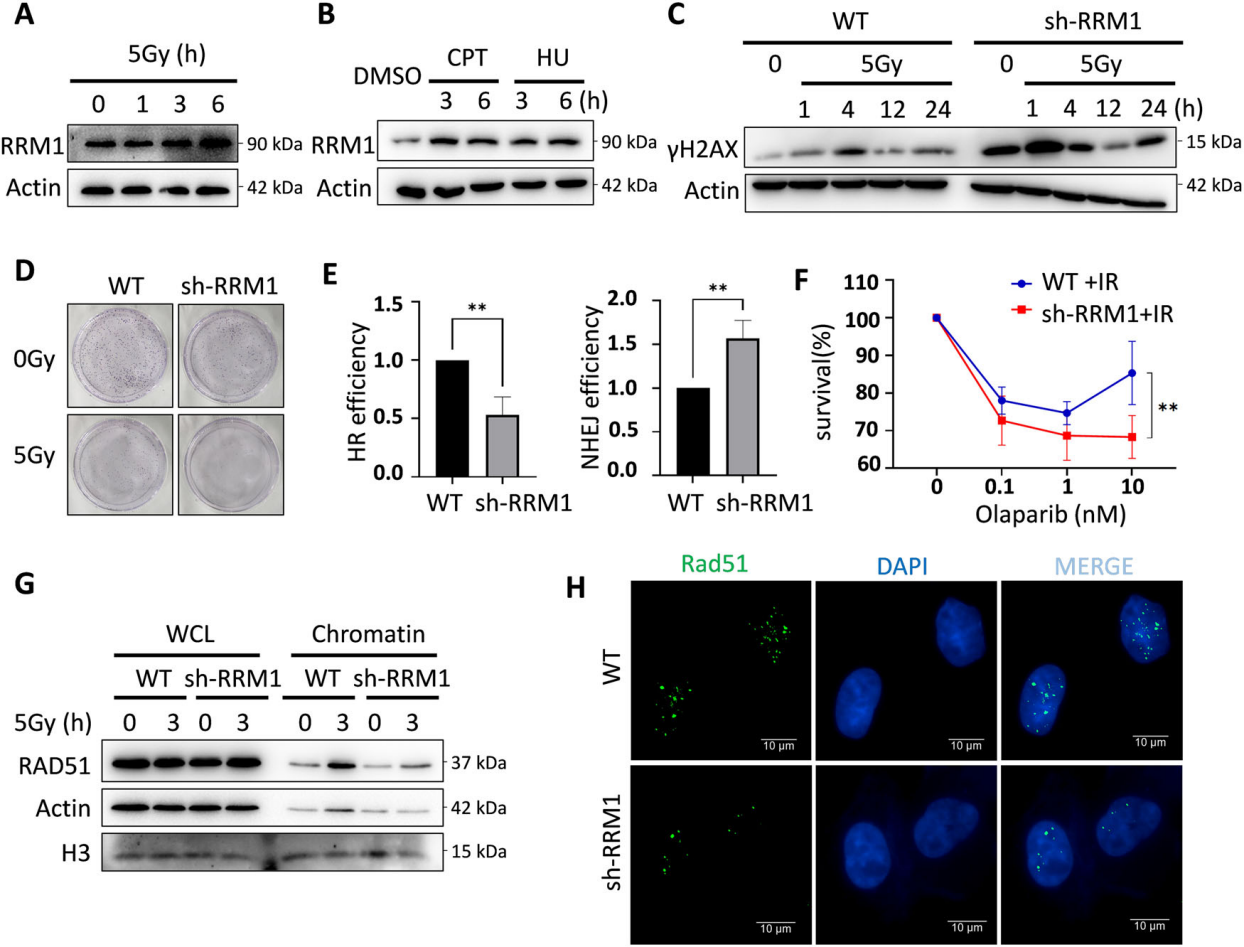
RRM1 enhances the radiation resistance of tumor cells by promoting HR repair. **A, B** Western blot analysis of RRM1 expression in HeLa cells treated with ionizing radiation and DNA damage drugs. **C** Detection of DNA damage markers γH2AX before and after ionizing radiation in HeLa wild-type and RRM1 knockdown cell lines. **D** Colony-forming assay to evaluate the colony-forming ability of HeLa wild-type and RRM1 knockdown cells before and after ionizing radiation. **E** Flow cytometry analysis to determine the proportion of GFP-positive cells indicative of HR repair (DR-GFP) or NHEJ repair (EJ5-GFP) in HeLa wild-type and RRM1 knockdown cells. **F** Sensitivity assay showing the response of HeLa wild-type cells and RRM1 knockdown cells to the PARP inhibitor olaparib after irradiation. **G** Chromatin separation experiment assessing the chromatin recruitment of RAD51 in HeLa wild-type and RRM1 knockdown cells. **H** Immunofluorescence staining to visualize the formation of RAD51 foci in HeLa wild-type and RRM1 knockdown cells after DNA damage.

We initially determined that DSB induces the expression of RRM1(Fig. 1A, B), and knockdown of RRM1 upregulated DNA damage response (Fig. 1C), further triggering significant growth inhibition following IR in tumor cells (Fig. 1D). Next, to elucidate the role of RRM1 in HR, we utilized DR-GFP and EJ5-GFP plasmid reporting systems. Here, DSBs were induced by expressing the I-SceI endonuclease, impacting the expression of the GFP gene. HR or NHEJ pathways can repair the broken I-SceI site, thereby restoring GFP gene expression. The activity of HR and NHEJ was assessed by quantifying the number of GFP+ cells post-I-SceI expression using flow cytometry. Our findings revealed that HR but not NHEJ was limited in RRM1 knocking down cells (Fig. 1E and S1A). These results provide direct evidence that RRM1 promotes DSB-induced HR. Given that HR deficiency and PARP inhibitors exert a synergistic lethal effect on tumor cells [20, 21], we sought to assess the response of RRM1 knockdown cells to PARPi. Remarkably, RRM1 knockdown cells displayed increased sensitivity to Olaparib following IR (Fig. 1F), thereby corroborating the role of RRM1 in promoting HR. Furthermore, RRM1 knockdown inhibited the recruitment of RAD51 to chromatin and the formation of RAD51 foci at the DSB site post-IR (Fig. 1G, H). Consistent with the results of the EJ5GFP reporting system, RRM1 knockdown enhanced the chromatin recruitment of the NHEJ protein ku70 and promoted the formation of 53BP1 foci (Figure S1B, C). In summary, the results presented in Fig. 1 underscore the role of RRM1 in enhancing the radiation resistance of tumor cells by promoting HR.

### RRM1 enhances HR by promoting RAD51AP1 transcription

**Fig. 2:**
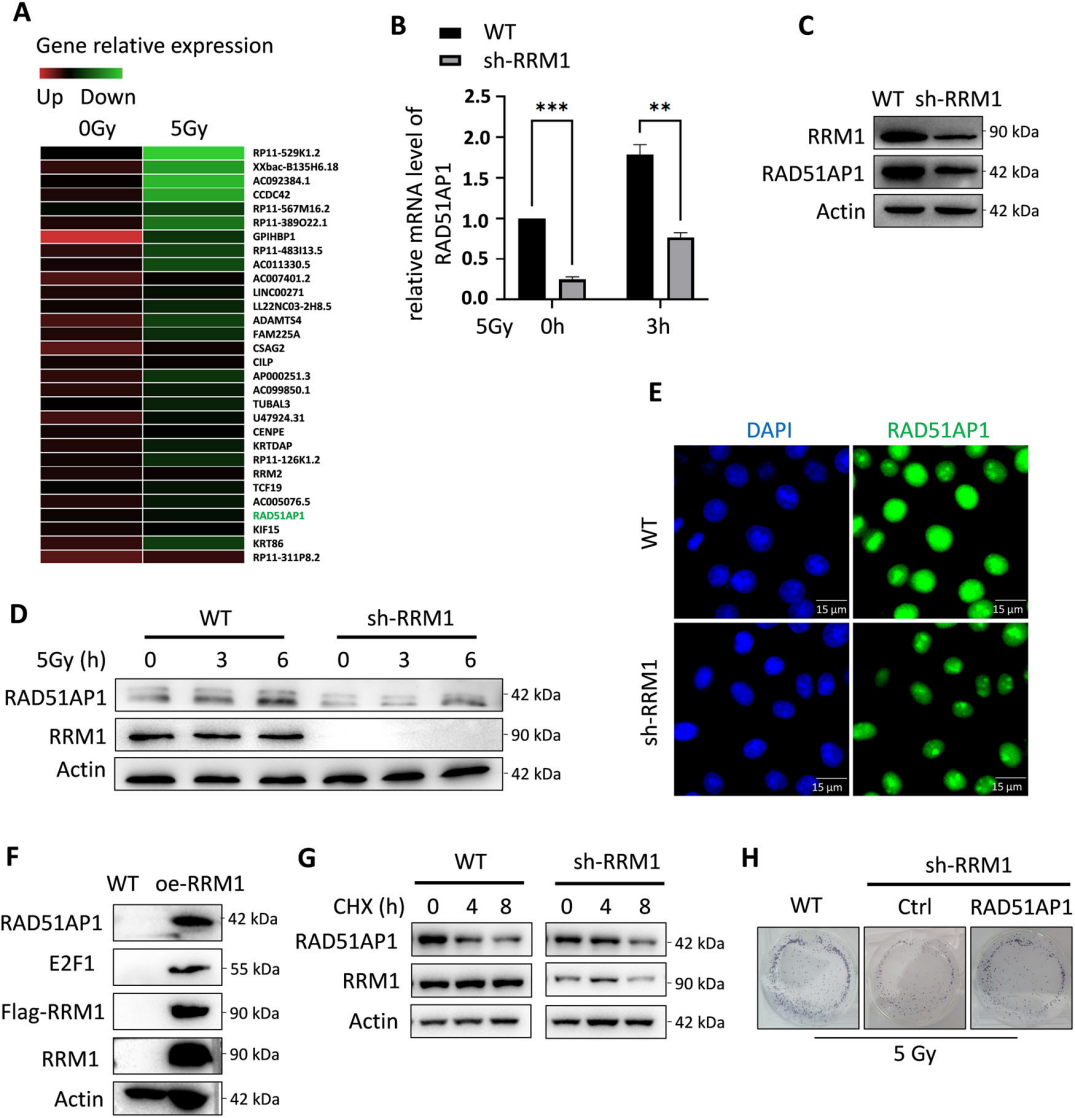
RRM1 promotes the transcription of RAD51AP1. **A** Heatmap comparison of differentially expressed genes in HCT116 and A549 cells between low and high RRM1 expression groups. **B** Measurement of RAD51AP1 mRNA expression before and after ionizing radiation (IR) in HeLa wild-type and RRM1 knockdown cells. **C** Evaluation of the effect of RRM1 knockdown on RAD51AP1 protein levels. **D** Analysis of RAD51AP1 protein levels over time in HeLa wild-type and RRM1 knockdown cells after IR. **E** Immunofluorescence staining of RAD51AP1 to assess the impact of RRM1 knockdown on RAD51AP1 protein expression. **F** Transfection of HEK293T cells with an RRM1-carrying plasmid followed by Western blot analysis to examine changes in RAD51AP1 protein levels. **G** Detect the RAD51AP1 protein content at regular intervals of WT and RRM1 knockdown HeLa cells treated with CHX. **H** Transfer the plasmid carrying RAD51AP1 into HeLa cells with knockdown of RRM1 and assay their colony-forming ability. Compare this with the colony-forming ability of HeLa wild-type and RRM1 knockdown cells following IR treatment.

We conducted transcriptome sequencing analysis to investigate whether the observed decrease in HR was attributable to altered transcription levels of HR proteins upon RRM1 knockdown. Our data revealed that knocking down RRM1 led to a downregulation in the transcription level of RAD51AP1 (Fig. 2A). Subsequently, we confirmed that RRM1 knockdown attenuated RAD51AP1 mRNA levels both pre- and post-IR (Fig. 2B). Consistently, the protein expression of RAD51AP1 was diminished in RRM1 knockdown cells (Fig. 2C). Furthermore, RRM1 knockdown impeded the accumulation of RAD51AP1 protein levels following IR (Fig. 2D). The regulation of RAD51AP1 protein levels by RRM1 was further corroborated through immunofluorescence staining of RAD51AP1 (Fig. 2E). These findings suggest that RRM1 may modulate RAD51AP1 protein levels by regulating its transcription. To further validate the role of RRM1 in regulating radiosensitivity via RAD51AP1, we introduced a plasmid carrying RAD51AP1 into RRM1 knockdown cells. We found that restoring RAD51AP1 expression was able to reverse the increased sensitivity of RRM1 knockdown cells to IR (Fig. 2H). Notably, overexpression of RRM1 led to increased RAD51AP1 expression levels (Fig. 2F) and knocking down RRM1 did not accelerate the degradation of RAD51AP1 (Fig. 2G).

Taken together, these results indicate that RRM1 promotes RAD51AP1-mediated HR by regulating RAD51AP1 transcription.

### RRM1 facilitates RAD51AP1 mRNA expression via E2F1

**Fig. 3:**
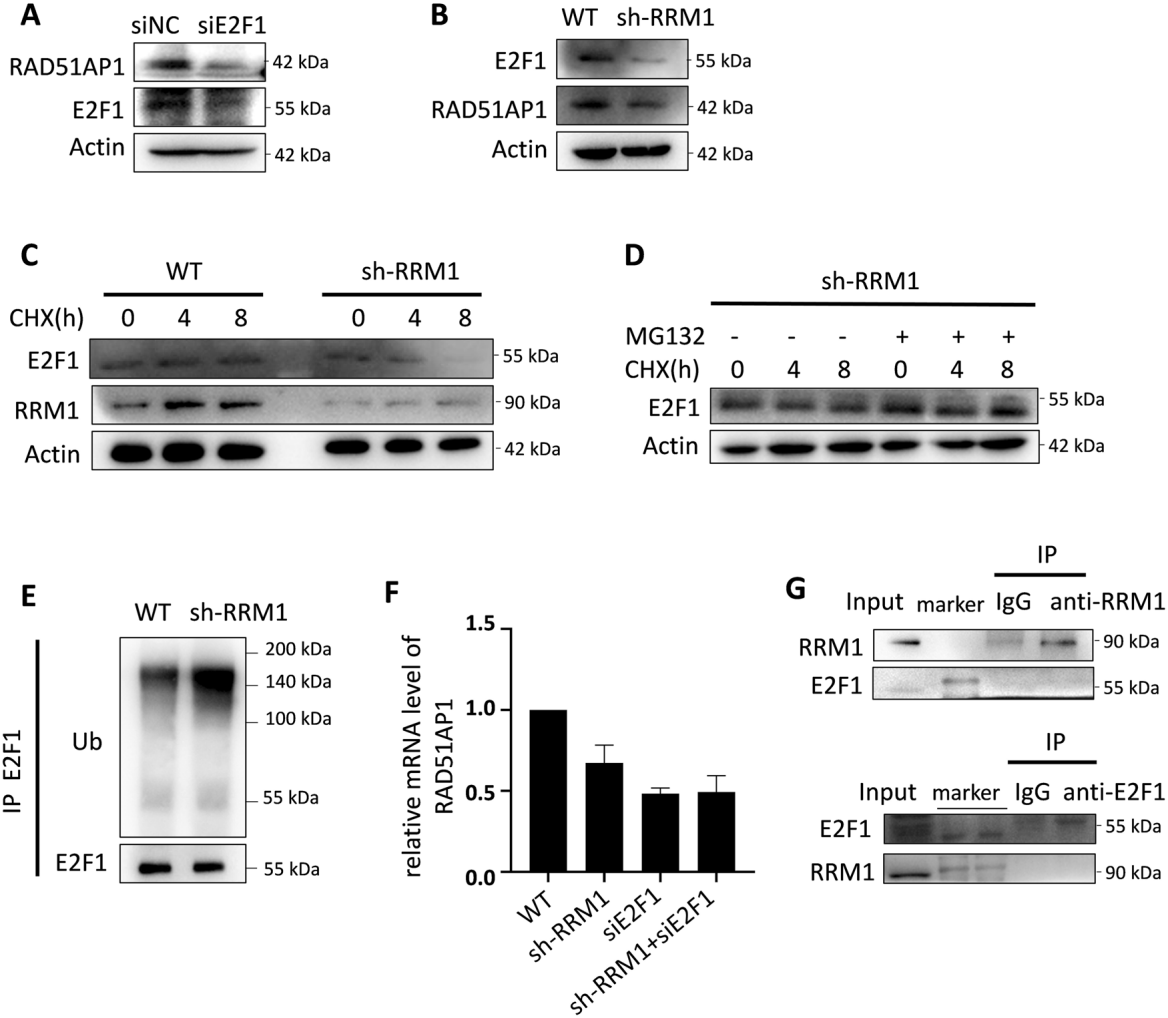
RRM1 promotes mRNA expression of RAD51AP1 dependent on E2F1. **A** Transfer of control plasmids NC and E2F1 siRNA separately, followed by detection of RAD51AP1 protein expression in HEK293T cells. **B** Detection of E2F1 protein expression in HeLa wild-type and RRM1 knockdown cell lines. **C** Detection of E2F1 protein levels in wild-type and RRM1 knockdown cells at intervals after adding the protein synthesis inhibitor CHX. **D** Evaluation of the effect of proteasome inhibitor MG132 on E2F1 protein levels in HeLa RRM1 knockdown cell lines treated with CHX. **E** Detection of ubiquitination levels of E2F1 in HeLa wild-type and RRM1 knockdown cell lines, respectively. **F** Transfer siE2F1 into RRM1 knockdown HeLa cells and detect the transcriptional changes of RAD51AP1. **G** Immunoprecipitation of E2F1 and RRM1 in HeLa wild-type cells using anti-RRM1 antibody and E2F1 antibody, respectively, followed by detection of the protein levels of E2F1 and RRM1 by Western blot.

Given that E2F1 serves as a known transcription factor for RAD51AP1 promoter activation [22, 23], we investigated whether E2F1 is involved in RRM1-mediated regulation of RAD51AP1 expression. Initially, we constructed plasmids expressing siRNAs targeting E2F1 and observed a subsequent inhibition of RAD51AP1 protein expression upon interference with the E2F1 gene (Fig. 3A). Consequently, we hypothesized that RRM1 may regulate the expression level of E2F1. Indeed, RRM1 knockdown led to reduced levels of E2F1 protein compared to wild-type cells, while overexpression of RRM1 resulted in elevated levels of E2F1 protein (Fig. 3B and Fig. 2F). Further experiments revealed that knocking down RRM1 accelerated the degradation rate of E2F1 (Fig. 3C). The addition of proteasome inhibitor MG132 prevented E2F1 degradation (Fig. 3D), indicating that RRM1 may influence the ubiquitin-mediated degradation of E2F1 [24]. As speculated, knocking down RRM1 enhanced the ubiquitination level of E2F1 (Fig. 3E). To further verify that E2F1 mediates the transcriptional regulation of RRM1 on RAD51AP1, we examined the effect of knocking down RRM1 followed by interference with E2F1 expression on RAD51AP1 transcription. The result show that interfering with E2F1 expression did not further reduce RAD51AP1 transcription (Fig. 3F), suggesting that RRM1 and E2F1 regulate RAD51AP1 through the same pathway. Additionally, protein immunoprecipitation experiments revealed no direct interaction between RRM1 and E2F1 (Fig. 3G). Thus, we propose that RRM1 regulates RAD51AP1 expression through E2F1-mediated transcription.

### RRM1 facilitates nuclear entry of USP11

**Fig. 4:**
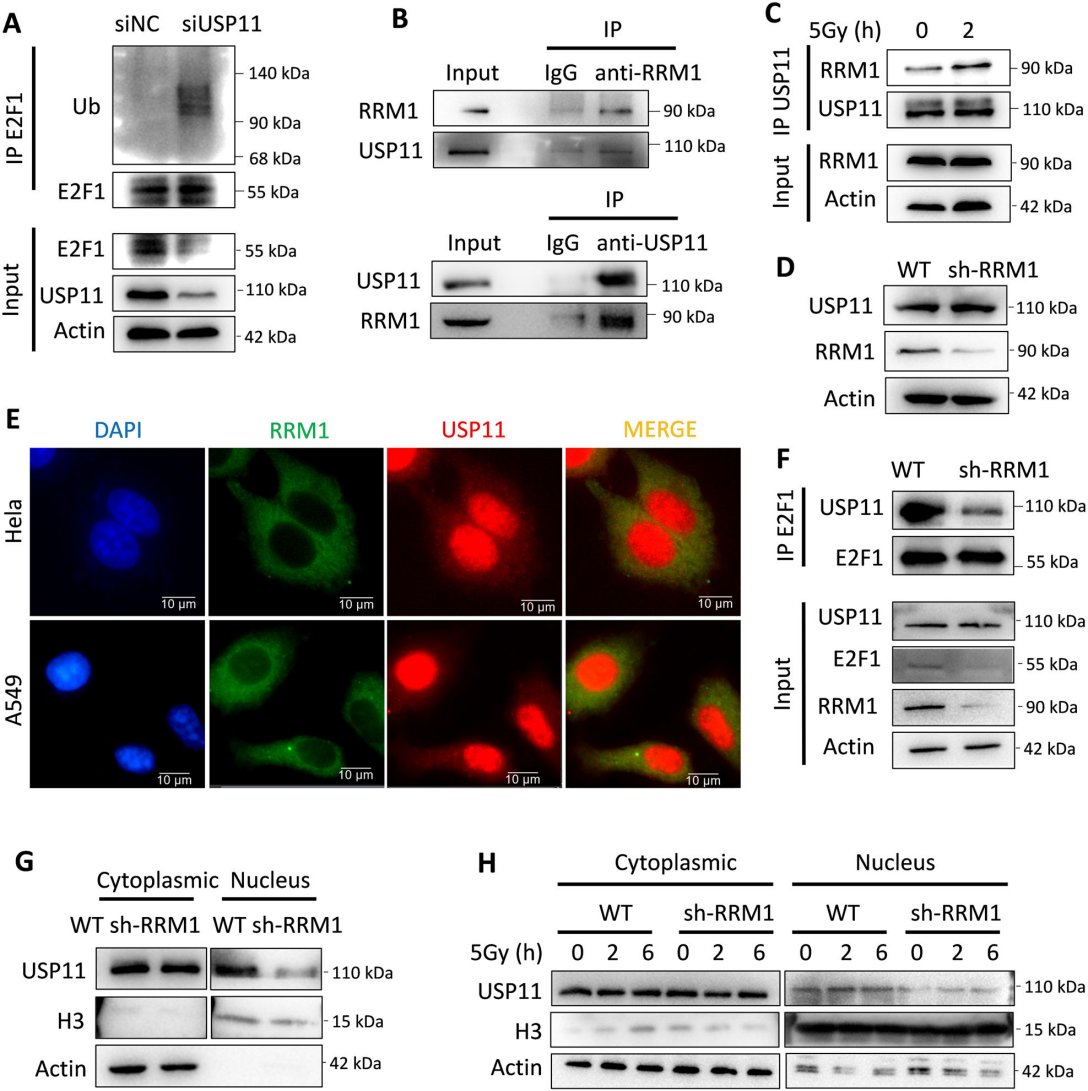
RRM1 facilitates the entry of USP11 into the nucleus. **A** After transfection with USP11 siRNA for 48 h, the levels of E2F1 ubiquitination and protein in the HEK293T cells were detected. **B** Immunoprecipitation of USP11 and RRM1 in HeLa cells using anti-RRM1 antibody and anti-USP11 antibody, respectively, followed by detection of the levels of the two proteins through Western blot, with IgG as a negative control. **C** Detection of changes in the interaction between RRM1 and USP11 in HeLa cells irradiated with 5 Gy at 0 and 2 h, respectively. **D** Detection of the expression of USP11 protein in HeLa wild-type and RRM1 knockdown cell lines separately. **E** Immunofluorescence labeling of RRM1 and USP11, respectively, to detect their co-localization in HeLa and A549 cells. **F** Detection of the amount of USP11 protein extracted by anti-E2F1 antibodies in HeLa wild-type and RRM1 knockdown cells by Western blot. **G** Evaluation of the effect of knocking down RRM1 on the protein content of USP11 in the cytoplasm and nucleus through nuclear-cytoplasmic separation experiments. **H** Detection of the cytoplasmic and nuclear protein levels of USP11 in HeLa wild-type and RRM1 knockdown cell lines at regular intervals after 5 Gy irradiation.

Studies suggest that USP11 acts as a deubiquitinase of E2F1, with downregulation of USP11 resulting in increased ubiquitination and decreased stability of E2F1 [24, 25]. We transfected specific siRNA targeting USP11 into cells, leading to increased ubiquitination and reduced protein levels of E2F1(Fig. 4A). Our previous research demonstrated that RRM1 increases the ubiquitination of p53 by promoting the binding of ubiquitin ligase MDM2 to p53 and inhibiting the binding of deubiquitinase USP11 to p53 [19], indicating a potential yet unknown relationship between RRM1 and USP11. Therefore, we speculated whether RRM1 regulates the stability of E2F1 through USP11. Here, we found that there is an interaction between RRM1 and USP11 (Fig. 4B), primarily occurring in the cytoplasm (Fig. 4E). Moreover, their interaction was enhanced after IR (Fig. 4C). Notably, knocking down RRM1 did not affect the protein level of USP11 but inhibited its binding to E2F1 (Fig. 4D, F). Since USP11 primarily exerts deubiquitination activity on E2F1 in the nucleus [26], we speculate that RRM1’s interaction with USP11 alters its subcellular localization. Nuclear-cytoplasmic separation experiments confirmed this hypothesis, showing that knocking down RRM1 altered USP11’s subcellular localization and inhibited its accumulation in the nucleus after IR (Fig. 4G, H and S2B). Furthermore, nuclear degradation experiments found that knocking down RRM1 did not affect USP11’s degradation rate in the nucleus (Figure S2A). Thus, we propose that the interaction between RRM1 and USP11 promotes USP11 migration from the cytoplasm to the nucleus.

### RRM1 facilitates USP11 binding to the Nuclear Pore Complex (NPC)

**Fig. 5:**
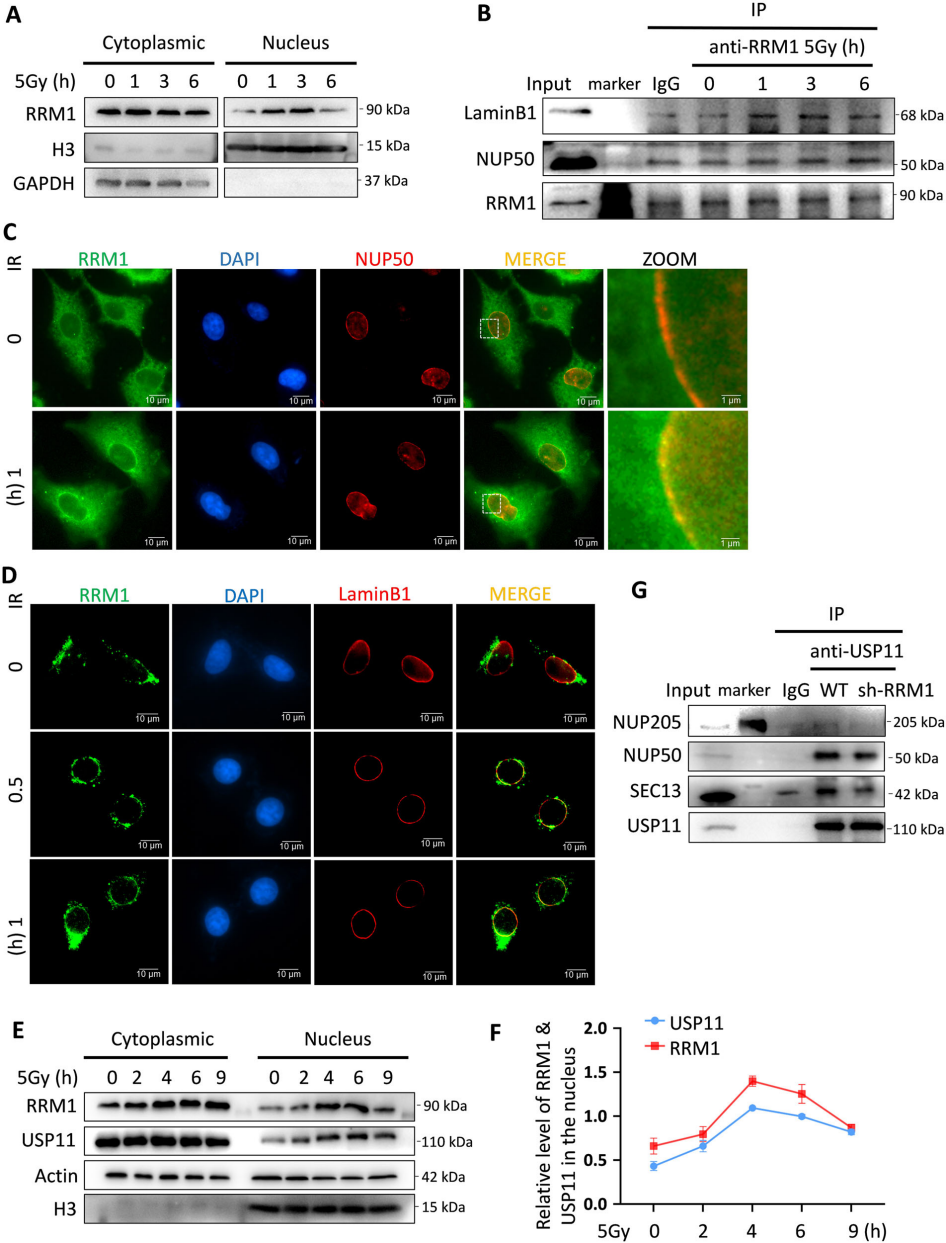
RRM1 promotes the binding of USP11 to NPC. **A** Nuclear-cytoplasmic separation analysis was performed on HeLa wild-type cells at certain intervals after 5 Gy irradiation to detect the expression of RRM1 in the cytoplasm and nucleus. **B** HeLa cells were treated with 5 Gy irradiation, and the protein levels of LaminB1 and NUP50 co-precipitated with anti-RRM1 antibody were detected by Western blot at different time points. **C** After treatment of HeLa cells with 5 Gy irradiation, the distribution of RRM1 and NUP50 at different time points after irradiation was observed under a fluorescence microscope. **D** After treatment of cells with 5 Gy irradiation, the distribution of RRM1 and LaminB1 at different time points after irradiation was observed under a fluorescence microscope. **E, F** Nuclear-cytoplasmic separation analysis was performed on HeLa wild-type cells at certain intervals after 5 Gy irradiation to detect the expression of RRM1 and USP11 in the cytoplasm and nucleus. The relative quantities of RRM1 and USP11 in the nucleus and internal reference H3 were plotted at each time point based on the grayscale values of WB bands. **G** The protein levels of several major nuclear pore complexes co-precipitated with anti-USP11 antibodies were detected in HeLa wild-type and RRM1 knockdown cells, with IgG as a negative control.

Upon irradiation, RRM1 aggregates into the nucleus (Fig. 5A). Notably, RRM1 tends to accumulate near the nuclear membrane and nuclear pore after entering the nucleus in response to irradiation (Fig. 5C, D). This phenomenon was further confirmed through immunoprecipitation experiments, showing increased binding of RRM1 to laminB1 and NUP50 after irradiation (Fig. 5B). We hypothesized that the migration of RRM1 from the nuclear pore to the nuclear fiber layer might carry USP11 and promote its binding to the NPC, facilitating USP11’s entry into the nucleus. To elucidate this mechanism, we separately analyzed the entry kinetics of RRM1 and USP11 after irradiation. Remarkably, RRM1 and USP11 entered the nucleus synchronously (Fig. 5E, F) and their interaction gradually increased during this process (Figure S3A). Since USP11 requires a nuclear localization signal (NLS) to enter the nucleus [27–29], we investigated whether the combination of RRM1 and USP11 promotes the interaction between USP11 and NPC proteins. Indeed, knocking down RRM1 prevented the binding of USP11 to certain major nuclear pore components (Fig. 5G). These results suggest that irradiation induces RRM1 transport into the nucleus, and the interaction between RRM1 and USP11 aids in facilitating the association between USP11 and NPC, thereby promoting USP11’s nuclear translocation.

### The C-terminus of RRM1 facilitates USP11 binding and nuclear entry

**Fig. 6:**
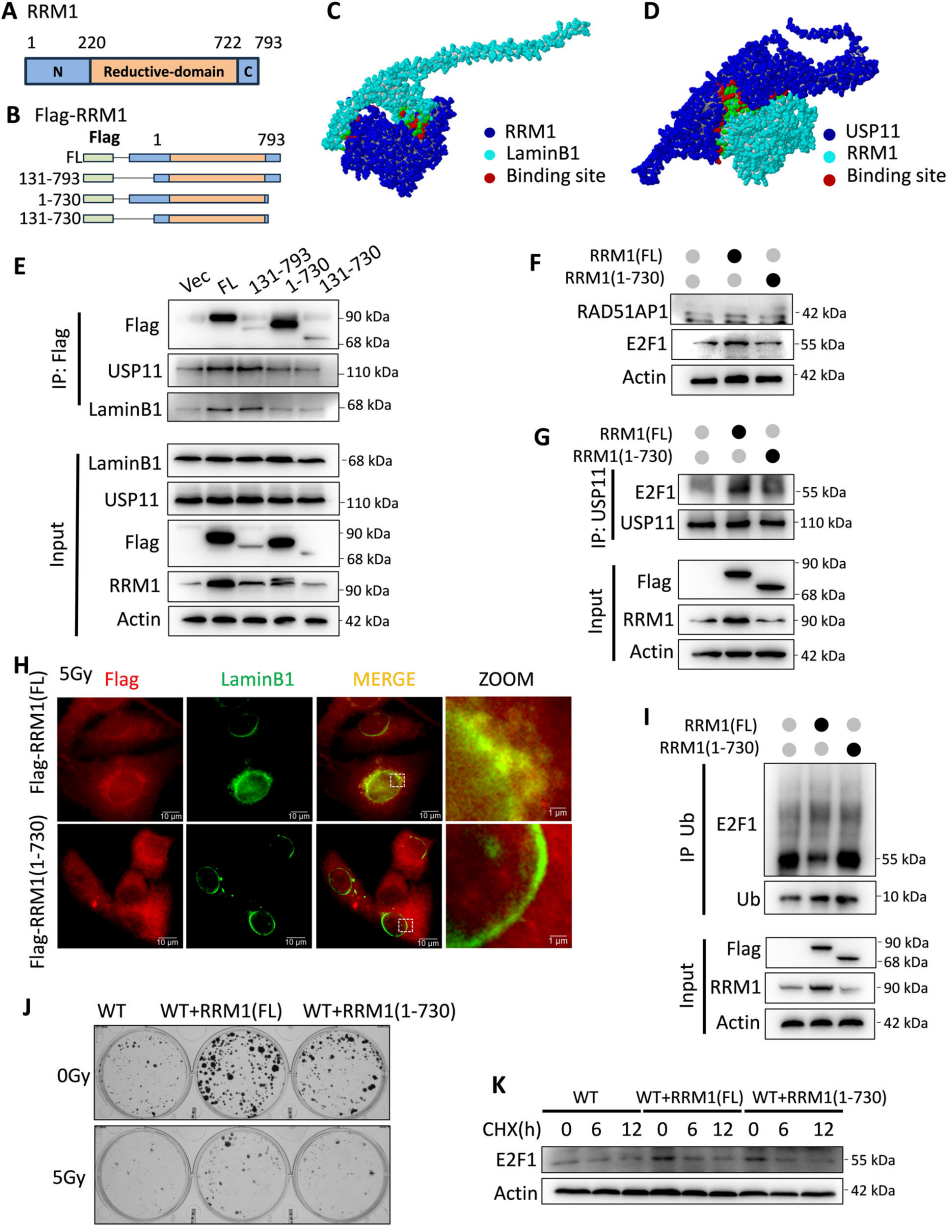
The C-terminus of RRM1 is crucial for RRM1 to bind to USP11 and facilitate USP11’s entry into the nucleus. **A** Structural sequence of RRM1 based on literature. **B** Schematic diagram of the RRM1 truncated body model with missing sequences. **C-D** Potential interaction sites between RRM1, USP11, and LaminB1 predicted using online protein structure prediction software. **E** Transfection of HEK293T cells with various RRM1 constructs, followed by treatment with 5 Gy irradiation. Immunoprecipitation with anti-flag antibody and analysis of the precipitate by immunoblotting with RRM1, USP11, LaminB1, Flag, and Actin. **F** Comparison of expression levels of E2F1 and RAD51AP1 proteins in HeLa cells transfected with different RRM1 constructs compared to wild-type cells. **G** Detection of the protein level of E2F1 co-precipitated with anti-USP11 antibody immunoblotting after transfection with full-length and truncated RRM1 constructs. **H** Analysis of the distribution of RRM1 and its truncated bodies within HEK293T cells after 5 Gy treatment using fluorescence immunoassay. **I** Detection of E2F1 co-precipitated with Ub after transfection with full-length and truncated RRM1 constructs. **J** Observation of colony-forming ability of HEK293T cells under irradiation and non-irradiation conditions after transfection with full length and truncated RRM1 constructs. **K** CHX treatment of HEK293T cells carrying full-length and truncated RRM1 constructs, followed by detection of the protein levels of E2F1 by Western blotting at different time points.

RRM1 comprises three structural domains [30–33], including a helical N-terminal domain, an α/β parallel domain, and an αβααβ domain, with the active site located between the N-terminus and C-terminus (Fig. 6A). Using an online server, we predicted and designed several RRM1 mutants (Fig. 6B–D). Deletion of the C-terminus (731–793) significantly disrupted the interaction between RRM1 and USP11/LaminB1(Fig. 6E), as confirmed by immunofluorescence showing impaired recruitment to LaminB1 after irradiation (Fig. 6H). This suggests that the C-terminal region outside the catalytic domain of RRM1 is crucial for promoting HR and enhancing cell radiation resistance. To validate this, we transfected plasmids containing full-length RRM1 or a truncated version lacking the C-terminus (1–730) into cells. Cells transfected with full-length RRM1 exhibited higher expression levels of E2F1 and RAD51AP1 proteins compared to those transfected with the C-terminally truncated RRM1 plasmid (Fig. 6F), indicating that truncation affects protein expression. Importantly, the truncation failed to facilitated USP11 nuclear migration and subsequent deubiquitination of E2F1 (Fig. 6G, I), thereby cannot preventing E2F1 degradation (Fig. 6K).

Consistently, colony formation assays showed that full-length RRM1 conferred stronger radiation resistance compared to the C-terminally deficient RRM1 truncated plasmid (Fig. 6J). These findings underscore the critical role of the C-terminus of RRM1 in facilitating USP11 binding and nuclear entry, thereby promoting USP11-mediated deubiquitination of E2F1, leading to increased transcription of RAD51AP1 and ultimately enhancing cell radiation resistance.

## Discussion

Since the discovery of the activity of the RR reducing enzyme family by Reichard et al. in 1961 [34], significant progress has been made in understanding their structure, function, and biological significance [35–37]. However, many aspects of mammalian RR mechanisms still require further investigation, including oligomerization dynamics, subcellular localization, and the specific effects of reductase and potential non-reductase functions on tumor biology, progression, and treatment sensitivity. Of particular interest is the large subunit of RR, RRM1, which shows promise as a prognostic indicator for radiotherapy and chemotherapy in various cancers. Its expression, independent of the cell cycle [13], suggests potential non-enzymatic functions beyond reductase activity. This study delves into RRM1’s non-ribonucleotide reductase function, focusing on its role in DNA damage repair and its impact on tumor cell radiation sensitivity.

The primary function of RRM1 is to generate essential raw materials for DNA synthesis by reducing and producing ribonucleotides [5]. It is widely expressed in various types of cancer and is associated with drug resistance, cancer cell proliferation, and metastasis [6]. Recent evidence suggests that RRM1 plays a role in the response to IR and DNA-damaging drugs [7]. Knocking down RRM1 increases DNA damage in tumor cells [4], while upregulating the expression of DNA damage-responsive genes and prolonging the DNA damage repair process [8, 9], indicating RRM1’s involvement in DNA damage repair. In this study, we have unveiled a direct association between RRM1 and HR repair. We discovered a novel function of RRM1 in DNA damage repair, wherein it regulates HR and promotes tumor cell survival by modulating the transcription of RAD51AP1. After IR exposure, RRM1 is recruited to LaminB1 through nuclear pores, facilitating the entry of its binding partner USP11 into the nucleus, thus stabilizing E2F1 and enhancing the transcriptional expression of RAD51AP1. Overall, our findings suggest that RRM1 deficiency can compromise cancer cell survival following DNA damage, and targeting RRM1 may enhance the efficacy of radiotherapy.

RRM1 also promotes the repair of DSB and enhances the radiation resistance of tumor cells by regulating the ubiquitination level of DDR protein. For instance, our previous research has demonstrated that RRM1 can impact the radiation sensitivity of tumor cells by modulating the USP11-mediated ubiquitination level of p53 [4]. Nevertheless, the mechanism by which RRM1 regulated p53 ubiquitination via USP11 has not been fully elucidated. Here, we found that RRM1 binds to USP11 via its C-terminus, increasing the likelihood of interaction between USP11 and the NPC, thereby facilitating the nuclear entry of USP11 and its subsequent deubiquitination activity on E2F1. In summary, our findings provide direct evidence for the regulation of ubiquitination levels of DNA damage repair factors by RRM1.

Furthermore, we unexpectedly observed co-localization of RRM1 with the laminB1 protein following irradiation. Subsequent immunoprecipitation experiments confirmed that IR induces the binding of RRM1 and laminB1 (Fig. 4). It is known that the outer nuclear layer of the nucleus is conducive to NHEJ, while the central area and nuclear pore favor HR for DSB repair [38–43]. This raises the question of whether RRM1 plays a role in determining the selection of DSB repair pathways. Our findings indicate that knocking down RRM1 resulted in reduced foci of the HR protein RAD51 in the central region of the nucleus and decreased foci of the NHEJ protein 53BP1 in the peripheral region (Fig. 1H and S4A, B). Given RRM1’s inherent function in inducing nucleotide triphosphate (NTP) to deoxynucleotide triphosphate (dNTP) conversion [44, 45], which can influence the selection of DNA damage repair pathways [46, 47], we hypothesize that the binding of RRM1 and laminB1 after IR may be involved in pathway selection near the nuclear periphery. Therefore, we speculate that DSBs induce the migration of RRM1 towards the nuclear periphery, where it functions as a reductase, creating a local environment characterized by low NTP and high dNTP levels. This environment may regulate the selection of the DDR pathway. However, further experimental verification is required to confirm these inferences.

**Fig. 7:**
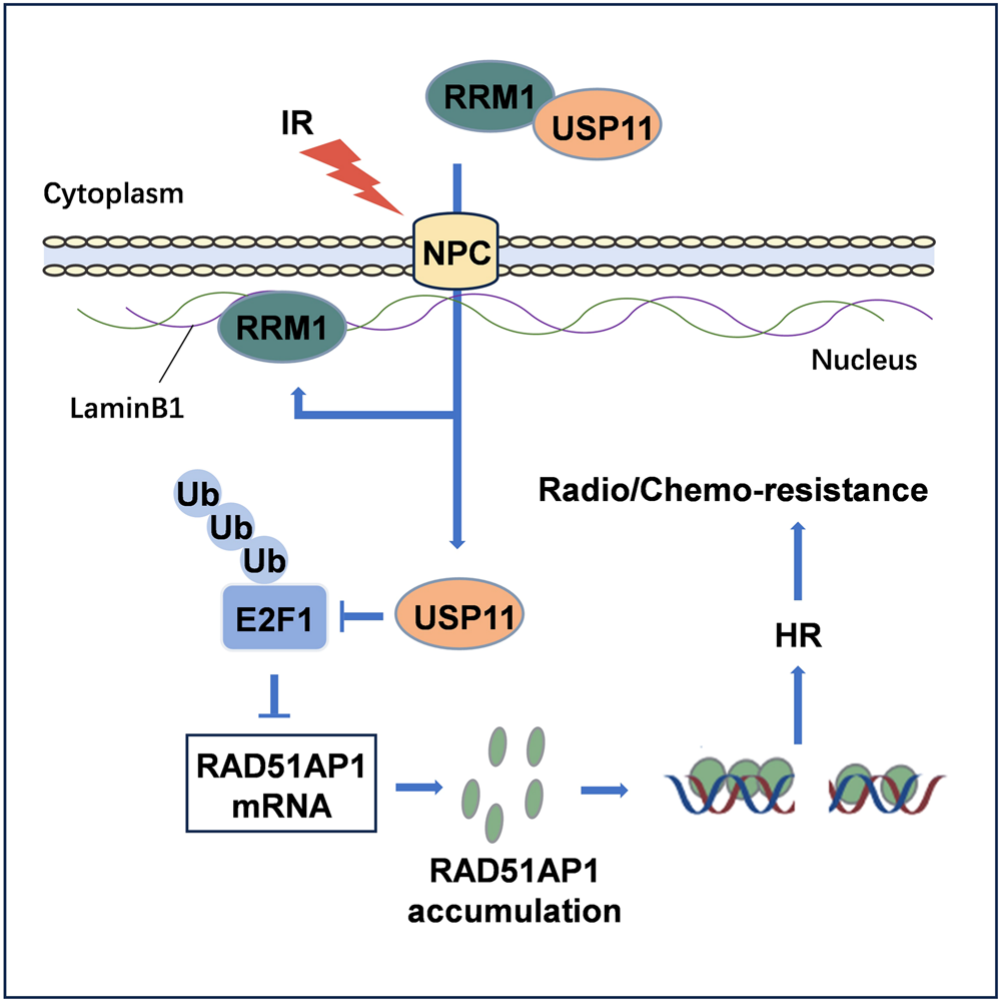
Schematic diagram showing the role of RRM1 in regulating DSB repair. RRM1 interacts with USP11 in the cytoplasm. IR induces the entry of RRM1 into the nucleus while promoting the entry of USP11. USP11 in the nucleus can exert a deubiquitination function on E2F1 and inhibit the ubiquitination degradation of E2F1. High levels of E2F1 promote the transcription of RAD51AP1, thereby enhancing HR and the radiation sensitivity of tumor cells.

Overall, our study has unveiled a novel role of RRM1 in regulating DNA damage repair. Furthermore, by demonstrating the direct interaction between RRM1 and USP11, our research has linked RRM1 to RAD51AP1 and elucidated how this interaction promotes E2F1-dependent RAD51AP1 expression (Fig. 7). Our findings expand the understanding of RRM1’s biological function, providing new insights into its role in cancer biology and offering a potential target for cancer treatment.

## Materials and methods

### Cell culture and radiation

A549, HeLa, and HEK293T cell lines were obtained from Beyotime (Shanghai, China) and cultured in DMEM (VivaCell BIOSCIENCES) supplemented with 10% fetal bovine serum (Biological Industries, Israel) and 1×penicillin and streptomycin (100 U/mL, Gibco, NY, USA) in a 5% CO_2_ incubator at 37 °C. Irradiation was performed using a Biobeam GM gamma irradiator (Leipzig, Germany) with a dose rate of 3.27 Gy/min.

### Antibodies

The following antibodies were purchased from Proteintech (IL, USA): anti-RRM1 (60073-2-Ig), anti-RAD51AP1 (11255-1-AP), anti-USP11 (10244-1-AP), anti-E2F1 (66515-1-Ig), anti-RAD51 (14961-1-AP), anti-Actin (66009-1-Ig), anti-H3 (17168-1-AP), and anti-Ub (10201-2-AP). Antibodies from Abcam included anti-γH2AX (ab81299), anti-LaminB1 (ab16048), anti-RAD51 (ab133534), and anti-53BP1 (ab175933). The anti-FLAG antibody (AF0036) was obtained from Beyotime (Shanghai, China).

### Stable cell line construction

Briefly, transduced HeLa and A549 cell populations were selected with 2 µg/mL puromycin for one week and knockdown efficiency was identified by western blotting. shRNA sequences used in the study are listed in the follow. RRM1-shRNA-F: CCGG CCTG CTCA GATC ACCA TGAA ACTC GAGT TTCA TGGT GATC TGAG CAGG TTTT TG. RRM1-shRNA-R: AATT CAAA AACC TGCT CAGA TCAC CATG AAAC TCGA GTTT CATG GTGA TCTG AGCA GG. Primer sequences for RRM1 overexpression are listed in the follow. RRM1-oe-F: ATGG ACTA CAAA GACG ATGA CGAC AAGA TGCA TGTG ATCA AGCG AGAT G. RRM1-oe-R: TCAG GATC CACA CATC AGAC ATTC.

### siRNAs and primers

All siRNAs and primers were synthesized by TSINGKE (Nanjing, China). The target sequences for siUSP11 are as follows: GAAG AAGC GTTA CTAT GAC. The target sequences for siE2F1 are as follows: CAGA GCAG ATGG TTAT GGT. siRNA transfections were performed at a final concentration of 50 nM unless otherwise specified. The primer sequences for RAD51AP1 are as follows: forward 5’-CGCC TTGG CTTG TCCA GAT-3’ and reverse 5’-GGTG CTAG TGGC ATTT GGAT G-3’.

### Co-immunoprecipitation and western blot

Cell lysates were prepared using cell lysis buffer (Biosharp). Protein samples were incubated with the respective antibody or non-specific immunoglobulin G and protein G-agarose beads (Beyotime, Shanghai, China) overnight at 4 °C with rotation. After centrifugation, the beads were washed five times with PBS buffer. Immuno-complexes were eluted with 50 µL 1×loading sample buffer and heated at 95 °C for 10 min. Protein samples were separated by SDS-PAGE and transferred onto PVDF membranes (Roche, Basel, Switzerland). Membranes were probed with antibodies and visualized using ECL plus (Amersham Bioscience, London, UK).

### Cell viability and colony formation assay

For cell viability assays, cells were seeded at a density of 2 × 10^3^ per well in 96-well plates and analyzed using the CCK-8 assay (Solarbio, Beijing, China) according to the manufacturer’s instructions. For colony formation assays, 1 × 10^3^ cells were plated in 35 mm cell culture plates. After two weeks of growth, colonies were fixed with 4% paraformaldehyde, stained with crystal violet, and counted under a ×100 microscope.

### HR/NHEJ reporter assay

HeLa cells were transfected with DR-GFP/EJ5-GFP, pCBA-I-SecI, and pmCherry to examine the repair of I-SceI-generated DSBs by HR/NHEJ. HR/NHEJ efficiency was determined at 48 h post-transfection by quantifying double-positive (GFP and mCherry-positive) cells using Flow Cytometry (Thermo Fisher). Results were normalized to the control group.

### Immunofluorescence microscopy

Cells were fixed and permeabilized with ice-cold formaldehyde for 10 min, followed by incubation in PBS containing 0.5% or 0.1% Triton X-100 for 30 min, and then washed with PBS. The cells were incubated with primary antibodies overnight at 4 °C. Subsequently, the cells were incubated with the secondary antibody for 60 min at room temperature. After washing three times with PBST, the cells were mounted in a fluorescent mounting medium. Images were acquired using an Olympus fluoview-1000 confocal microscope (Olympus, Tokyo, Japan).

### Prediction of Interaction Domain

Initially, the PDB file of the complex was generated using the AlphaFold2 online server (https://colab.research.google.com/github/sokrypton/ColabFold/blob/main/AlphaFold2.ipynb). Then, the PDB file was analyzed using the PDBePISA online server (https://www.ebi.ac.uk/msd-srv/prot_int/cgi-bin/piserver).

### Statistical analysis

Experiments were conducted independently three times, and the data are presented as the mean ± standard error of the mean. Statistical comparisons between two groups were performed using Student’s t-test, while comparisons among multiple groups were conducted using two-way ANOVA. A *p*-value < 0.05 was considered statistically significant. The levels of significance were denoted as follows: *p < 0.05, **p < 0.01, ***p < 0.001, ****p < 0.0001, and ns = not significant.

## Supplementary information

Supplementary Figures and Legends

Full and uncropped western blots

## Data Availability

All other data supporting the findings of this study are available from the corresponding author upon reasonable request.

## References

[1] Wang Y, Deng O, Feng Z, Du Z, Xiong X, Lai J, et al. RNF126 promotes homologous recombination via regulation of E2F1-mediated BRCA1 expression. Oncogene. 2016;35:1363–72. 26234677 10.1038/onc.2015.198 PMC4740281

[2] Cao B, Wu X, Zhou J, Wu H, Liu L, Zhang Q, et al. Nick-seq for single-nucleotide resolution genomic maps of DNA modifications and damage. Nucleic Acids Res. 2020;48:6715–25. 32484547 10.1093/nar/gkaa473 PMC7337925

[3] Sabourin M, Osheroff N. Sensitivity of human type II topoisomerases to DNA damage: stimulation of enzyme-mediated DNA cleavage by abasic, oxidized and alkylated lesions[J]. Nucleic Acids Res. 2000;28:1947–54. 10756196 10.1093/nar/28.9.1947 PMC103304

[4] Shen H, Li Z. DNA Double-Strand Break Repairs and Their Application in Plant DNA Integration. Genes (Basel), 2022, 13. 10.3390/genes13020322 PMC8871565 35205367

[5] Ortega P, Merida-Cerro JA, Rondon AG, Gomez-Gonzalez B, Aguilera A. DNA-RNA hybrids at DSBs interfere with repair by homologous recombination. Elife. 2021, 10. 10.7554/eLife.69881 PMC8289408 34236317

[6] Carusillo A, Mussolino C. DNA Damage: From Threat to Treatment. Cells. 2020, 9. 10.3390/cells9071665 PMC7408370 32664329

[7] Cuella-Martin R, Oliveira C, Lockstone HE, Snellenberg S, Grolmusova N, Chapman JR. 53BP1 Integrates DNA Repair and p53-Dependent Cell Fate Decisions via Distinct Mechanisms. Mol Cell. 2016;64:51–64. 27546791 10.1016/j.molcel.2016.08.002 PMC5065530

[8] Martini E, Keeney S. Sex and the single (double-strand) break. Mol Cell. 2002;9:700–2. 11983162 10.1016/s1097-2765(02)00512-9

[9] Carrasco C, Dillingham MS, Moreno-Herrero F. Single molecule approaches to monitor the recognition and resection of double-stranded DNA breaks during homologous recombination. DNA Repair (Amst). 2014;20:119–29. 24569169 10.1016/j.dnarep.2014.02.002

[10] O’connor MJ. Targeting the DNA Damage Response in Cancer. Mol Cell. 2015;60:547–60. 26590714 10.1016/j.molcel.2015.10.040

[11] Ceccaldi R, Rondinelli B, D’andrea AD. Repair Pathway Choices and Consequences at the Double-Strand Break. Trends Cell Biol. 2016;26:52–64. 26437586 10.1016/j.tcb.2015.07.009 PMC4862604

[12] Long MJC, Van Hall-Beauvais A, Aye Y. The more the merrier: how homo-oligomerization alters the interactome and function of ribonucleotide reductase. Curr Opin Chem Biol. 2020;54:10–18. 31734537 10.1016/j.cbpa.2019.09.003 PMC7131891

[13] Aye Y, Li M, Long MJ, Weiss RS. Ribonucleotide reductase and cancer: biological mechanisms and targeted therapies. Oncogene. 2015;34:2011–21. 24909171 10.1038/onc.2014.155

[14] Tian J, Han S. Role of RRM1 in the Treatment and Prognosis of Advanced Non-small Cell Lung Cancer. Zhongguo Fei Ai Za Zhi. 2015;18:381–6. 26104896 10.3779/j.issn.1009-3419.2015.06.09 PMC5999903

[15] Zhu CM, Lian XY, Bi YH, Hu CC, Liang YW, Li QS. Prognostic value of ribonucleotide reductase subunit M1 (RRM1) in non-small cell lung cancer: A meta-analysis. Clin Chim Acta. 2018;485:67–73. 29803896 10.1016/j.cca.2018.05.042

[16] Jordheim LP, Seve P, Tredan O, Dumontet C. The ribonucleotide reductase large subunit (RRM1) as a predictive factor in patients with cancer. Lancet Oncol. 2011;12:693–702. 21163702 10.1016/S1470-2045(10)70244-8

[17] Sagawa M, Ohguchi H, Harada T, Samur MK, Tai YT, Munshi NC, et al. Ribonucleotide Reductase Catalytic Subunit M1 (RRM1) as a Novel Therapeutic Target in Multiple Myeloma. Clin Cancer Res. 2017;23:5225–37. 28442502 10.1158/1078-0432.CCR-17-0263 PMC5581671

[18] Gautam A, Bepler G. Suppression of lung tumor formation by the regulatory subunit of ribonucleotide reductase. Cancer Res. 2006;66:6497–502. 16818620 10.1158/0008-5472.CAN-05-4462

[19] Gao Y, Chen B, Wang R, Xu A, Wu L, Lu H, et al. Knockdown of RRM1 in tumor cells promotes radio-/chemotherapy induced ferroptosis by regulating p53 ubiquitination and p21-GPX4 signaling axis. Cell Death Discov. 2022;8:343. 35915092 10.1038/s41420-022-01140-z PMC9343379

[20] Fugger K, Bajrami I, Silva Dos Santos M, Young SJ, Kunzelmann S, Kelly G, et al. Targeting the nucleotide salvage factor DNPH1 sensitizes BRCA-deficient cells to PARP inhibitors. Science. 2021;372:156–65. 33833118 10.1126/science.abb4542 PMC7610649

[21] Verma P, Zhou Y, Cao Z, Deraska PV, Deb M, Arai E, et al. ALC1 links chromatin accessibility to PARP inhibitor response in homologous recombination-deficient cells. Nat Cell Biol. 2021;23:160–71. 33462394 10.1038/s41556-020-00624-3 PMC7880902

[22] Zhou J, Tong F, Zhao J, Cui X, Wang Y, Wang G, et al. Identification of the E2F1-RAD51AP1 axis as a key factor in MGMT-methylated GBM TMZ resistance. Cancer Biol Med. 2023;20:385–400. 37283490 10.20892/j.issn.2095-3941.2023.0011 PMC10246439

[23] Iwanaga R, Komori H, Ishida S, Okamura N, Nakayama K, Nakayama KI, et al. Identification of novel E2F1 target genes regulated in cell cycle-dependent and independent manners. Oncogene. 2006;25:1786–98. 16288221 10.1038/sj.onc.1209210

[24] Koren I, Timms RT, Kula T, Xu Q, Li MZ, Elledge SJ. The Eukaryotic Proteome Is Shaped by E3 Ubiquitin Ligases Targeting C-Terminal Degrons. Cell. 2018;173:1622–35.e14. 29779948 10.1016/j.cell.2018.04.028 PMC6003881

[25] Qiao L, Zhang Q, Sun Z, Liu Q, Wu Z, Hu W, et al. The E2F1/USP11 positive feedback loop promotes hepatocellular carcinoma metastasis and inhibits autophagy by activating ERK/mTOR pathway. Cancer Lett. 2021;514:63–78. 34044068 10.1016/j.canlet.2021.05.015

[26] Wang D, Zhao J, Li S, Wei J, Nan L, Mallampalli RK, et al. Phosphorylated E2F1 is stabilized by nuclear USP11 to drive Peg10 gene expression and activate lung epithelial cells. J Mol Cell Biol. 2018;10:60–73. 28992046 10.1093/jmcb/mjx034 PMC6075510

[27] Garcia A, Rodriguez Matas JF, Raimondi MT. Modeling of the mechano-chemical behaviour of the nuclear pore complex: current research and perspectives. Integr Biol (Camb). 2016;8:1011–21. 27713975 10.1039/c6ib00153j PMC5166569

[28] Oka M, Yoneda Y. Importin alpha: functions as a nuclear transport factor and beyond. Proc Jpn Acad Ser B Phys Biol Sci. 2018;94:259–74. 30078827 10.2183/pjab.94.018 PMC6117492

[29] Knockenhauer KE, Schwartz TU. The Nuclear Pore Complex as a Flexible and Dynamic Gate. Cell. 2016;164:1162–71. 26967283 10.1016/j.cell.2016.01.034 PMC4788809

[30] Uhlin U, Eklund H. Structure of ribonucleotide reductase protein R1. Nature. 1994;370:533–9. 8052308 10.1038/370533a0

[31] Eriksson M, Uhlin U, Ramaswamy S, Ekberg M, Regnstrom K, Sjoberg BM, et al. Binding of allosteric effectors to ribonucleotide reductase protein R1: reduction of active-site cysteines promotes substrate binding. Structure. 1997;5:1077–92. 9309223 10.1016/s0969-2126(97)00259-1

[32] Greene BL, Kang G, Cui C, Bennati M, Nocera DG, Drennan CL, et al. Ribonucleotide Reductases: Structure, Chemistry, and Metabolism Suggest New Therapeutic Targets. Annu Rev Biochem. 2020;89:45–75. 32569524 10.1146/annurev-biochem-013118-111843 PMC7316142

[33] Rose HR, Maggiolo AO, Mcbride MJ, Palowitch GM, Pandelia ME, Davis KM, et al. Structures of Class Id Ribonucleotide Reductase Catalytic Subunits Reveal a Minimal Architecture for Deoxynucleotide Biosynthesis. Biochemistry. 2019;58:1845–60. 30855138 10.1021/acs.biochem.8b01252 PMC6456427

[34] Reichard P, Baldesten A, Rutberg L. Formation of deoxycytidine phosphates from cytidine phosphates in extracts from Escherichia coli. J Biol Chem. 1961;236:1150–7. 13740426

[35] Cotruvo JA, Stubbe J. Class I ribonucleotide reductases: metallocofactor assembly and repair in vitro and in vivo. Annu Rev Biochem. 2011;80:733–67. 21456967 10.1146/annurev-biochem-061408-095817 PMC4703083

[36] Minnihan EC, Nocera DG, Stubbe J. Reversible, long-range radical transfer in E. coli class Ia ribonucleotide reductase. Acc Chem Res. 2013;46:2524–35. 23730940 10.1021/ar4000407 PMC3823682

[37] Hofer A, Crona M, Logan DT, Sjoberg BM. DNA building blocks: keeping control of manufacture. Crit Rev Biochem Mol Biol. 2012;47:50–63. 22050358 10.3109/10409238.2011.630372 PMC3267527

[38] Chen B, Ge T, Jian M, Chen L, Fang Z, He Z, et al. Transmembrane nuclease NUMEN/ENDOD1 regulates DNA repair pathway choice at the nuclear periphery. Nat Cell Biol. 2023;25:1004–16. 37322289 10.1038/s41556-023-01165-1

[39] Oza P, Jaspersen SL, Miele A, Dekker J, Peterson CL. Mechanisms that regulate localization of a DNA double-strand break to the nuclear periphery. Genes Dev. 2009;23:912–27. 19390086 10.1101/gad.1782209 PMC2675867

[40] Tsouroula K, Furst A, Rogier M, Heyer V, Maglott-Roth A, Ferrand A, et al. Temporal and Spatial Uncoupling of DNA Double Strand Break Repair Pathways within Mammalian Heterochromatin. Mol Cell. 2016;63:293–305. 27397684 10.1016/j.molcel.2016.06.002

[41] Meschini R, Morucci E, Berni A, Lopez-Martinez W, Palitti F. Role of chromatin structure modulation by the histone deacetylase inhibitor trichostatin A on the radio-sensitivity of ataxia telangiectasia. Mutat Res. 2015;777:52–9. 25942615 10.1016/j.mrfmmm.2015.04.009

[42] Fontana GA, Hess D, Reinert JK, Mattarocci S, Falquet B, Klein D, et al. Rif1 S-acylation mediates DNA double-strand break repair at the inner nuclear membrane. Nat Commun. 2019;10:2535. 31182712 10.1038/s41467-019-10349-z PMC6557901

[43] Nagai S, Dubrana K, Tsai-Pflugfelder M, Davidson MB, Roberts TM, Brown GW, et al. Functional targeting of DNA damage to a nuclear pore-associated SUMO-dependent ubiquitin ligase. Science. 2008;322:597–602. 18948542 10.1126/science.1162790 PMC3518492

[44] Mathews CK. DNA precursor metabolism and genomic stability. FASEB J. 2006;20:1300–14. 16816105 10.1096/fj.06-5730rev

[45] Rampazzo C, Miazzi C, Franzolin E, Pontarin G, Ferraro P, Frangini M, et al. Regulation by degradation, a cellular defense against deoxyribonucleotide pool imbalances. Mutat Res. 2010;703:2–10. 20561600 10.1016/j.mrgentox.2010.06.002

[46] Qu J, Sun W, Zhong J, Lv H, Zhu M, Xu J, et al. Phosphoglycerate mutase 1 regulates dNTP pool and promotes homologous recombination repair in cancer cells. J Cell Biol. 2017;216:409–24. 28122957 10.1083/jcb.201607008 PMC5294784

[47] Burkhalter MD, Roberts SA, Havener JM, Ramsden DA. Activity of ribonucleotide reductase helps determine how cells repair DNA double strand breaks. DNA Repair (Amst). 2009;8:1258–63. 19713159 10.1016/j.dnarep.2009.07.009 PMC2763971

