# Supplementary material for: RRM1 promotes homologous recombination and radio/chemo-sensitivity via enhancing USP11 and E2F1-mediated RAD51AP1 transcription
Source: Cell Death Discov. 2024 Dec 18;10:496. doi: 10.1038/s41420-024-02267-x (PMC11655868; doi:10.1038/s41420-024-02267-x)
Supplement: Supplementary file 1 — Supplementary Figures and Legends [file 41420_2024_2267_MOESM1_ESM.docx]

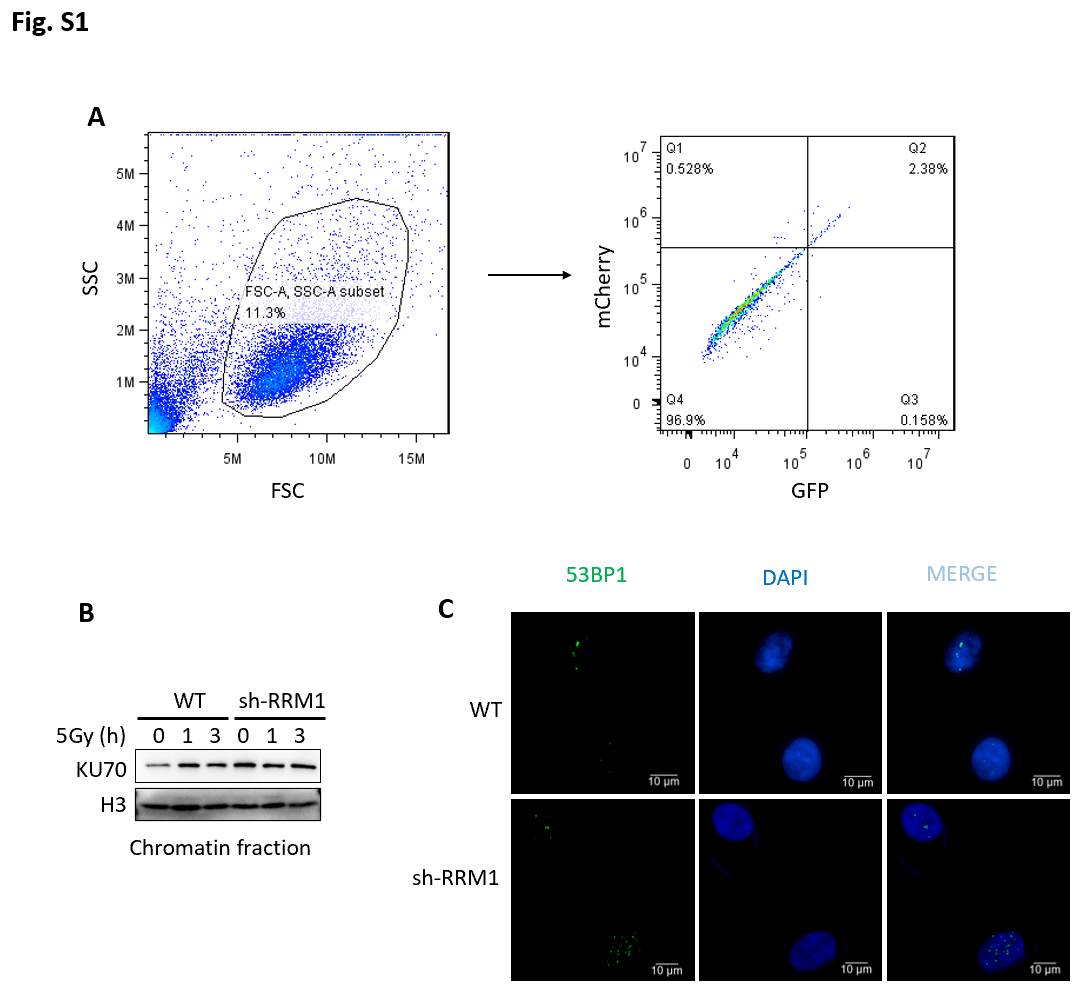


-15 kDa

-70 kDa

**Fig. S1 A** Example of flow cytometry gating strategy used in DSB reporter experiments of Fig. 1E. **B** Chromatin separation experiment used to detect chromatin recruitment of KU70 in Hela wild-type and RRM1 knockdown cells. **C** Immunofluorescence staining was used to detect the formation of 53BP1 lesions in Hela wild-type and RRM1 knockdown cells after DNA damage.


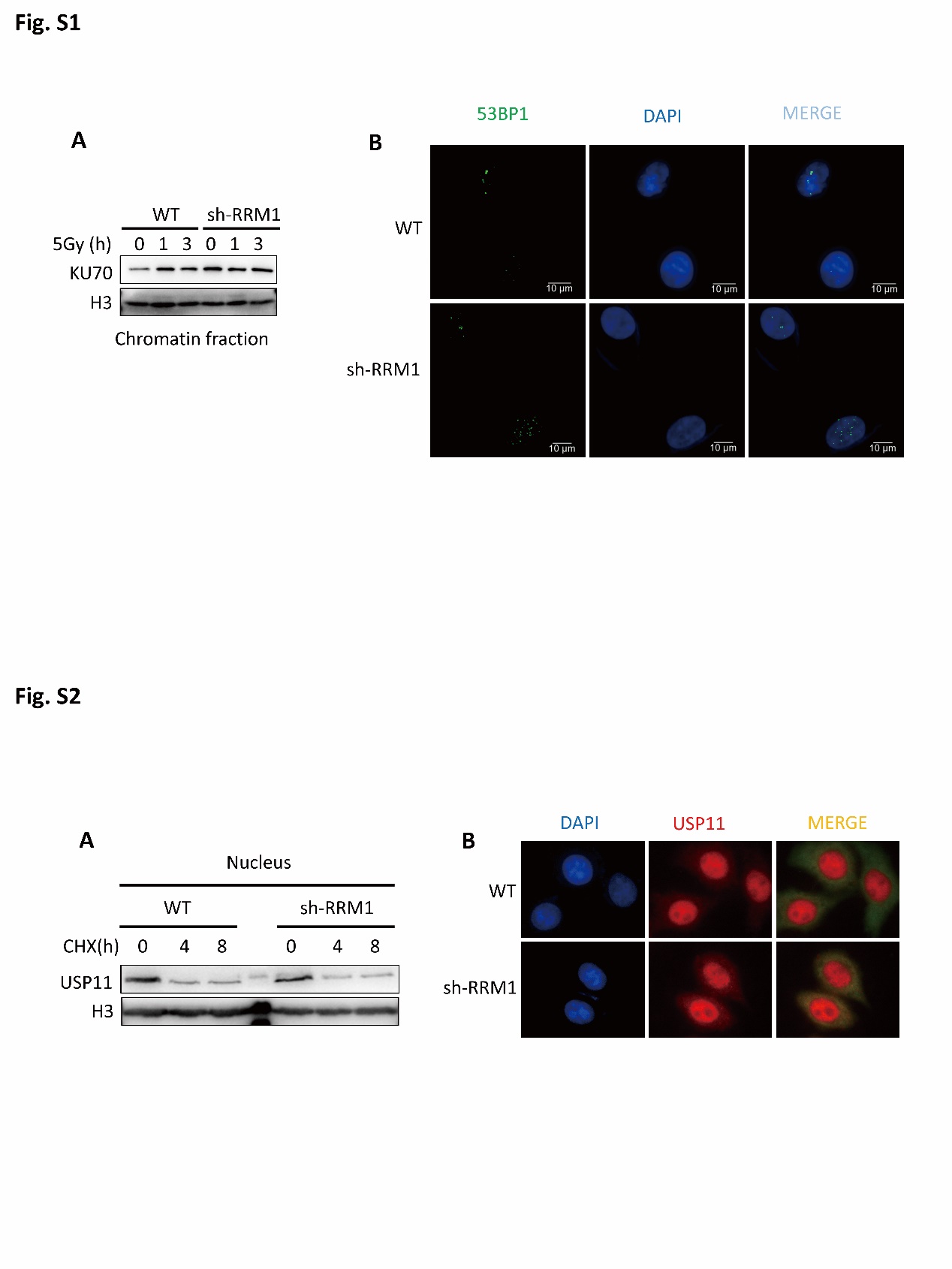


-15 kDa

-110 kDa

15 μm

**Fig. S2 A** CHX-treated Hela cells collected samples every 4 hours, extracted nuclear components through nuclear-cytoplasmic separation experiments, and then detected the amount of USP11 protein in the nucleus. **B** Immunofluorescence staining was used to detect the location of USP11.


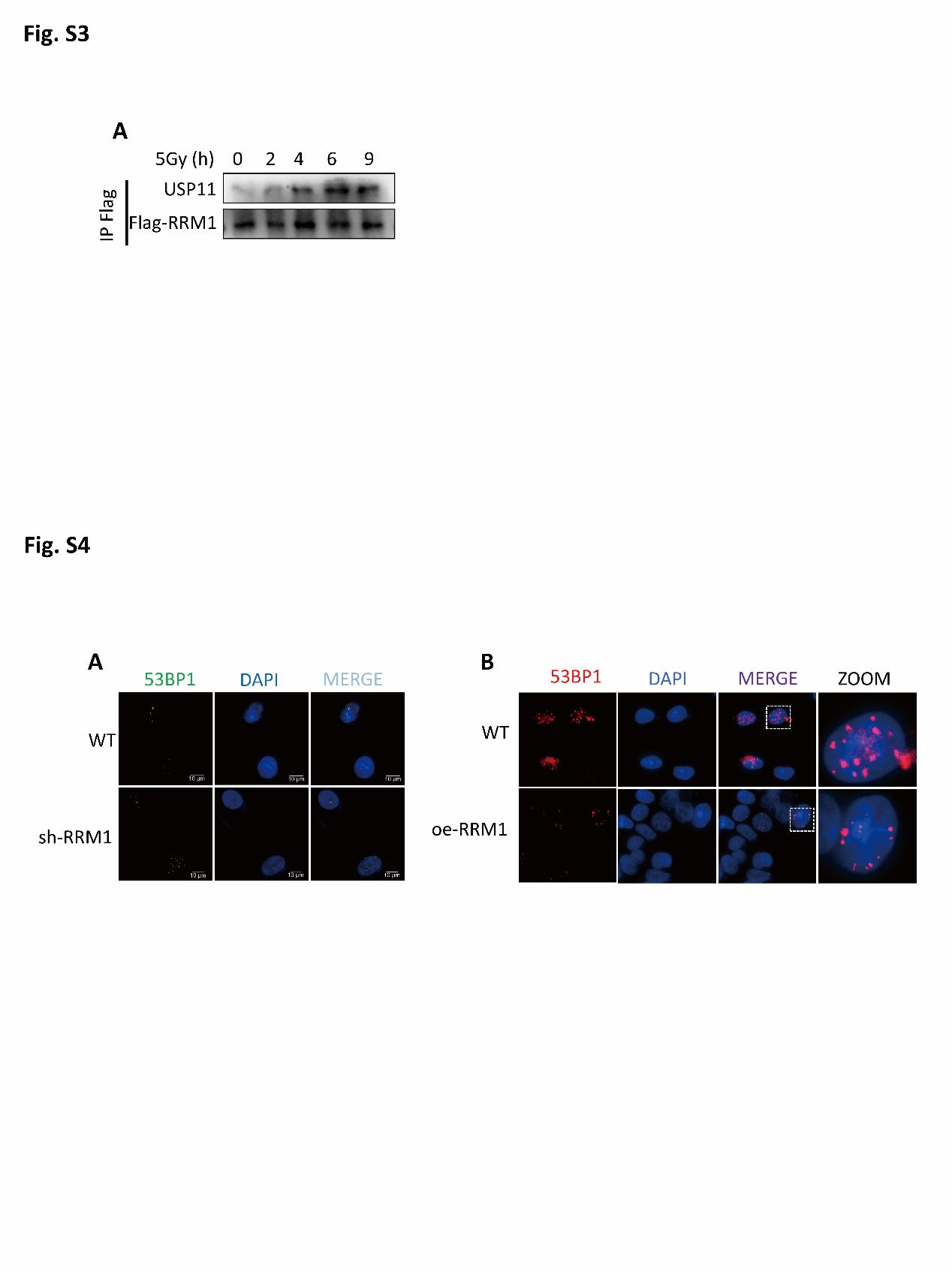
**Fig. S3 A** After labeling RRM1 with Flag tags and treating Hela cells with 5Gy, the protein levels of USP11 co-precipitated with anti-Flag antibodies were detected at different time points.

-90 kDa

-110 kDa


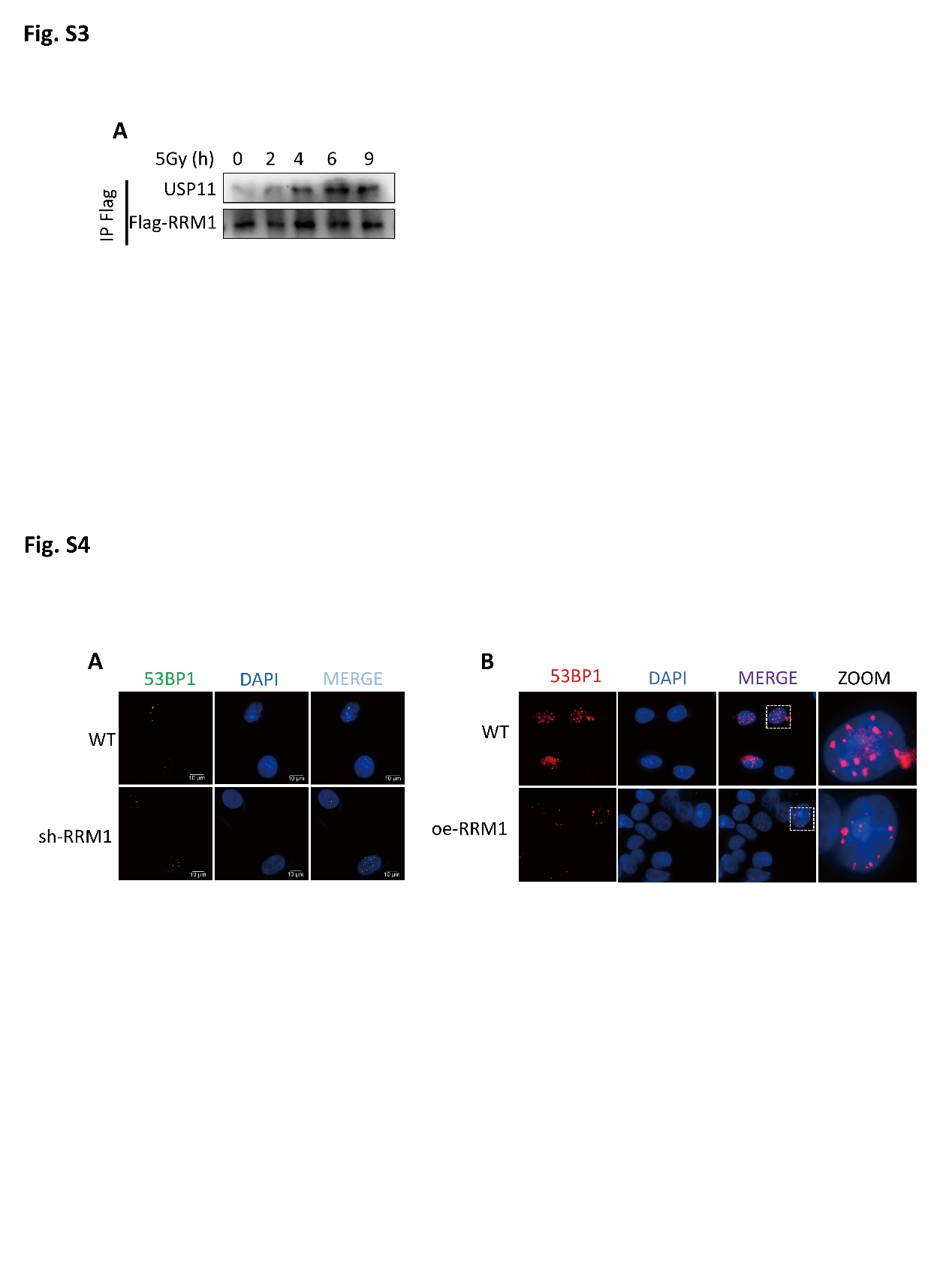


15 μm

**Fig. S4 A-B** Immunofluorescence staining was used to detect the formation of 53BP1 foci in Hela wild-type, RRM1 knockdown, and RRM1 overexpression cells after IR.

**Fig. S6**

**Fig. S5**


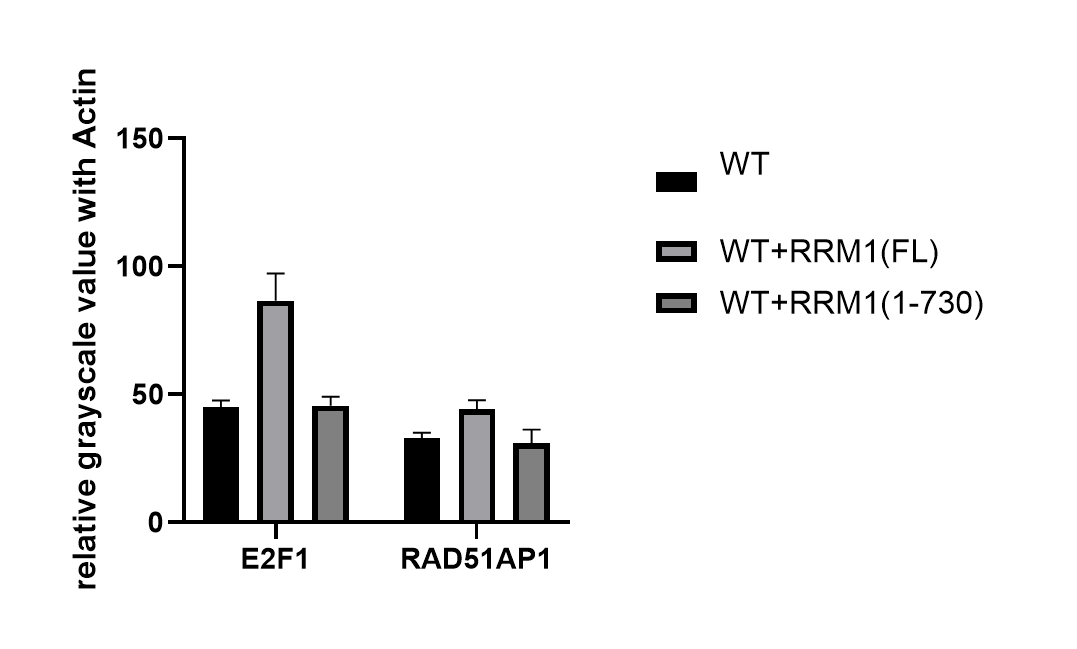

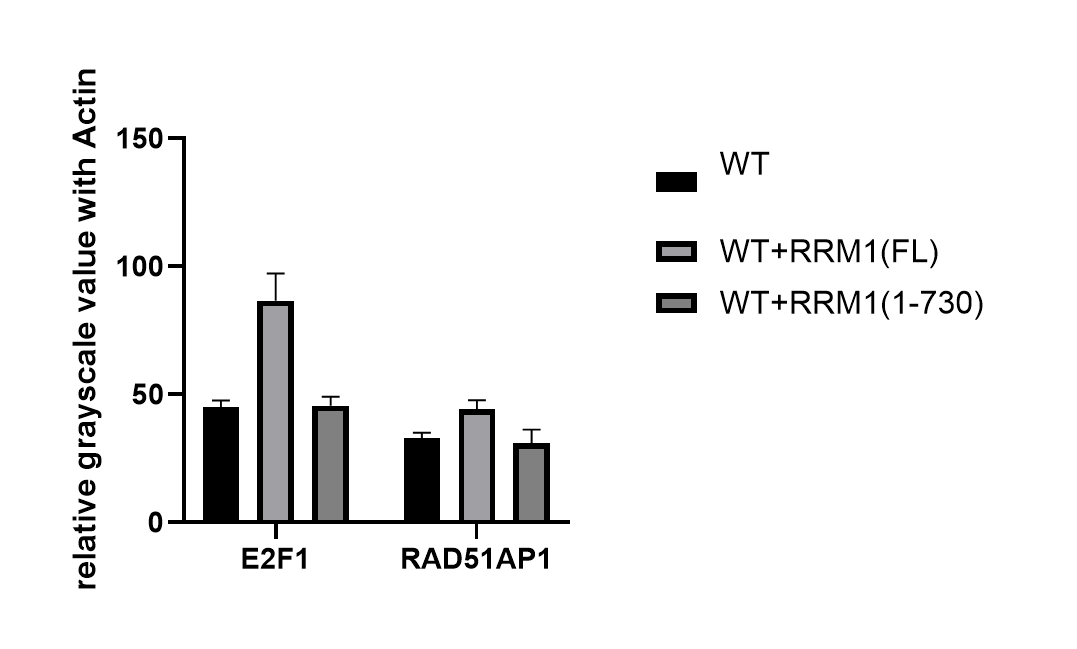

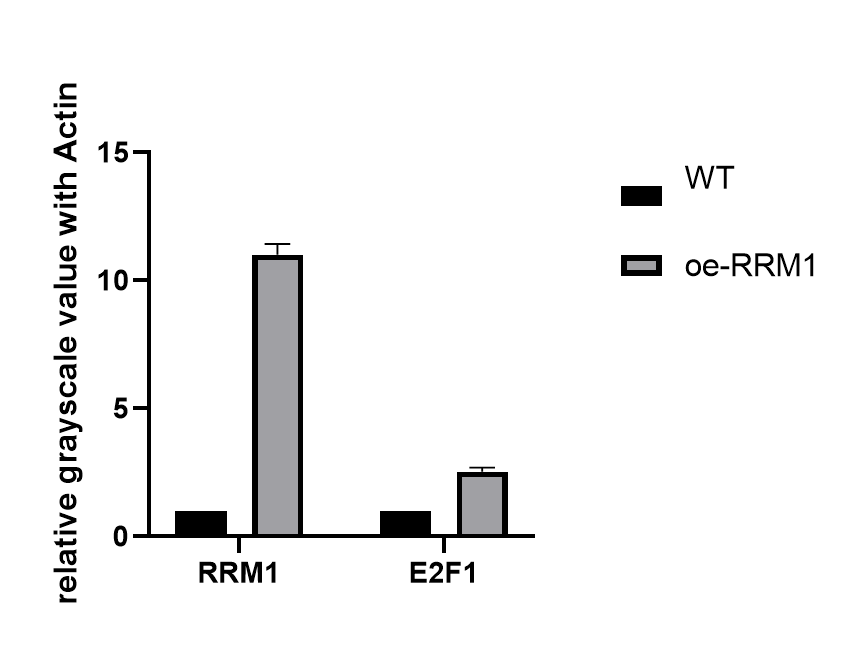

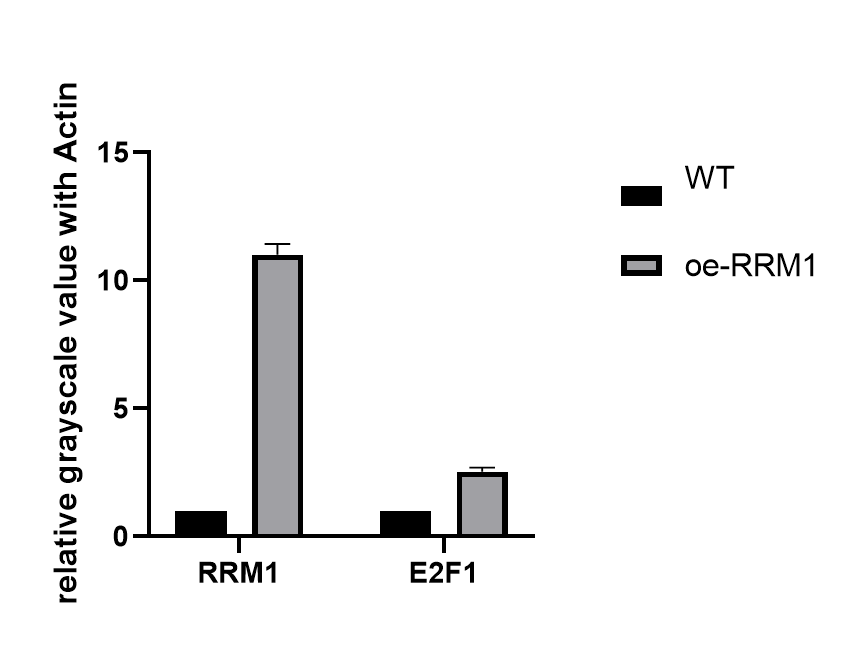


**Fig. S5** Use Image J to detect the grayscale values of Actin, RRM1, and E2F1 bands. Then analyze the relative values of RRM1and E2F1 to Actin.

**Fig. S6** Use Image J to detect the grayscale values of Actin, E2F1, and RAD51AP1 bands. Then analyze the relative values of E2F1 and RAD51AP1 to Actin.

**Fig. S7**


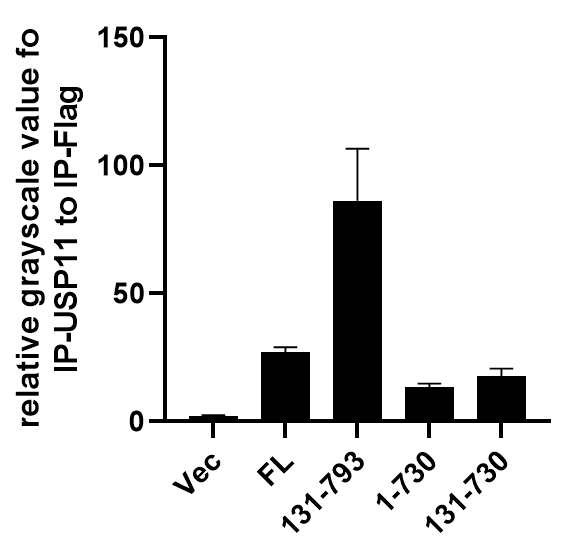


**Fig. S6** Use Image J to detect the grayscale values of IP-USP11 and IP-Flag bands. Then analyze the relative values of IP-USP11 to Actin.
