# Supplementary material for: RRM1 promotes homologous recombination and radio/chemo-sensitivity via enhancing USP11 and E2F1-mediated RAD51AP1 transcription
Source: Cell Death Discov. 2024 Dec 18;10:496. doi: 10.1038/s41420-024-02267-x (PMC11655868; doi:10.1038/s41420-024-02267-x)
Supplement: Supplementary file 2 — Full and uncropped western blots [file 41420_2024_2267_MOESM2_ESM.docx]

**Full and uncropped western blots**

**Fig. SM1**

A:(Actin, RRM1)


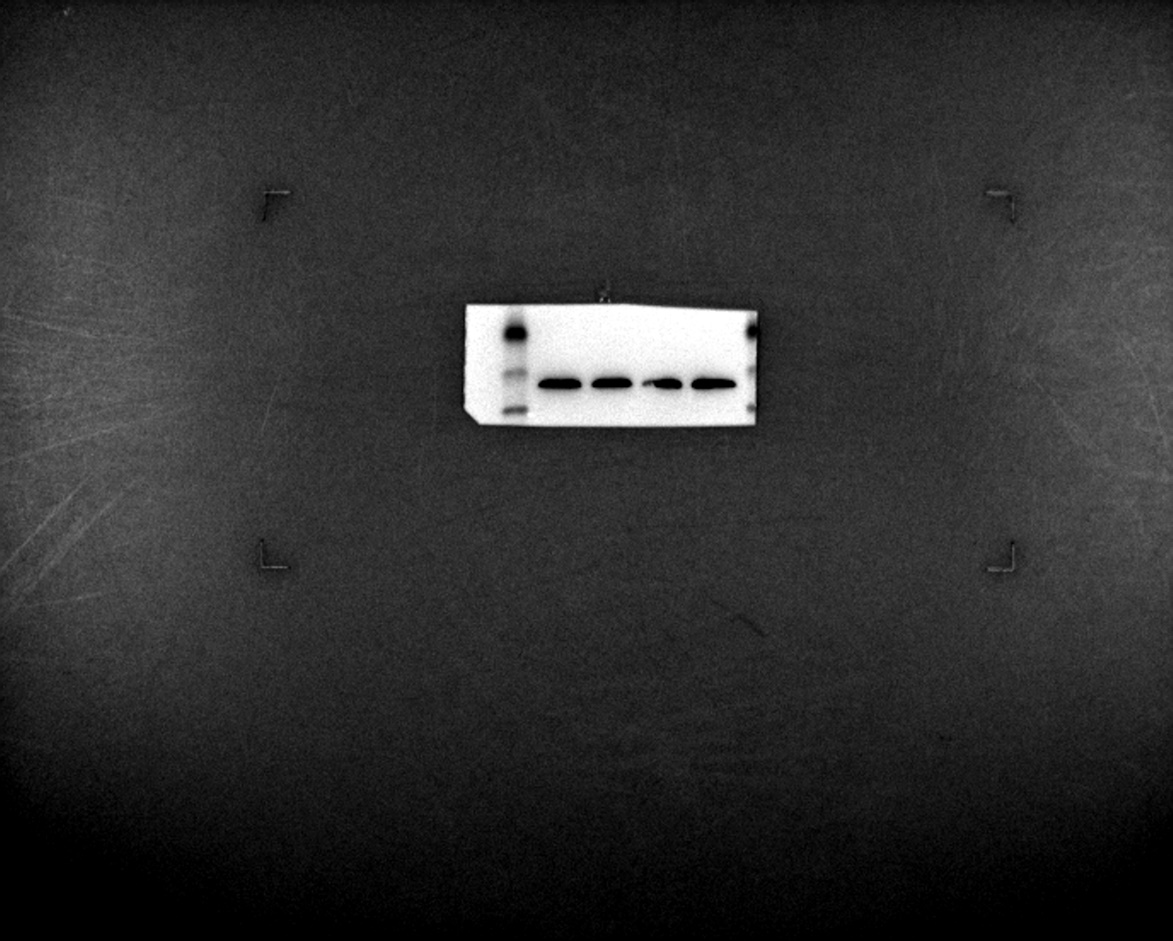

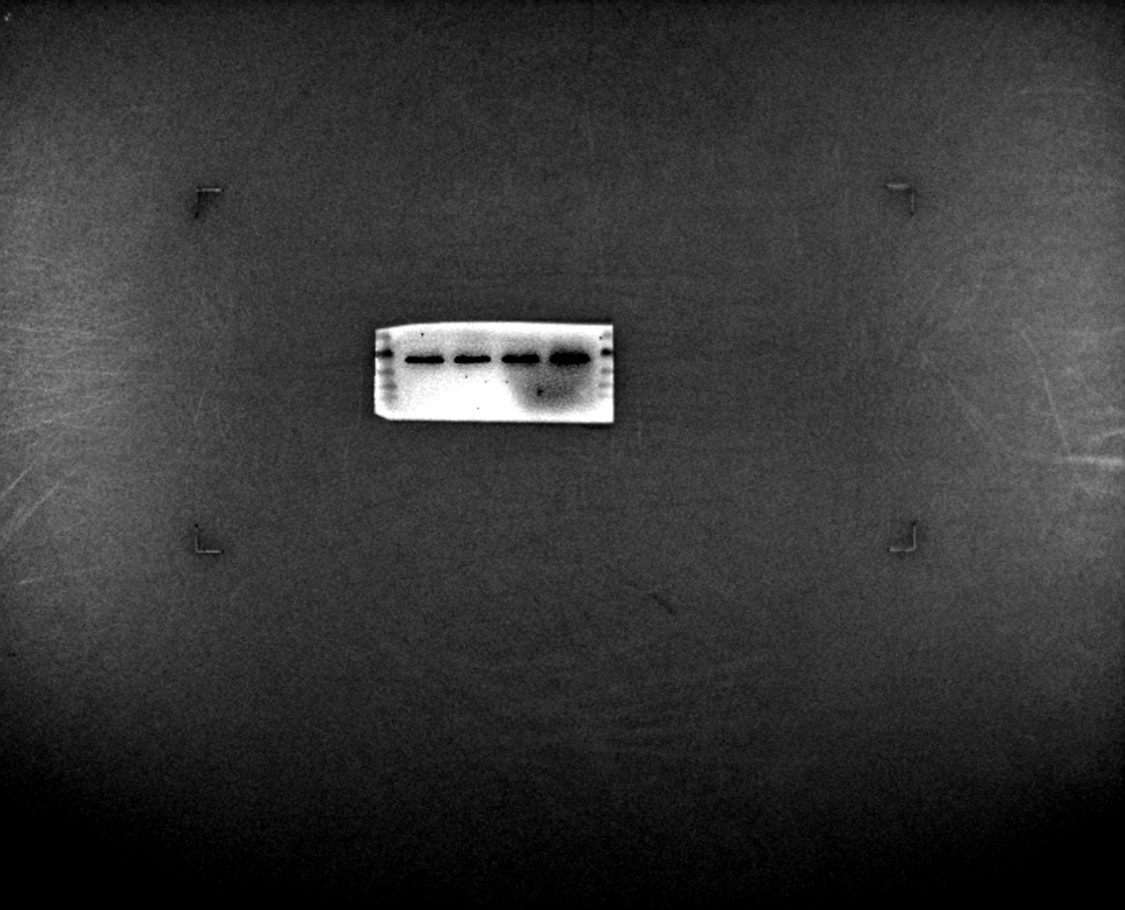


B:(Actin, RRM1）


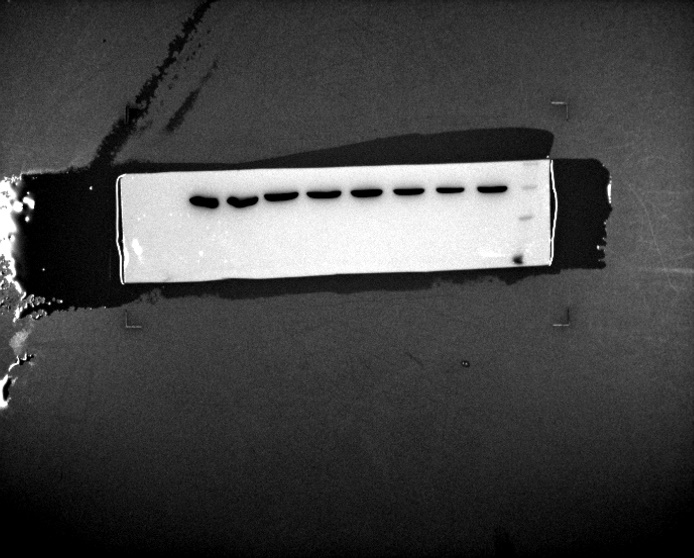

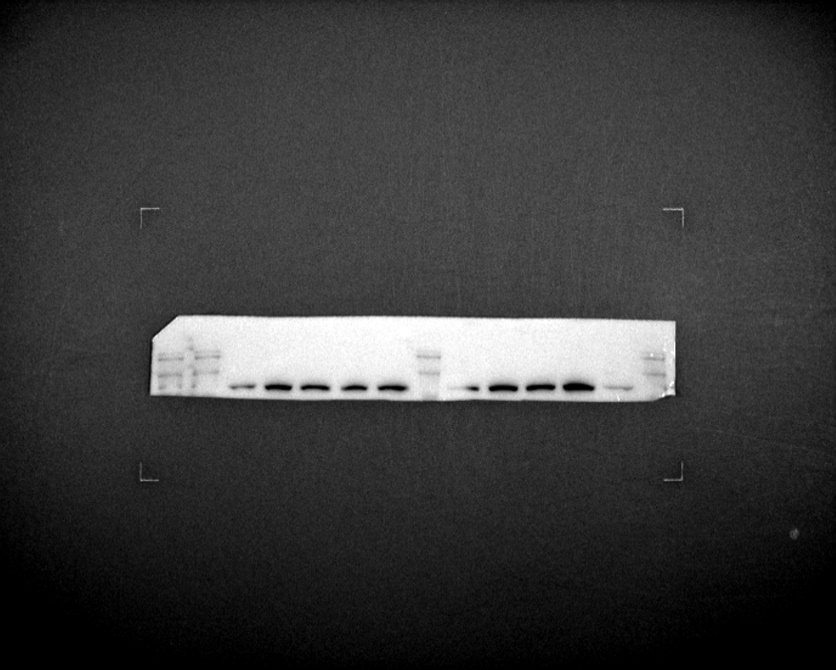


C:(Actin, γH2AX)


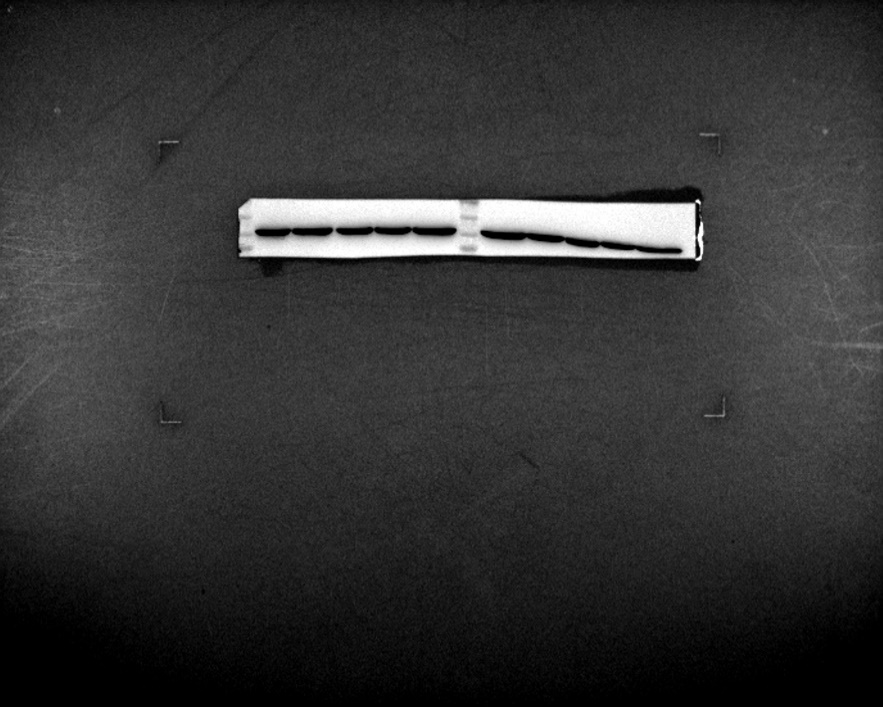

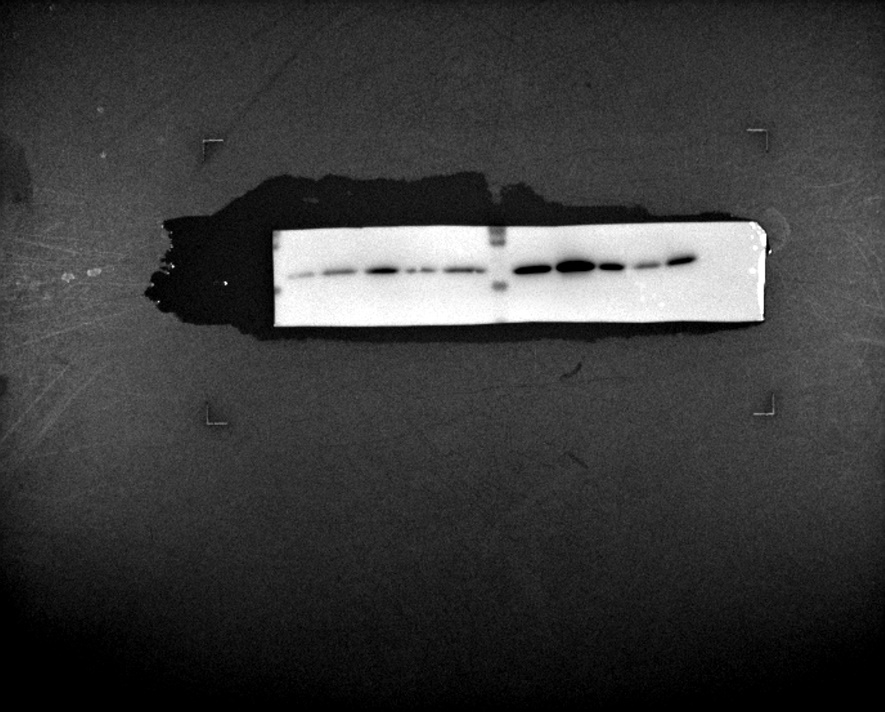


G:(Actin, H3, RAD51)


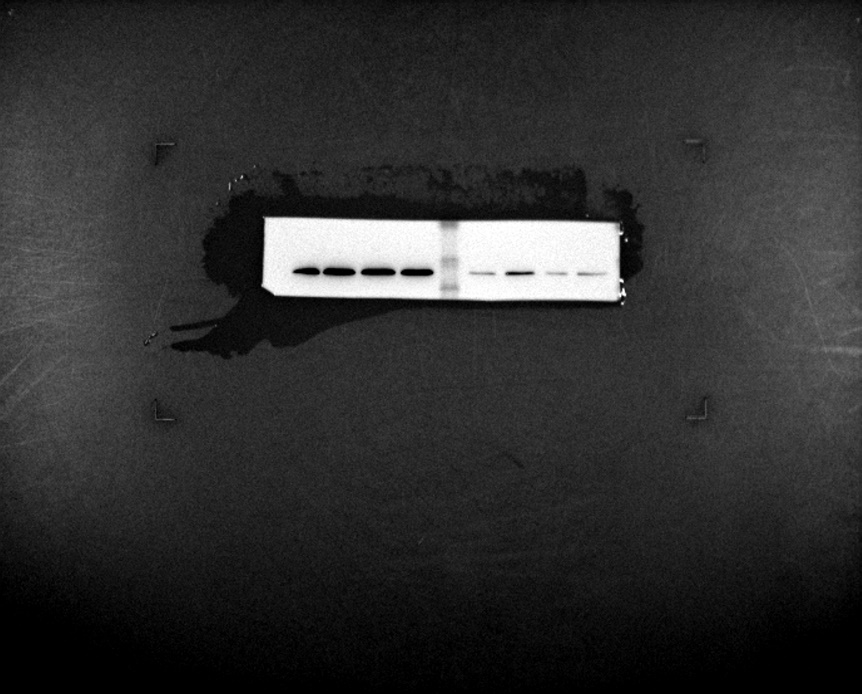

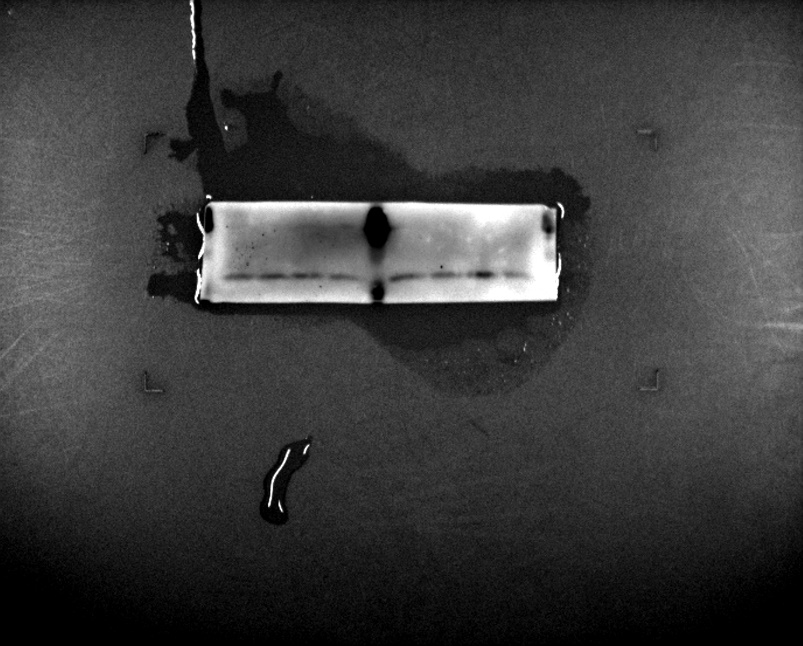

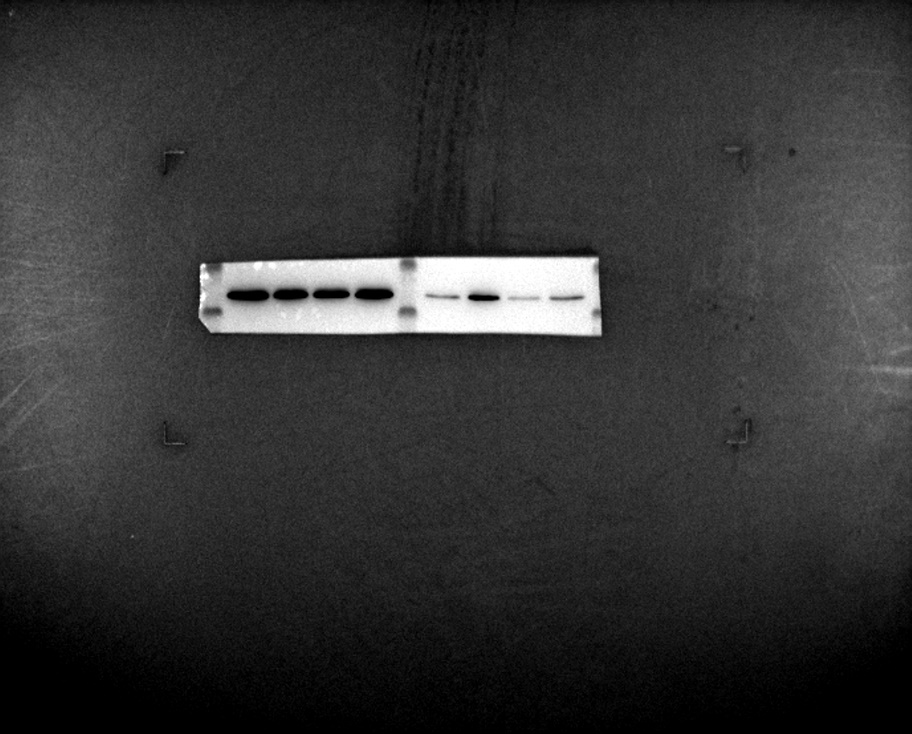


**Fig. SM2**

C:(Actin, RRM1, RAD51AP1）


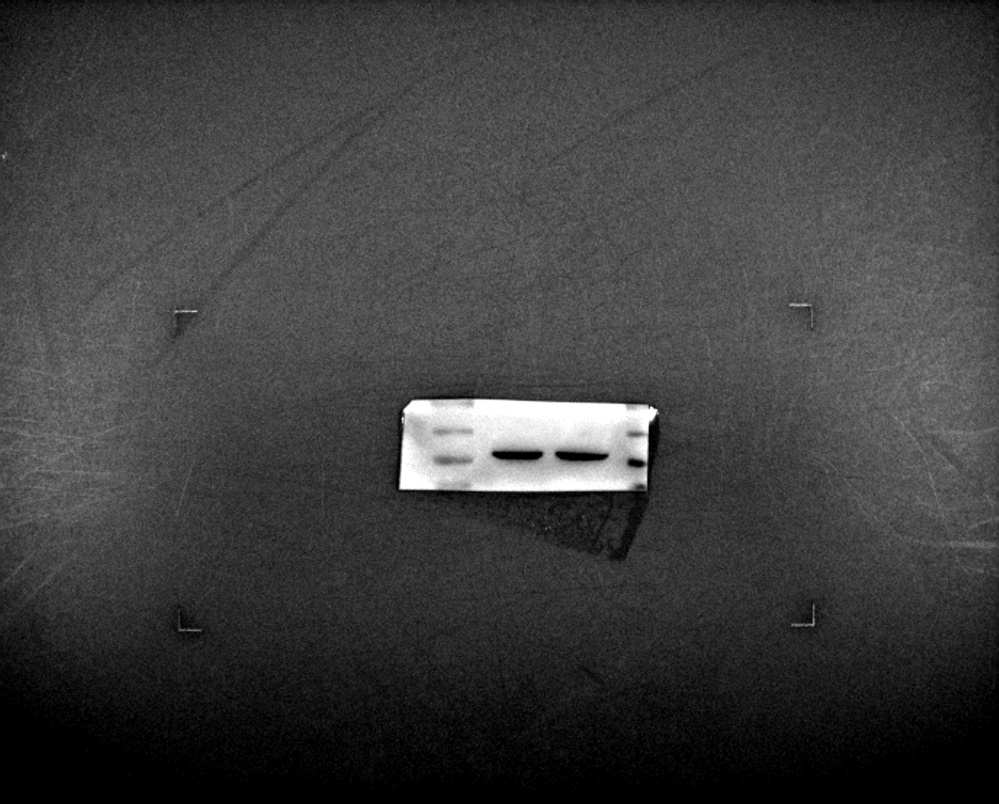

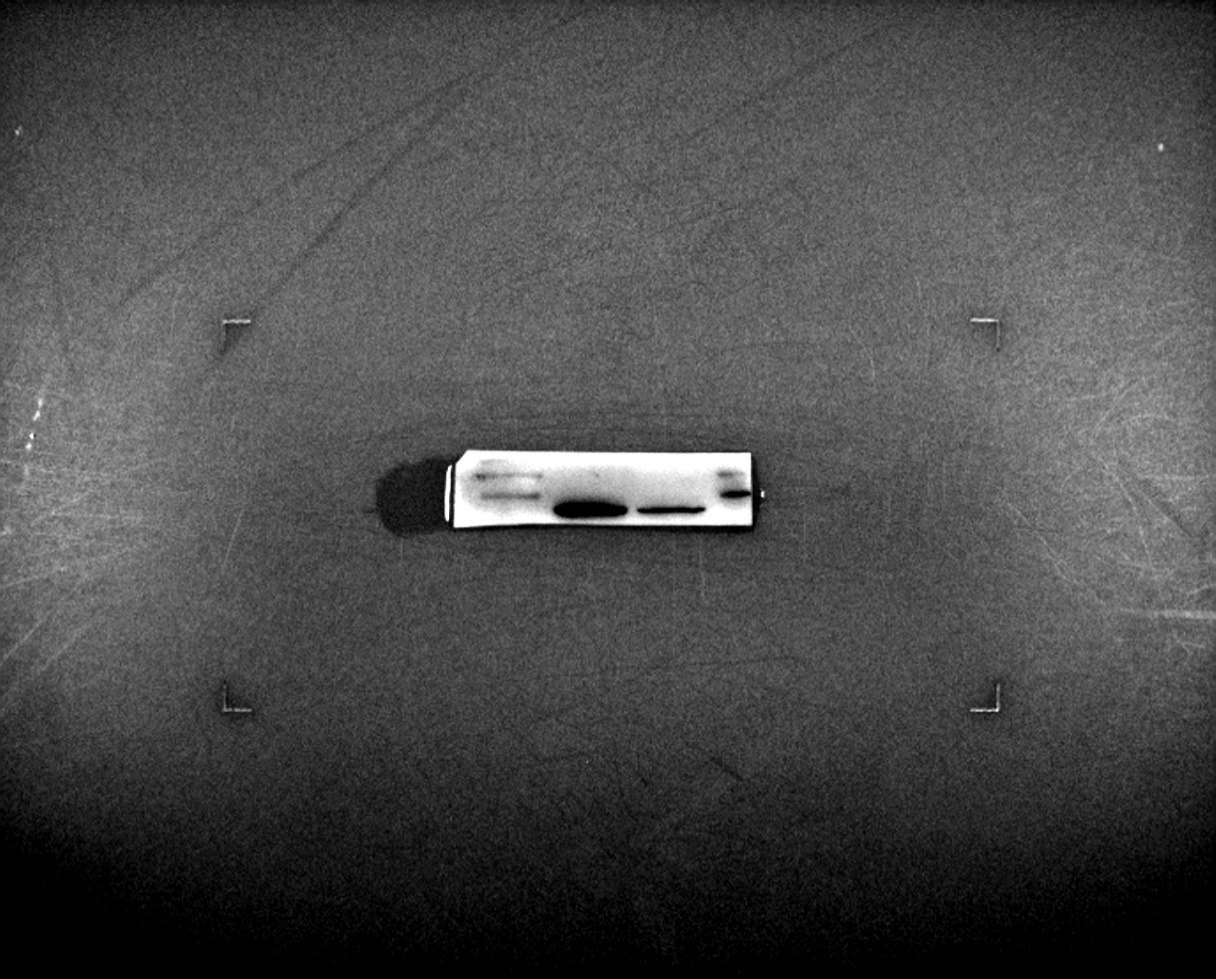

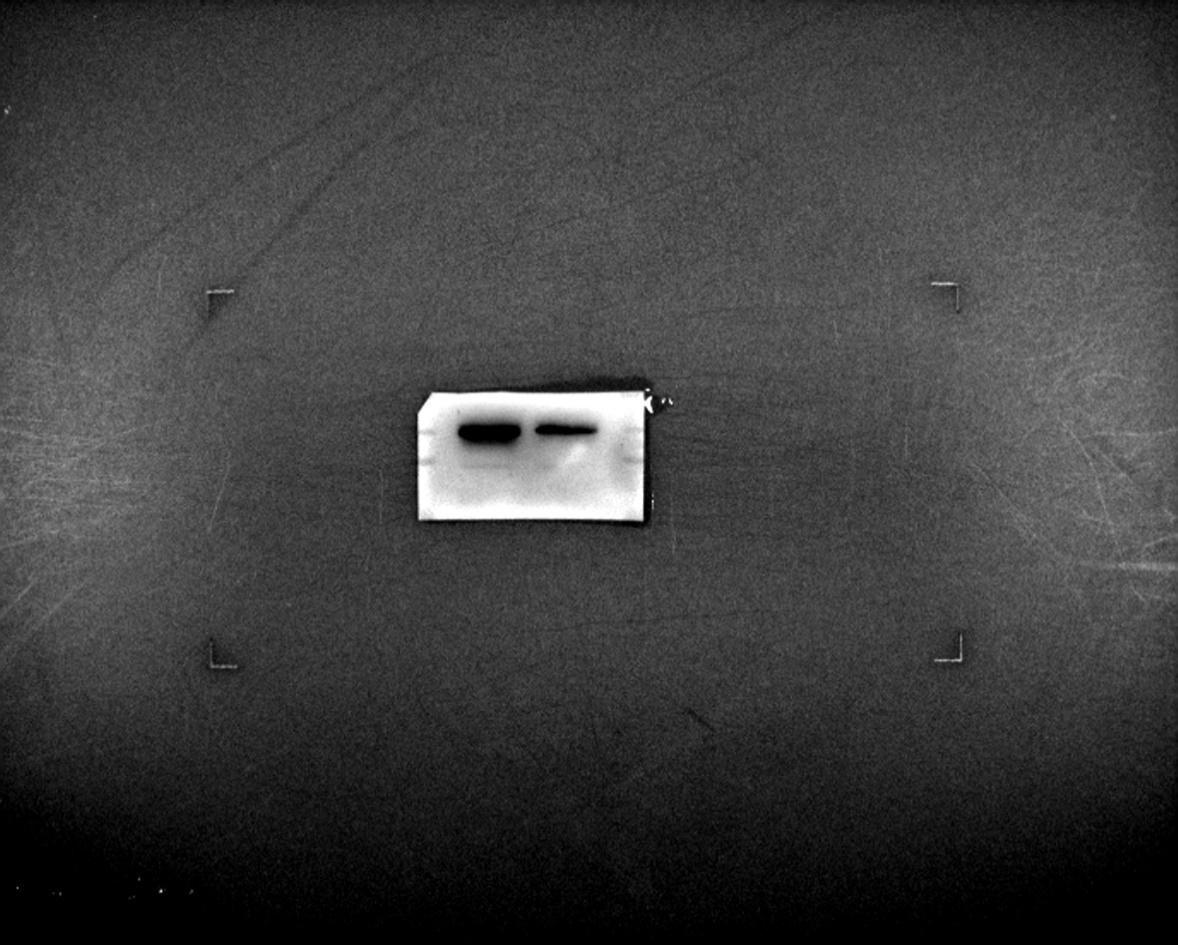


D:(Actin, RRM1, RAD51AP1)


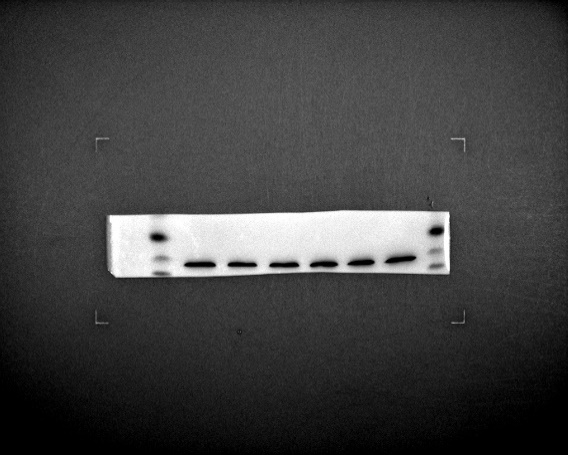

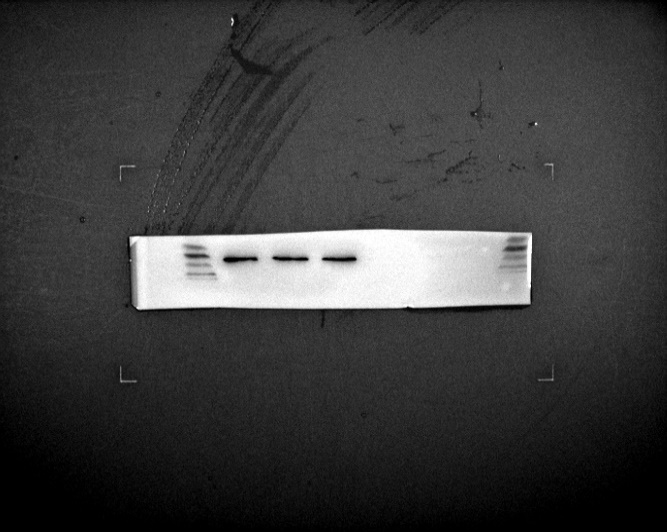

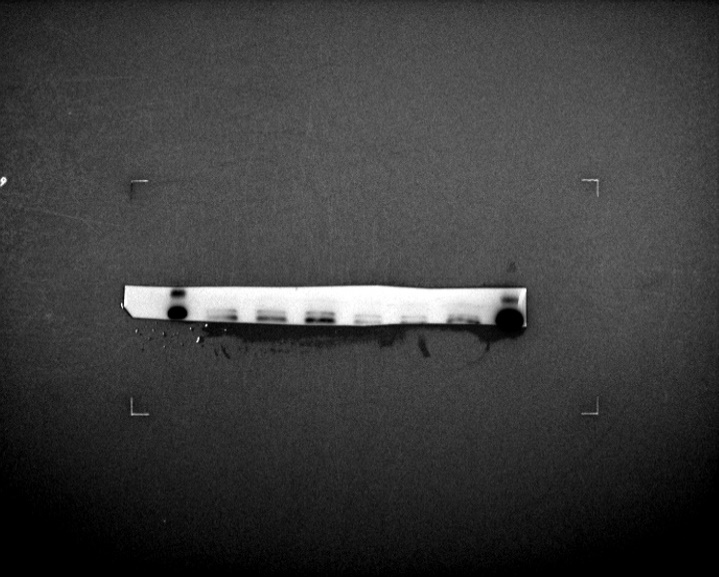


F:(Actin, RRM1, Flag-RRM1, E2F1, RAD51AP1)


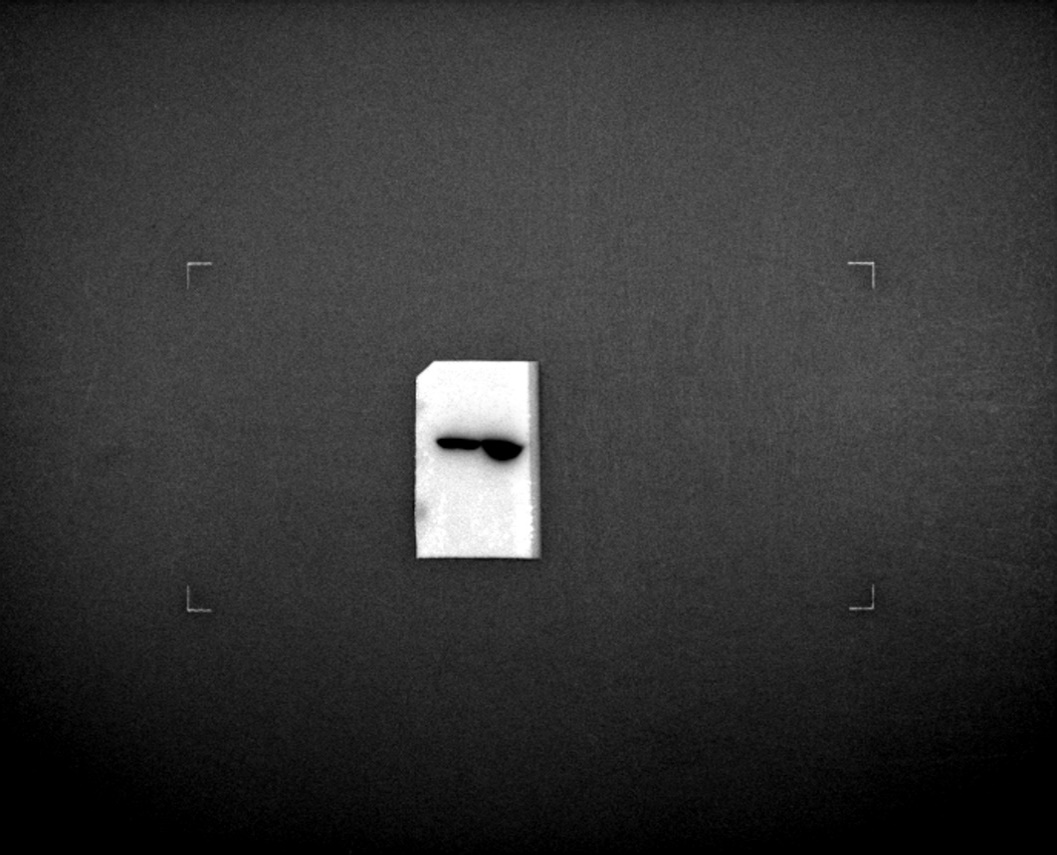

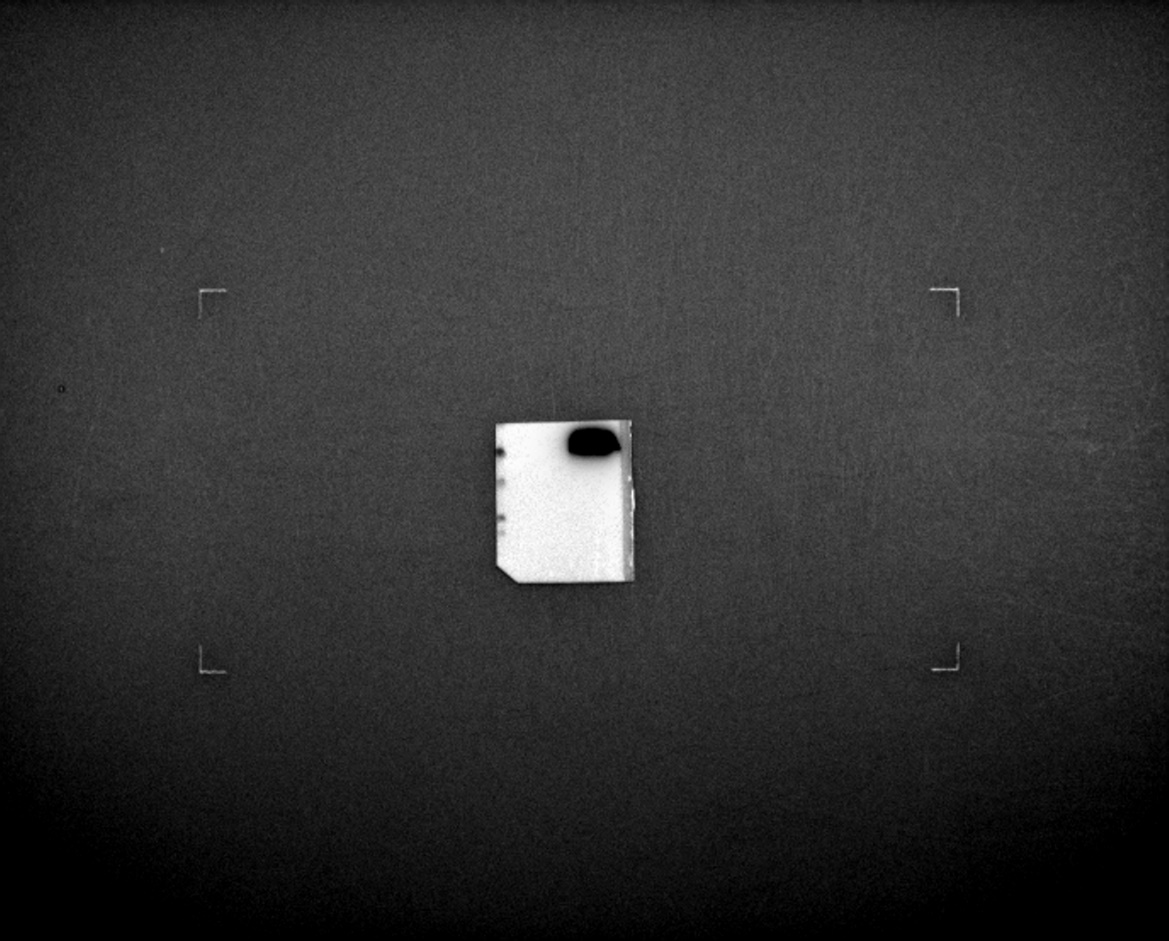

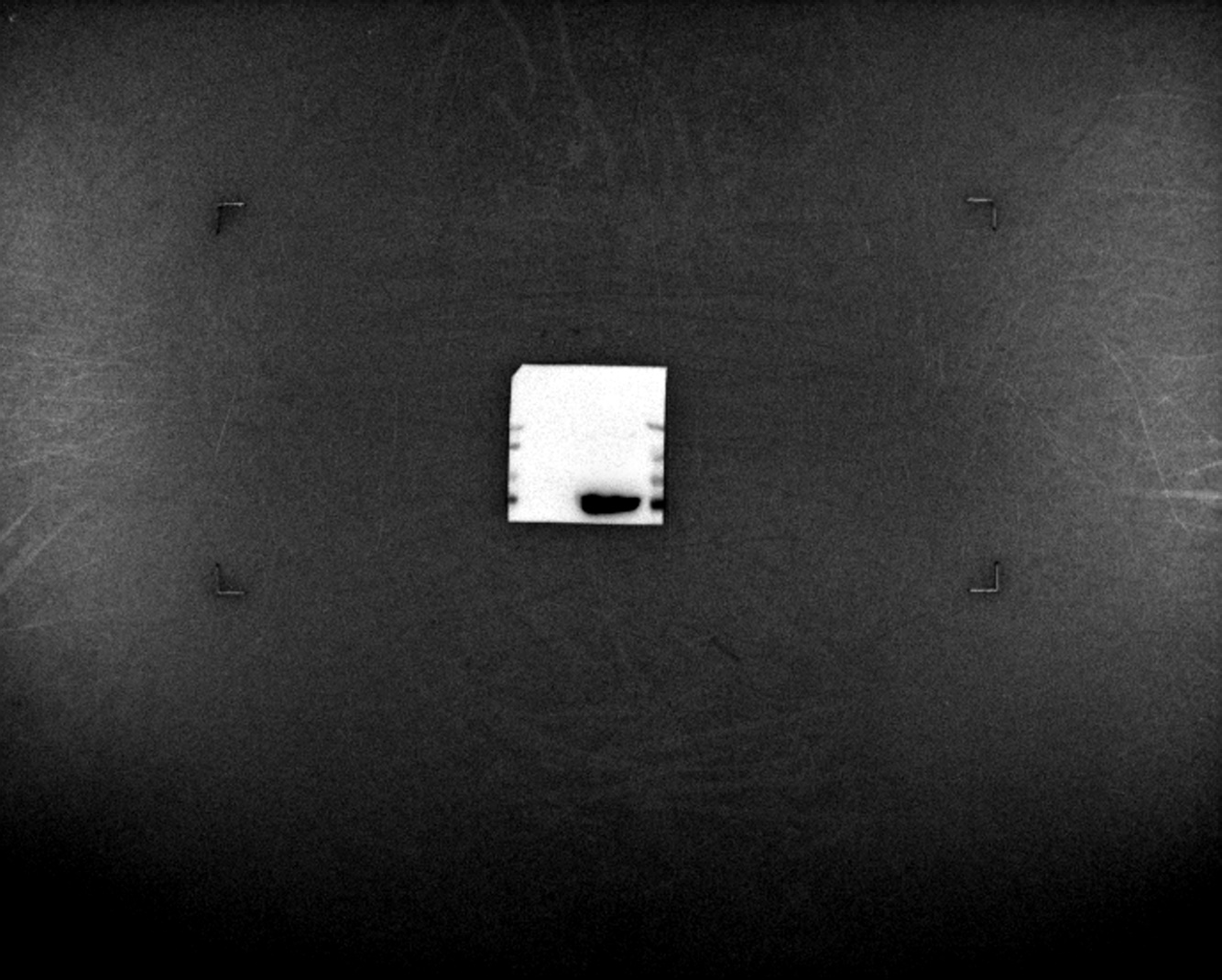

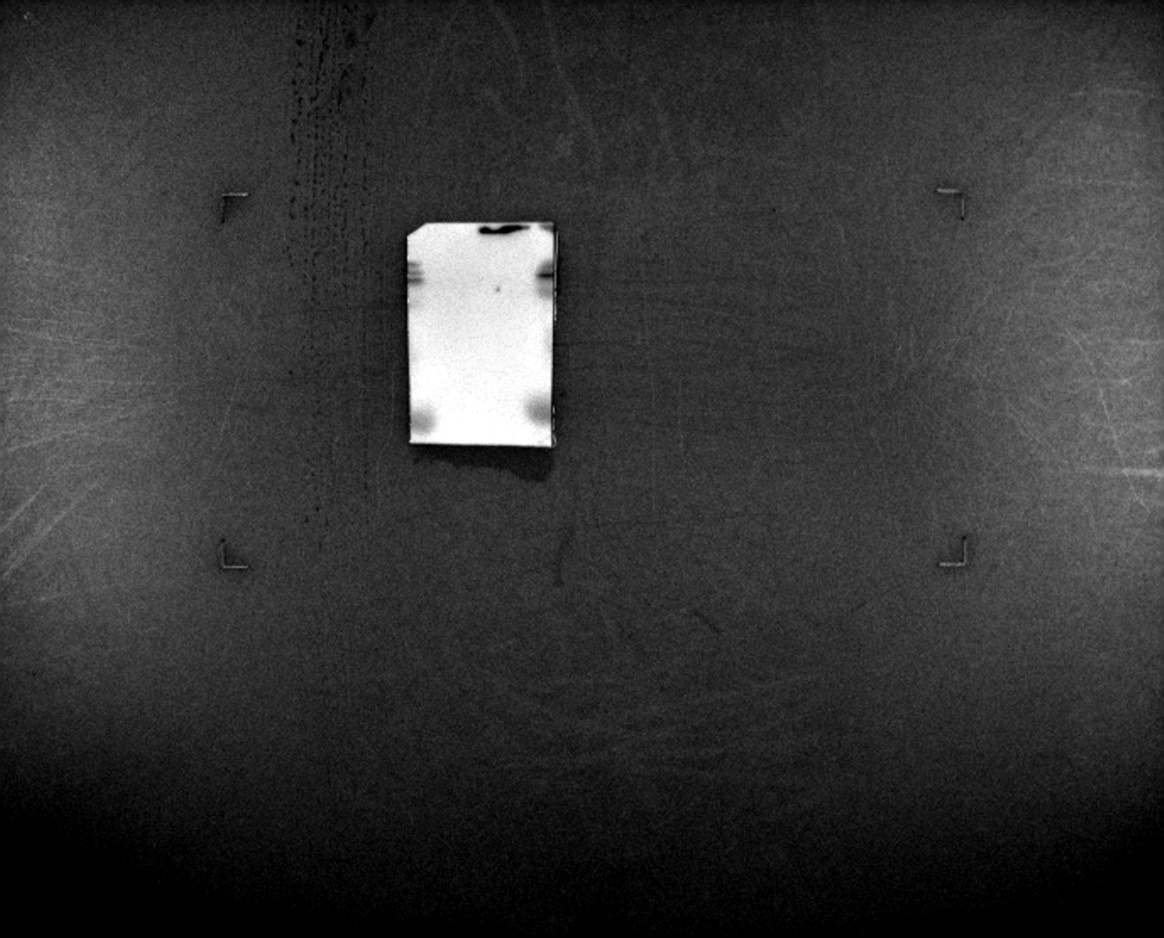

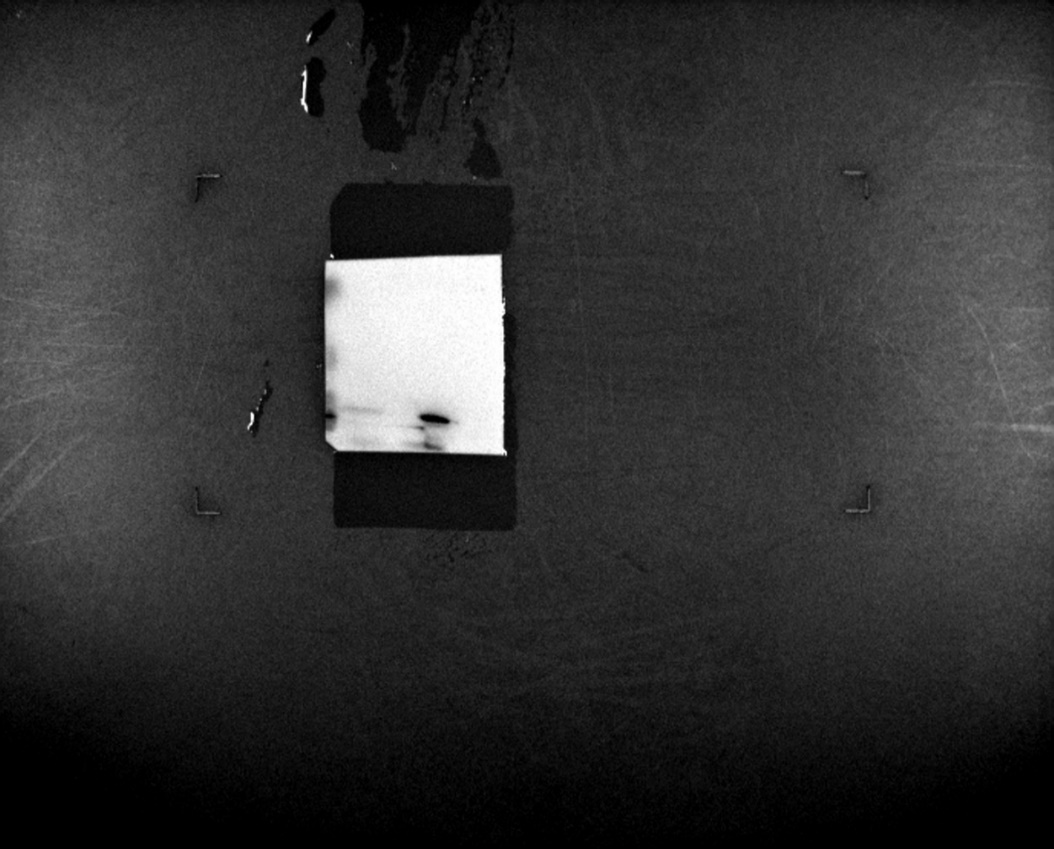


G:(Actin, RRM1, Flag-RRM1, RAD51AP1)


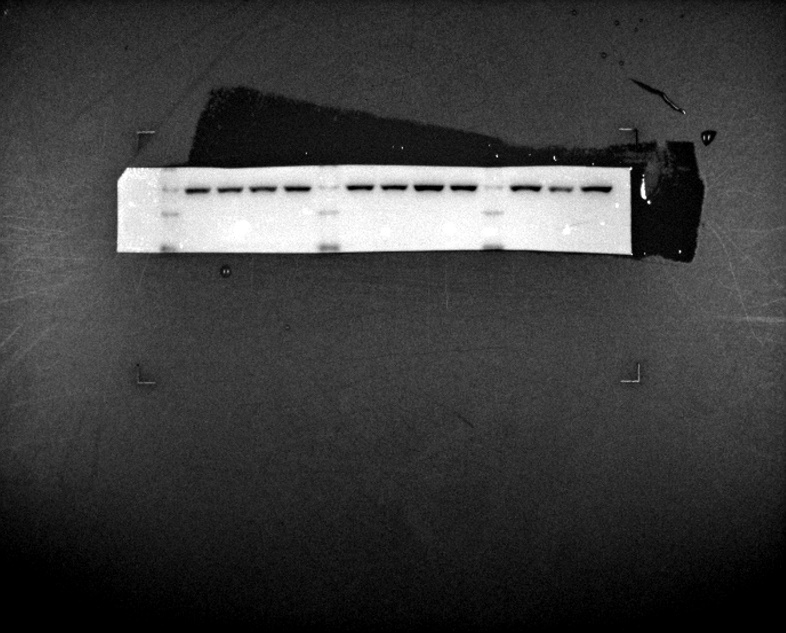

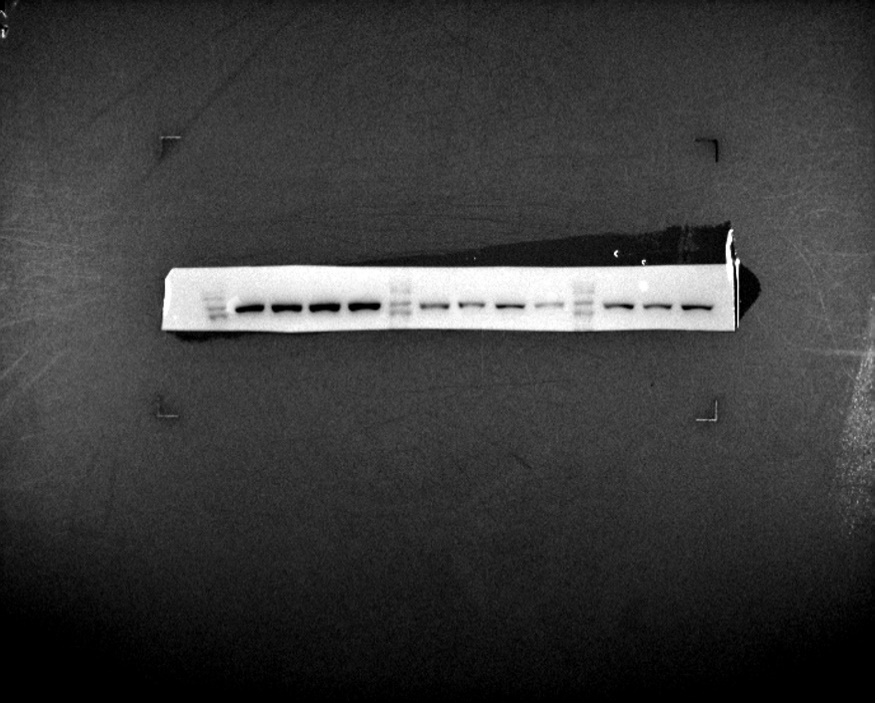


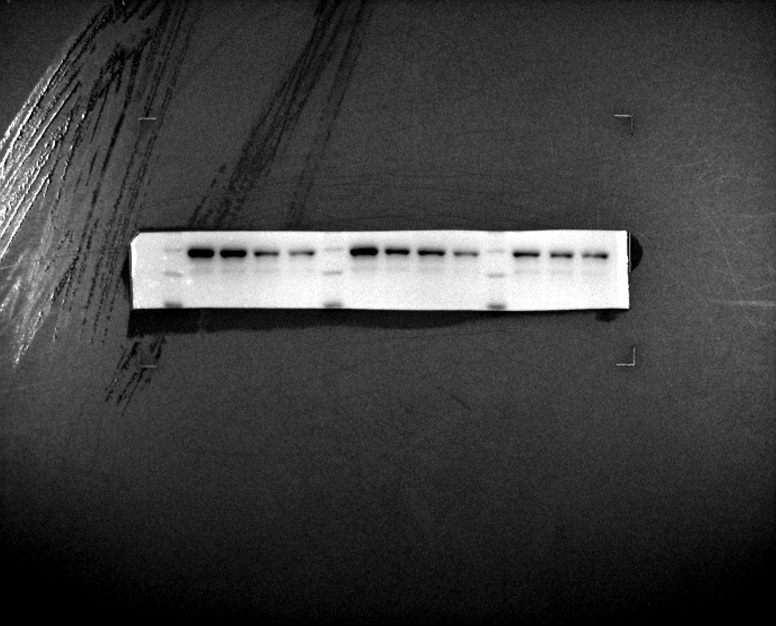


**Fig. SM3**

A:(Actin, RAD51AP1）


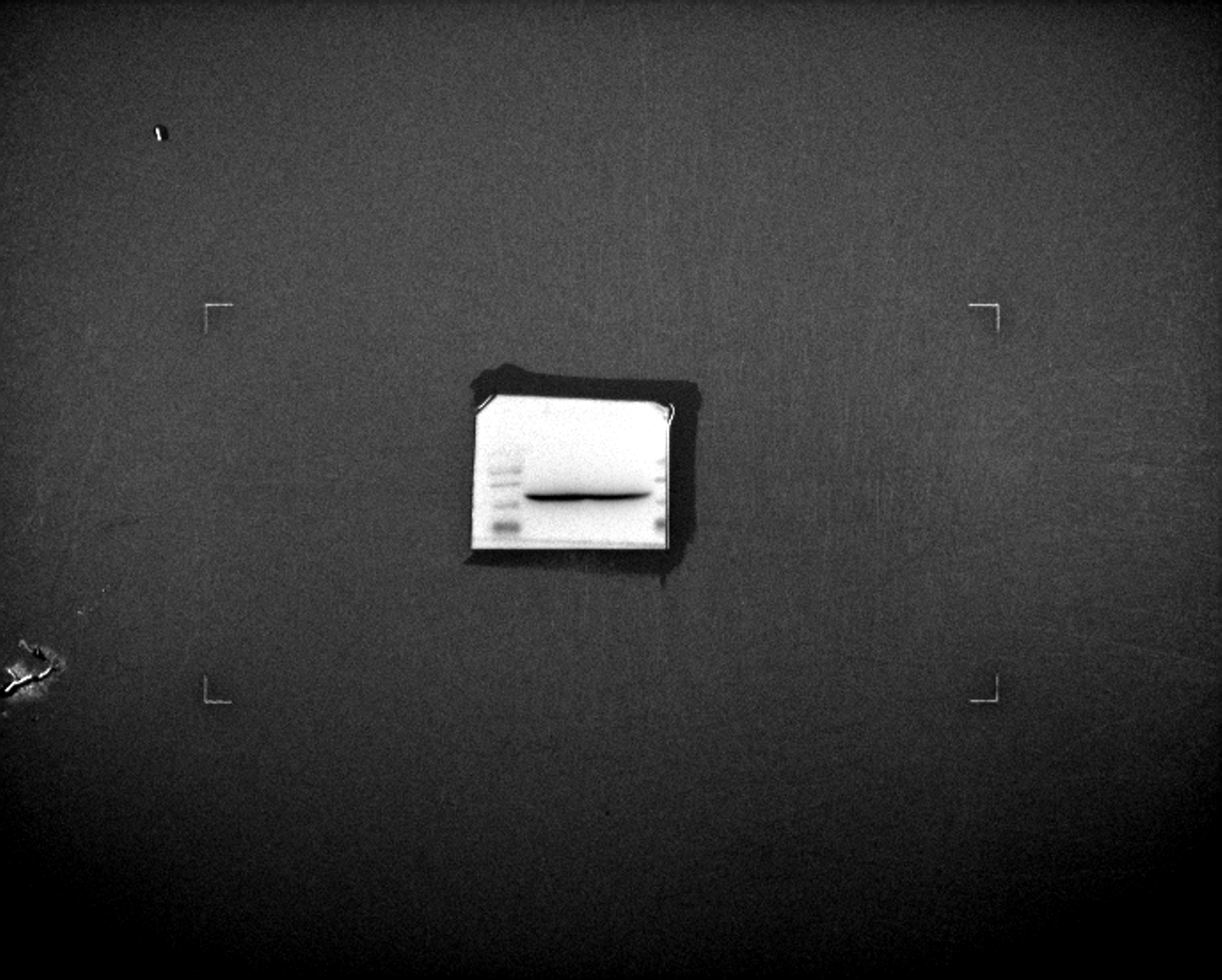

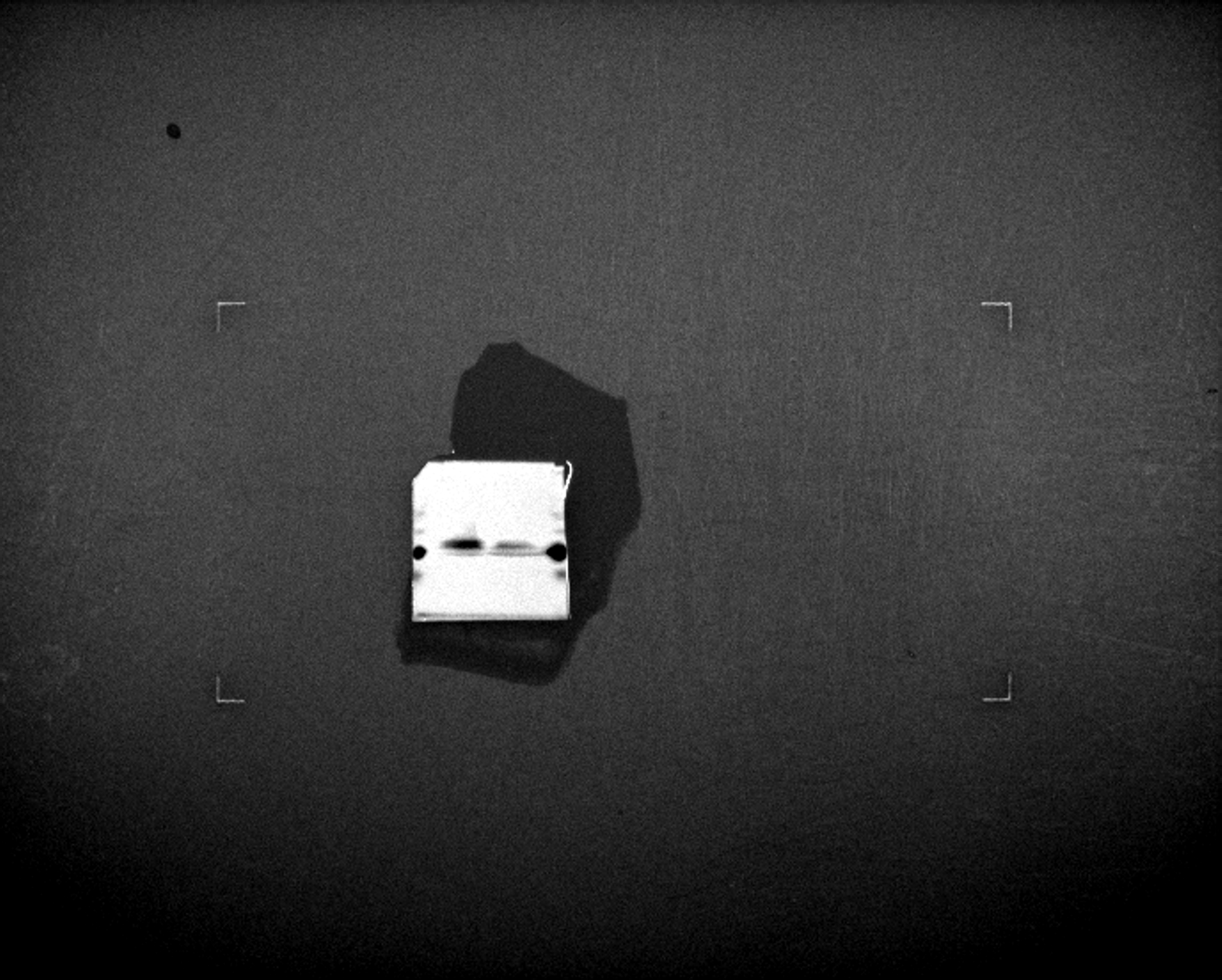


B:(Actin, E2F1, RAD51AP1)


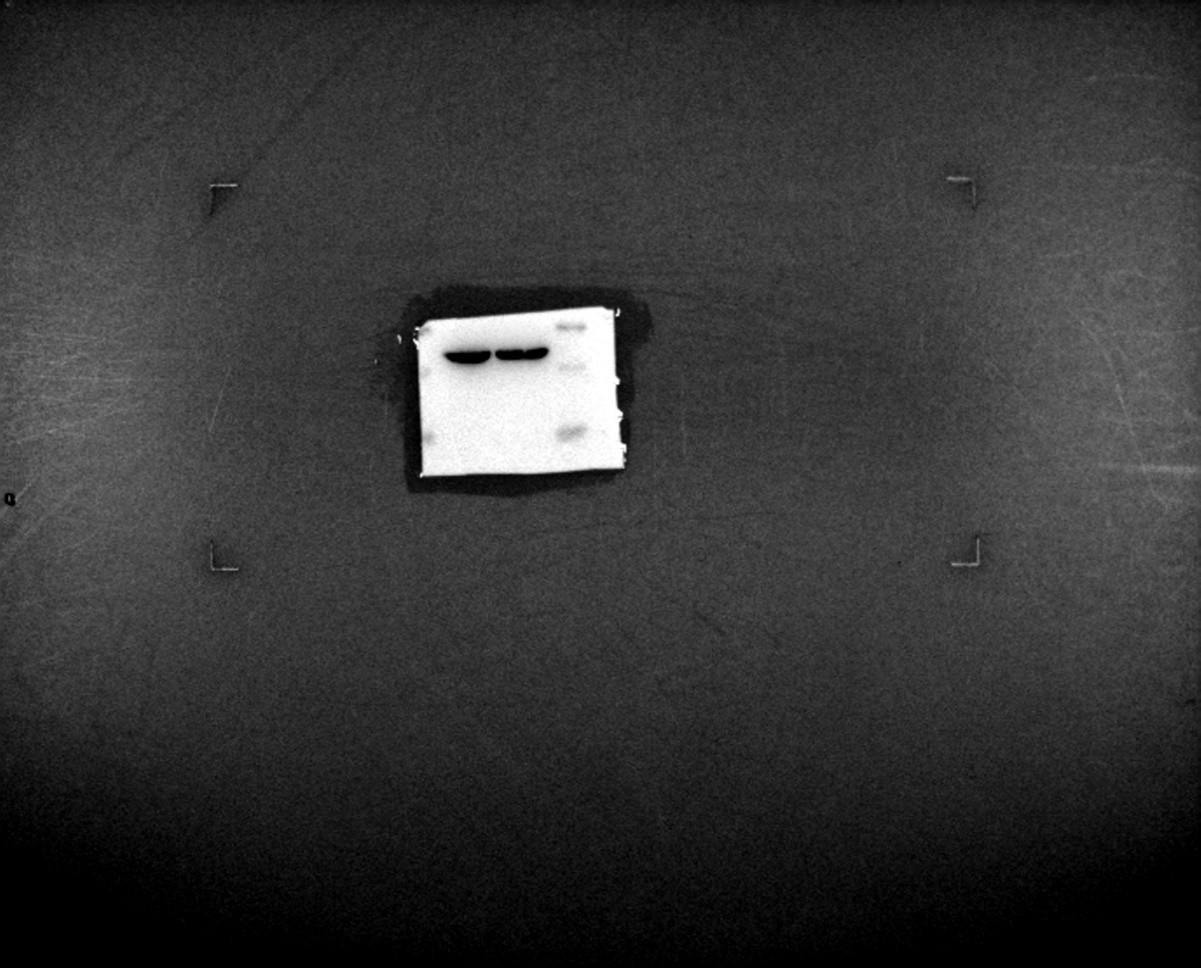

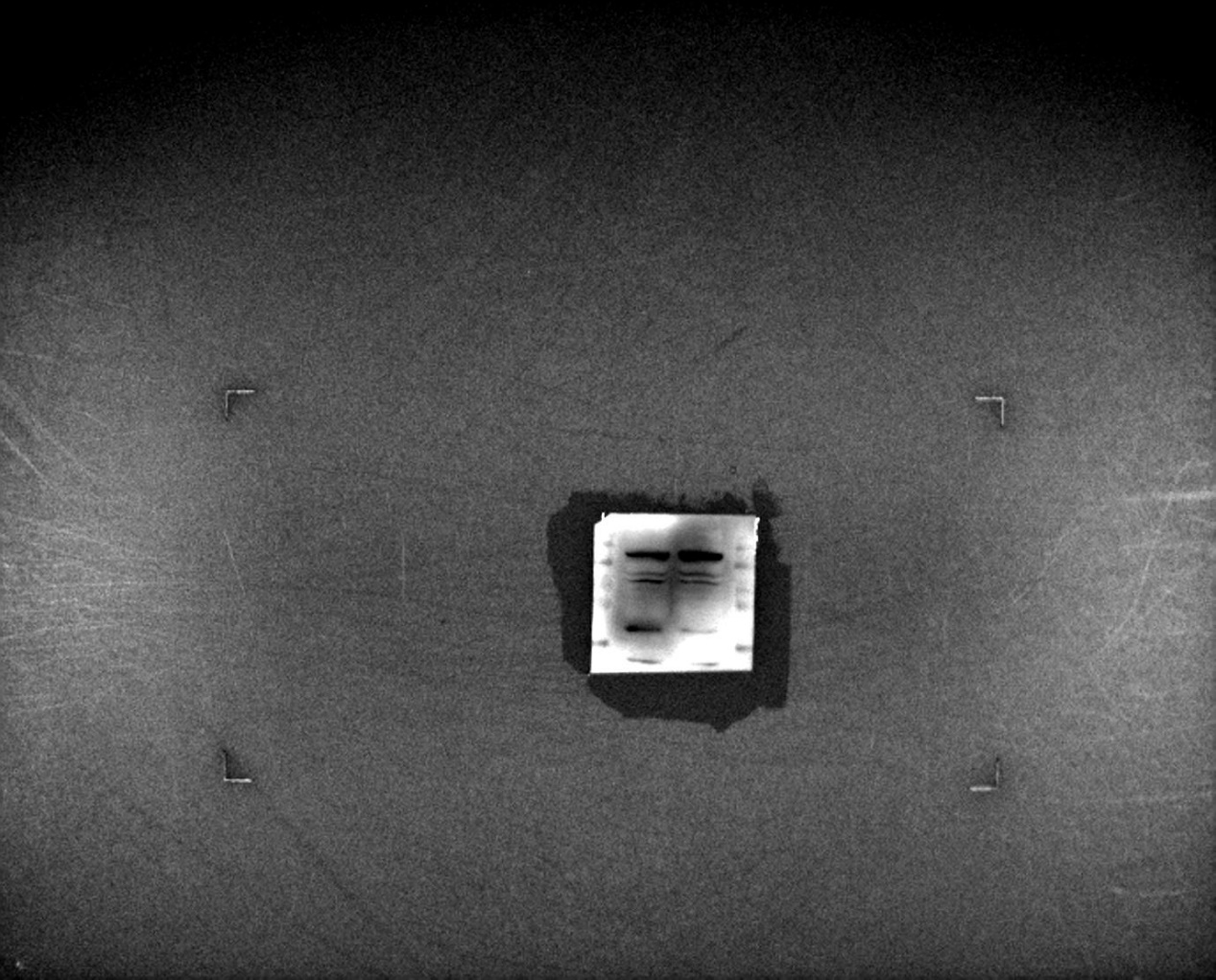

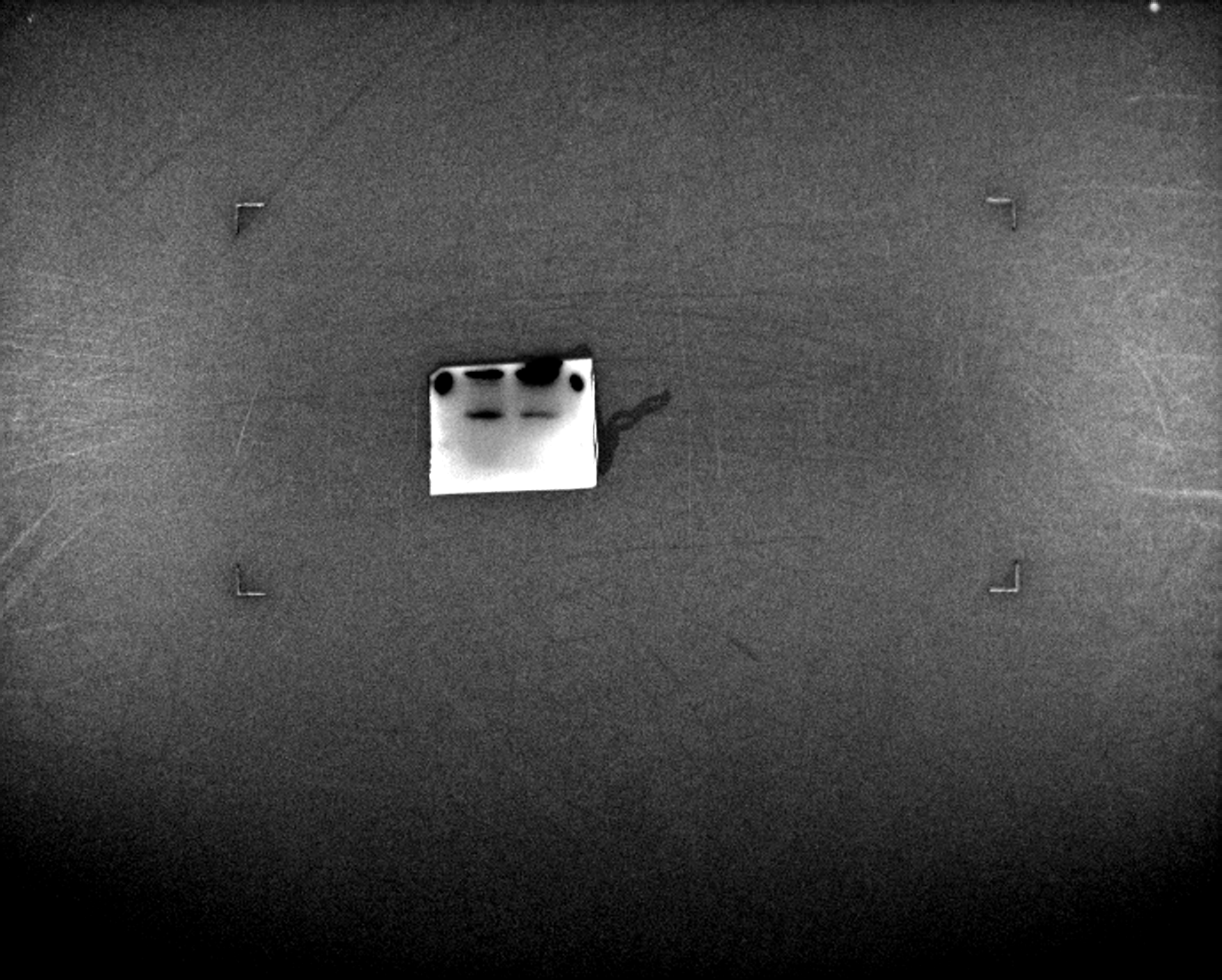


C:( E2F1, Actin, RRM1)


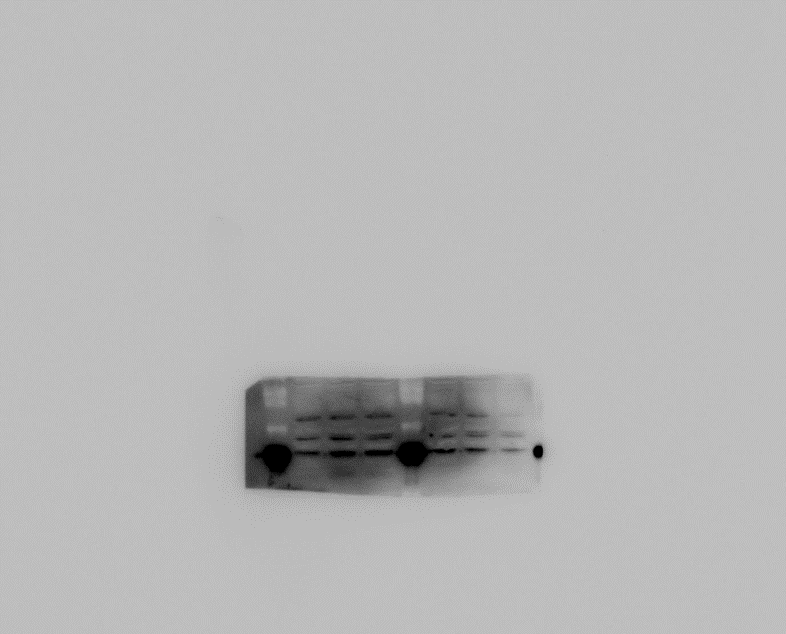

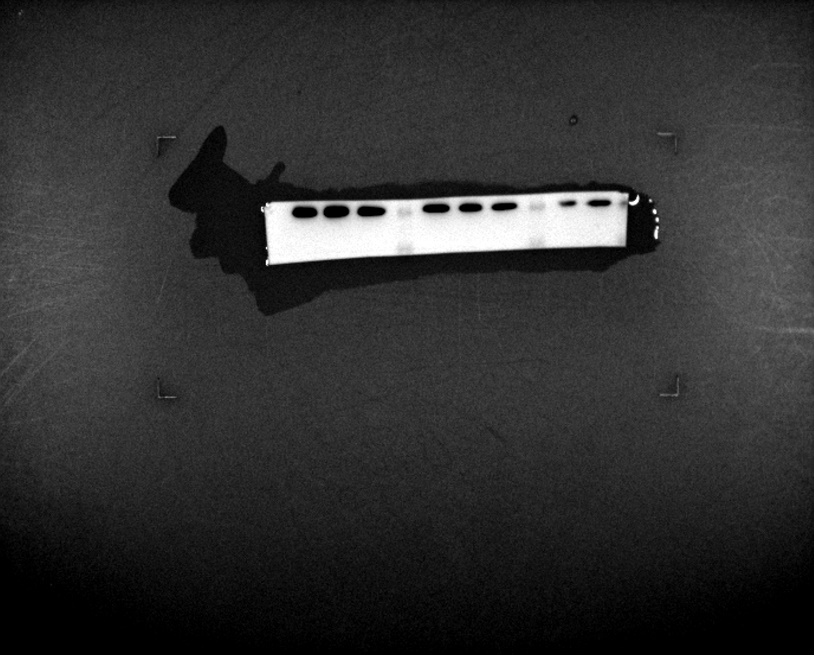

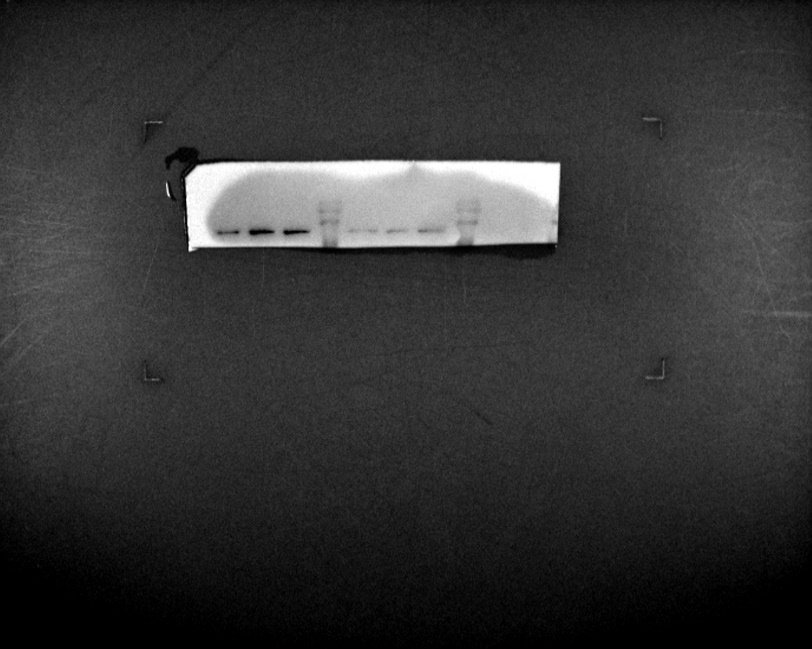


D:( Actin, E2F1)


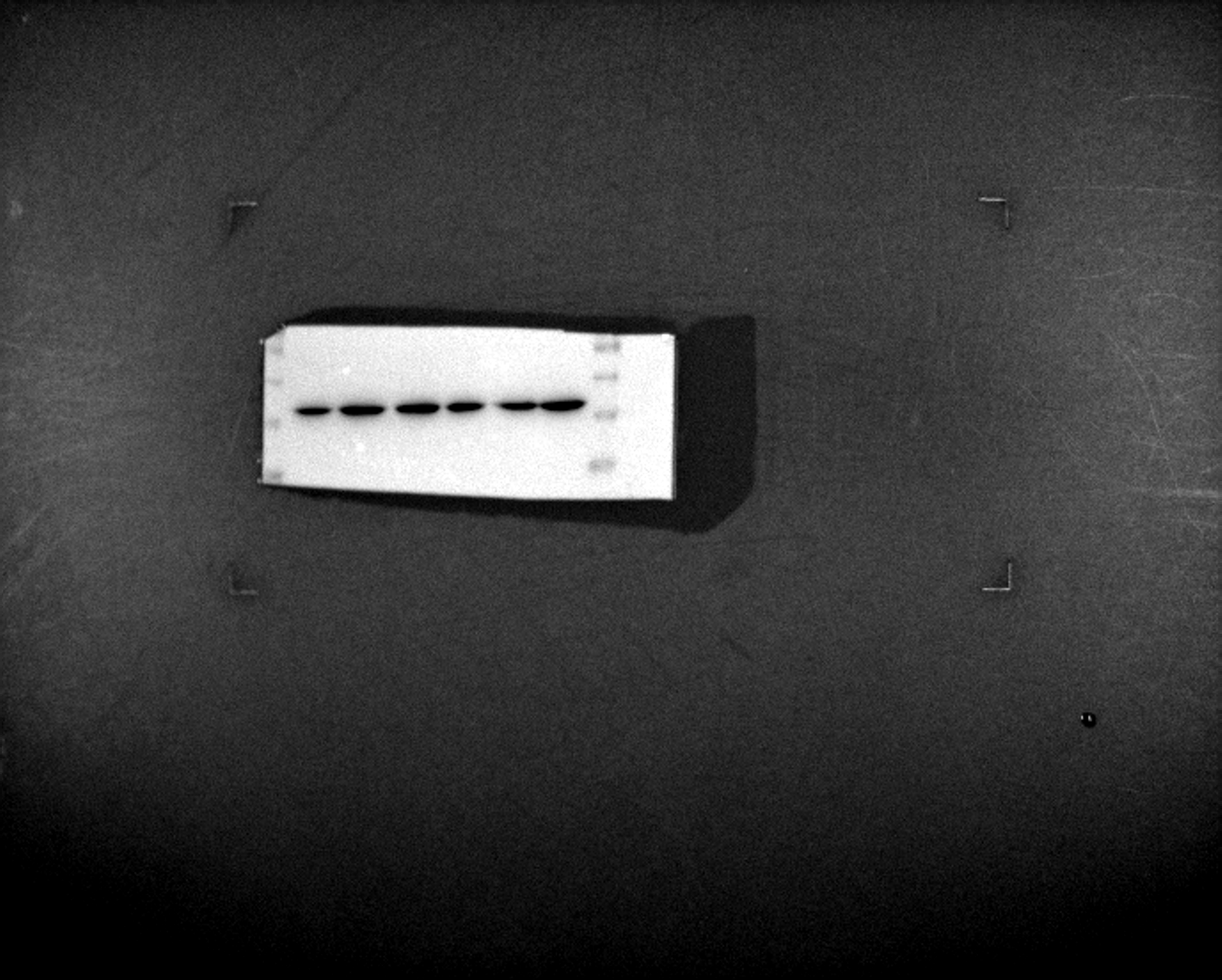

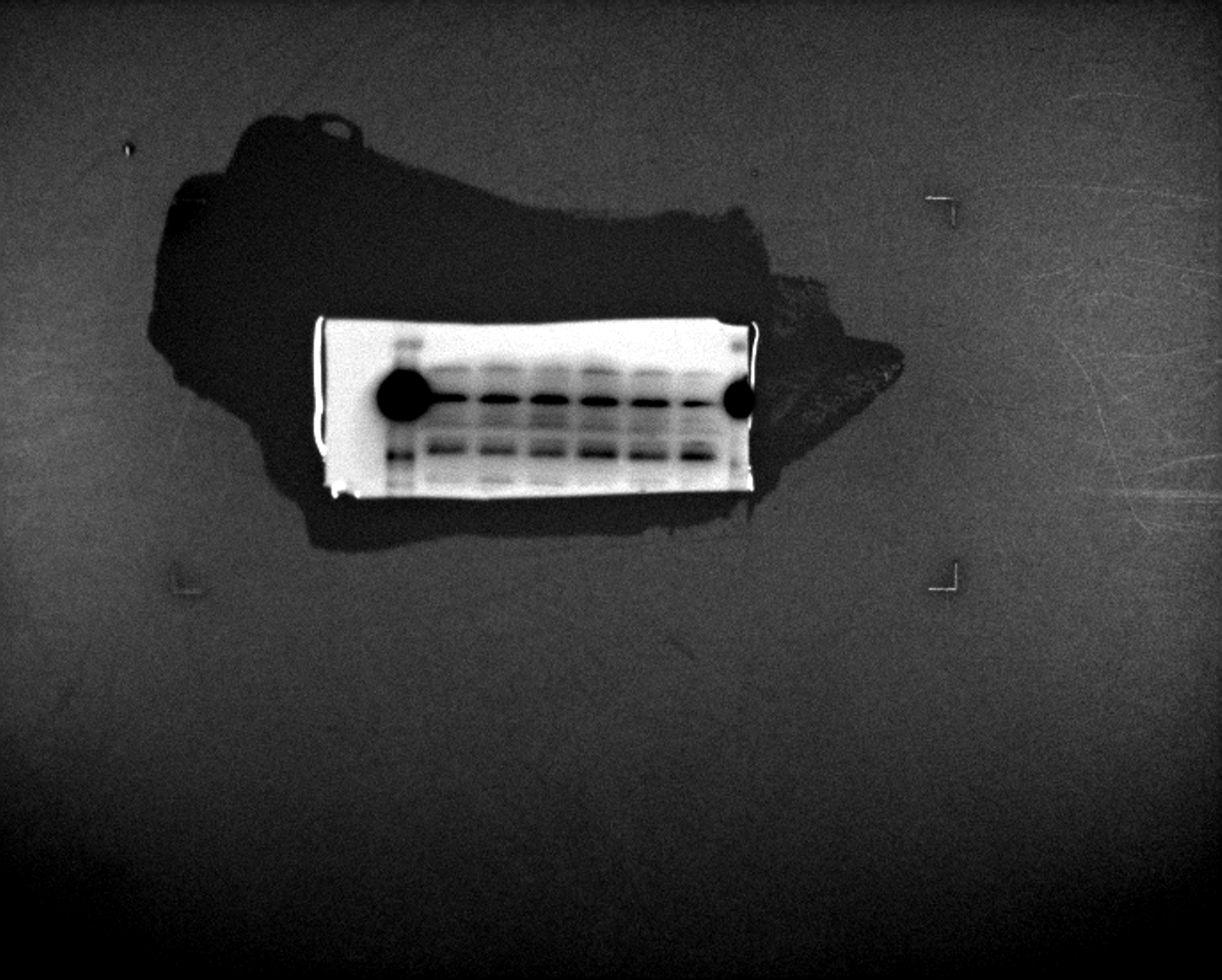


E:(Ub, E2F1)


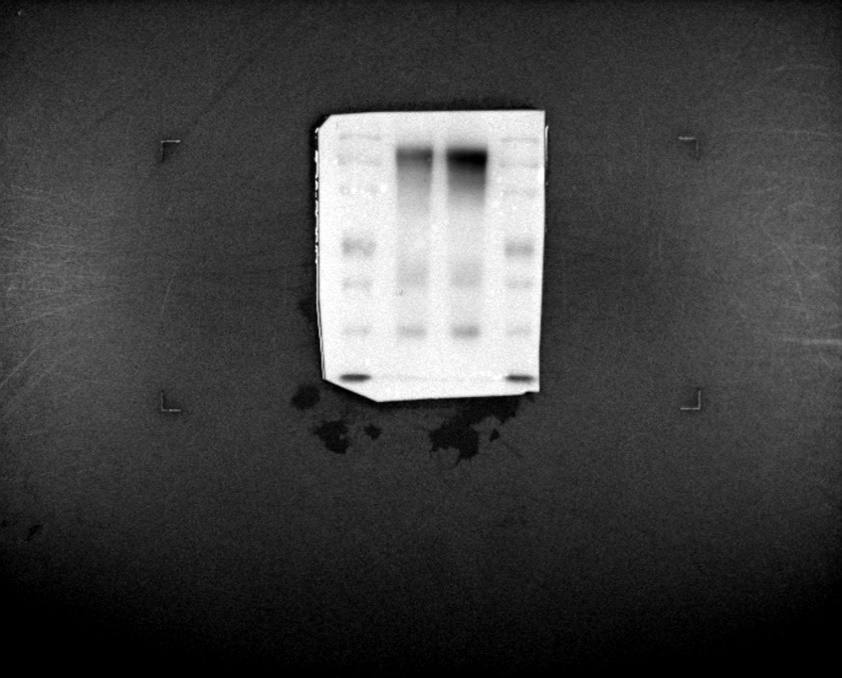

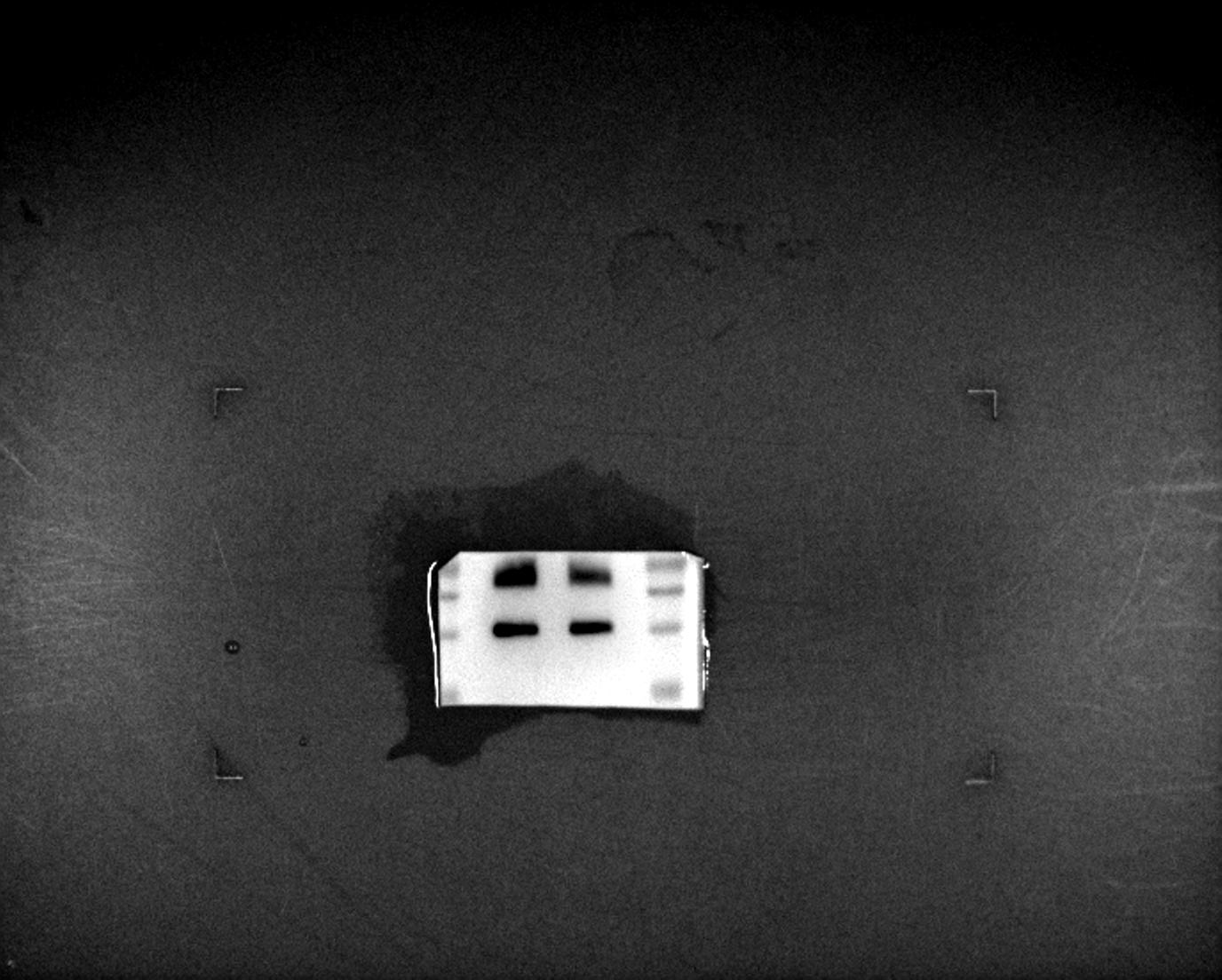


G:(RRM1(anti-RRM1), RRM1(anti-E2F1), E2F1(anti-RRM1), E2F1(anti-E2F1))


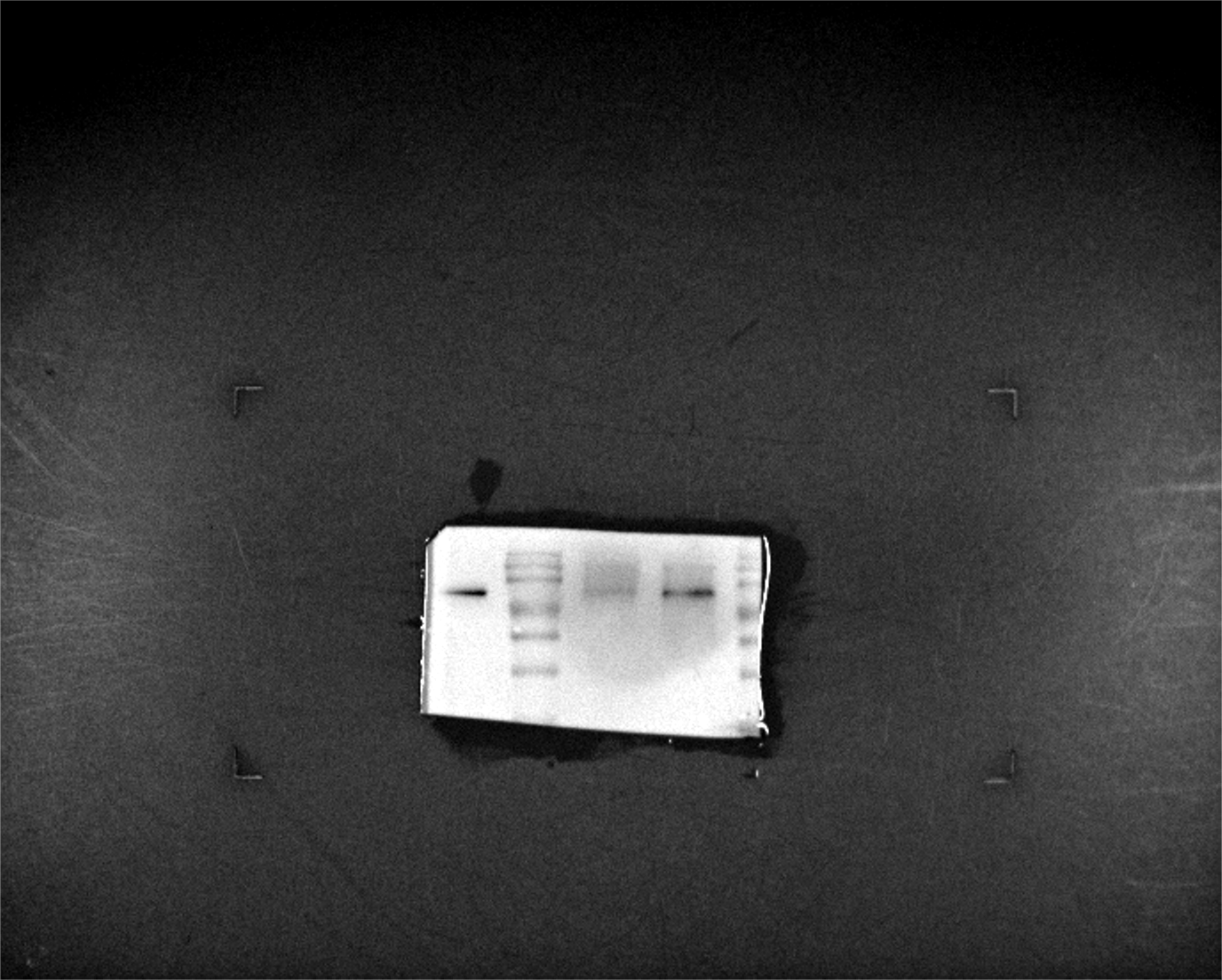

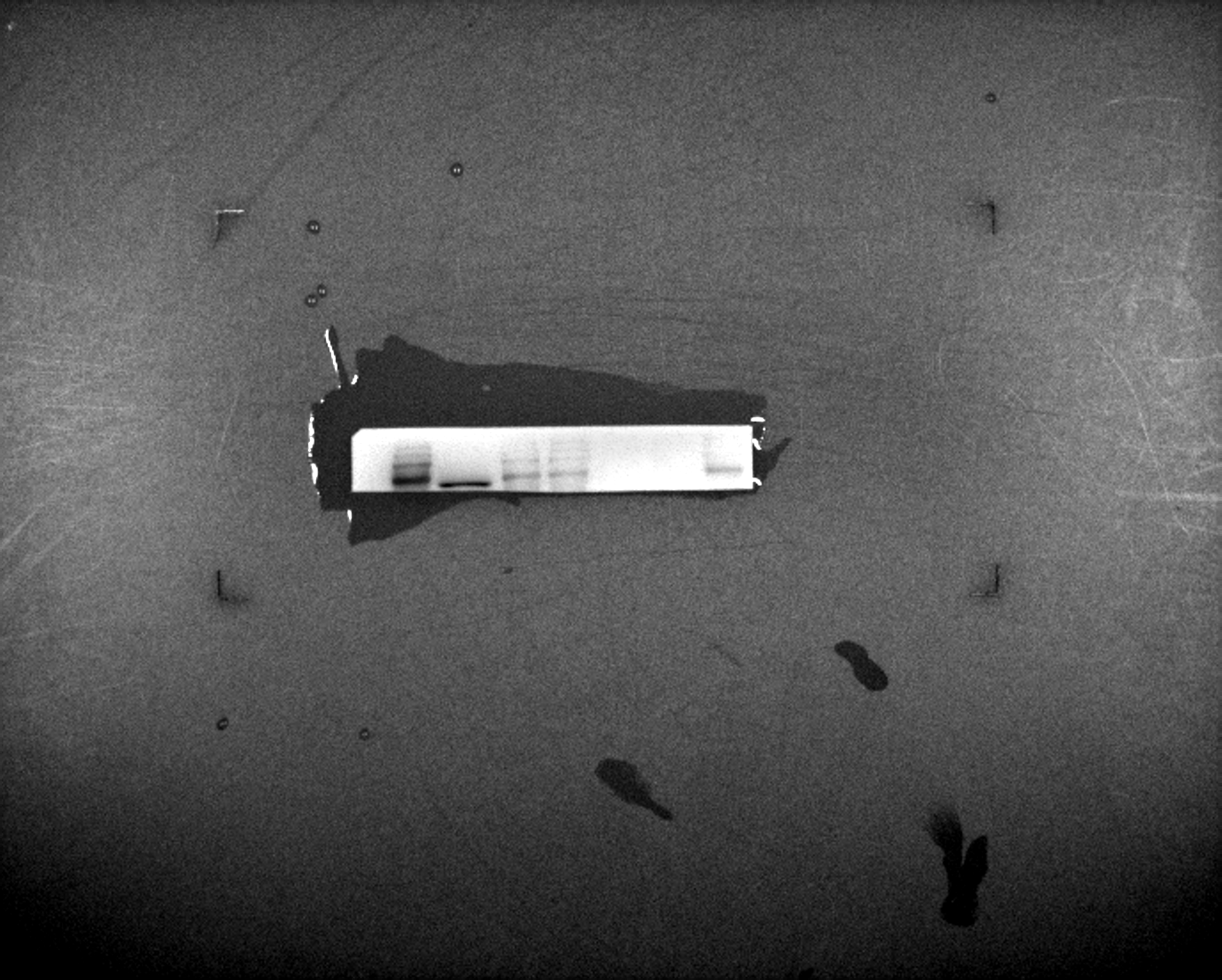


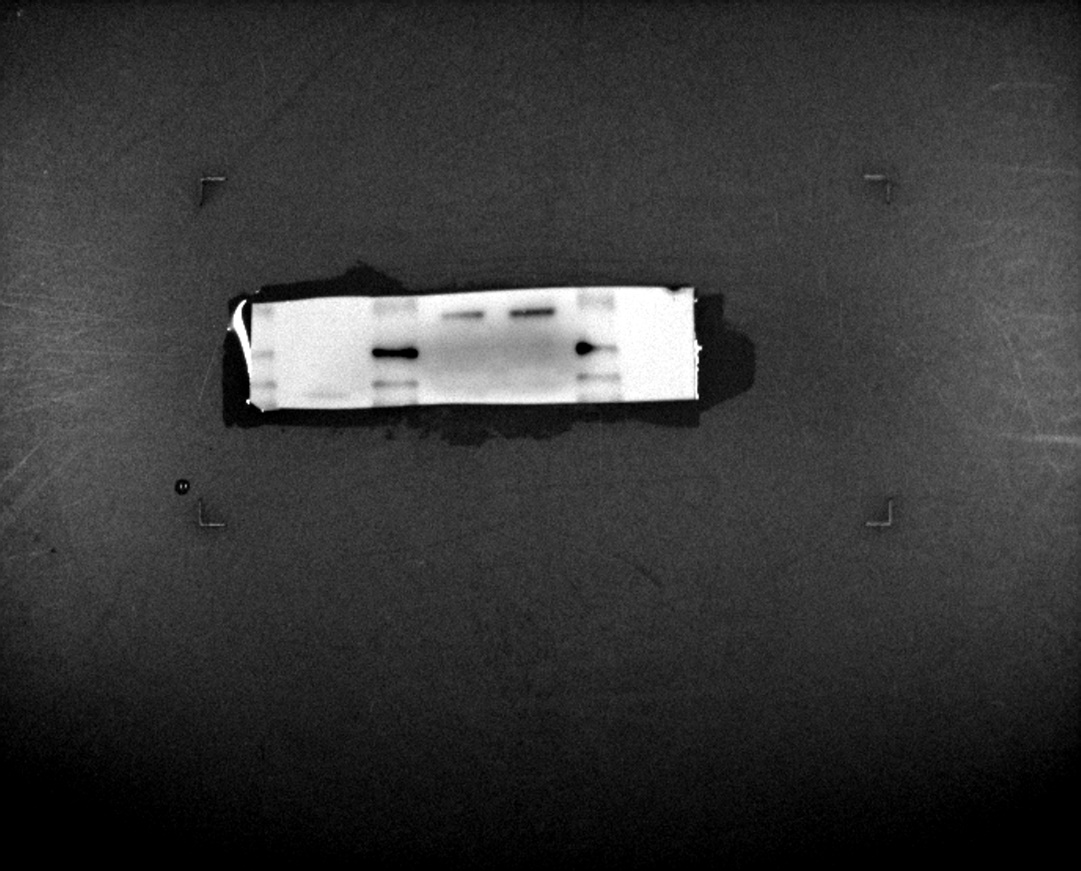

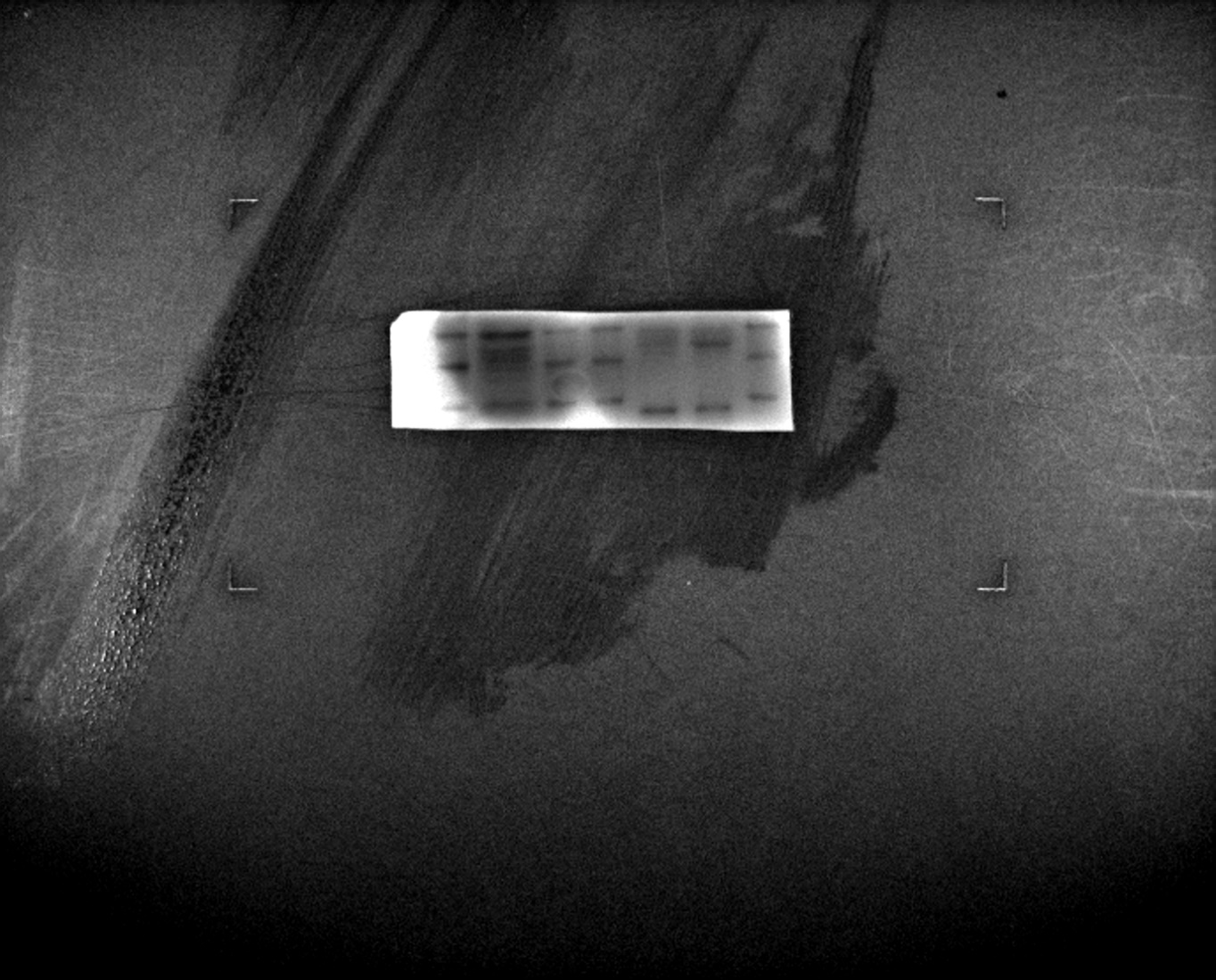


**Fig. SM4**

A:(Ub, IP-E2F1, E2F1, Actin, RRM1)


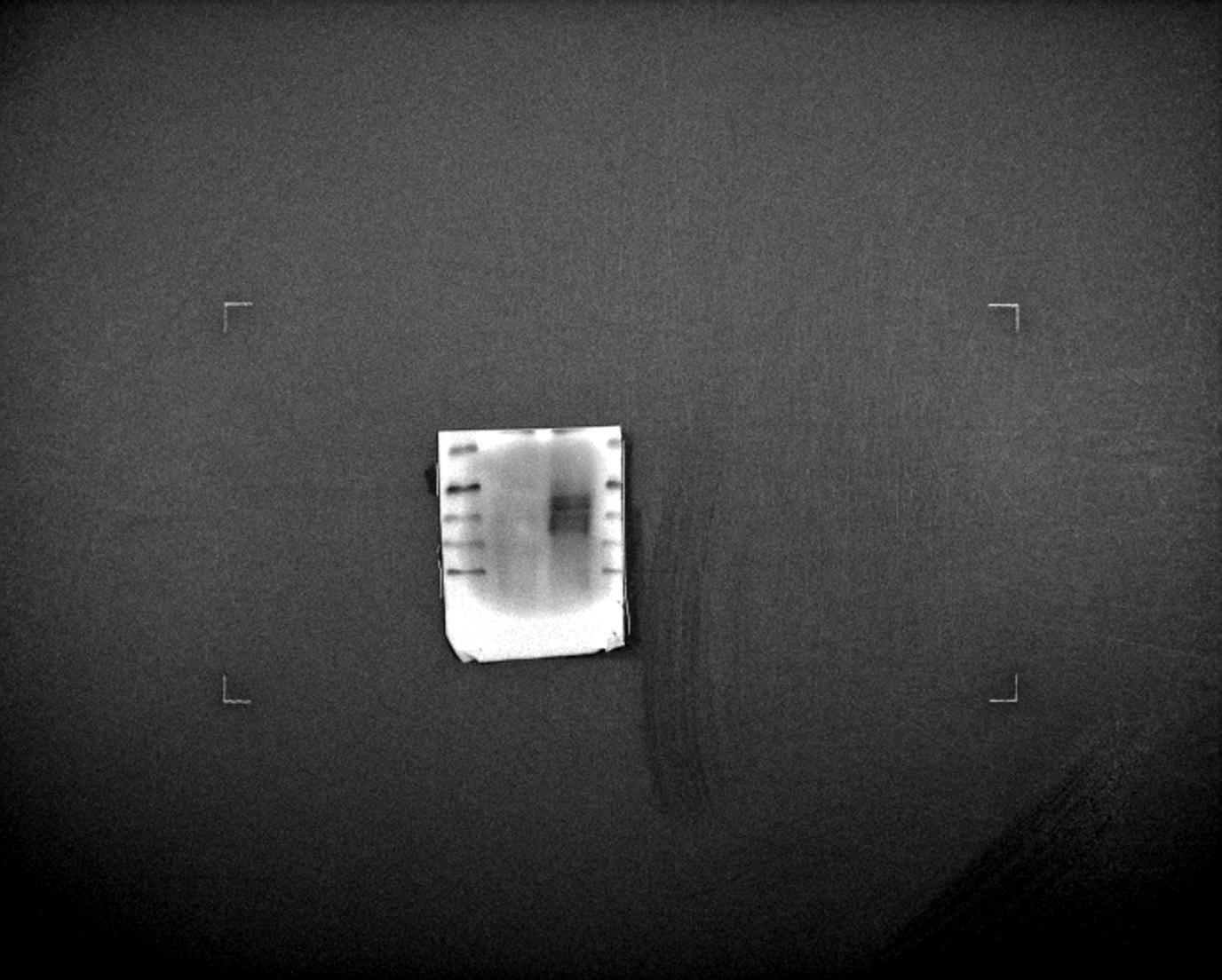

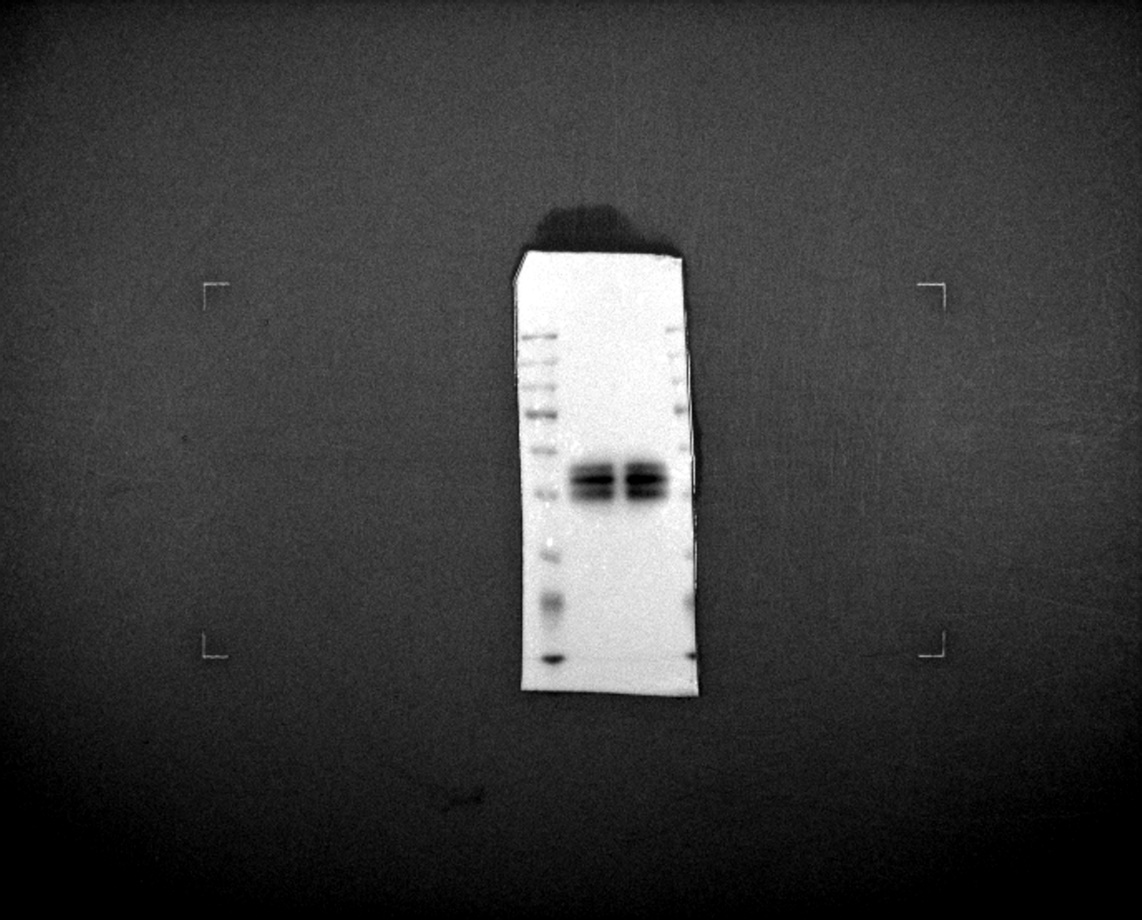


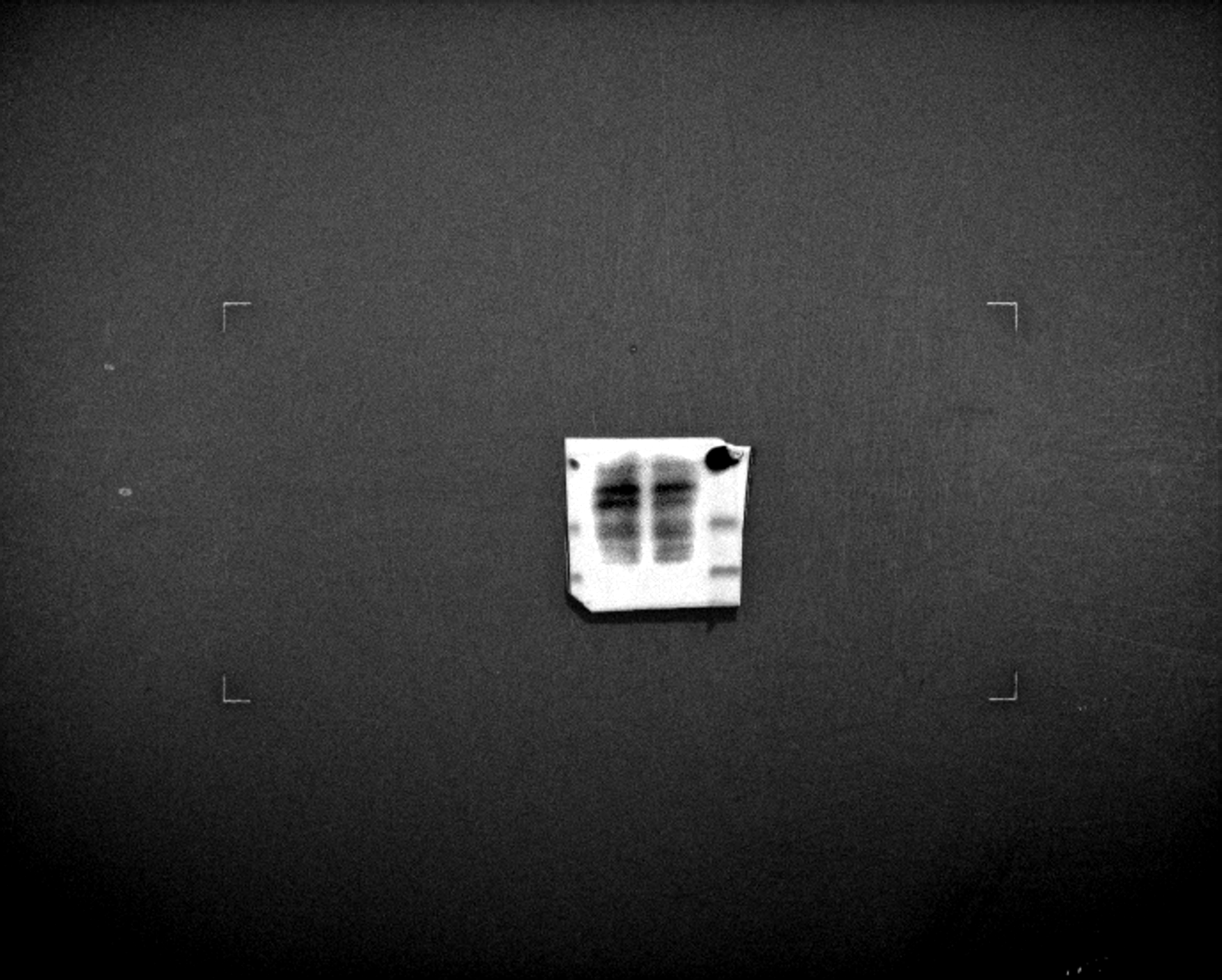

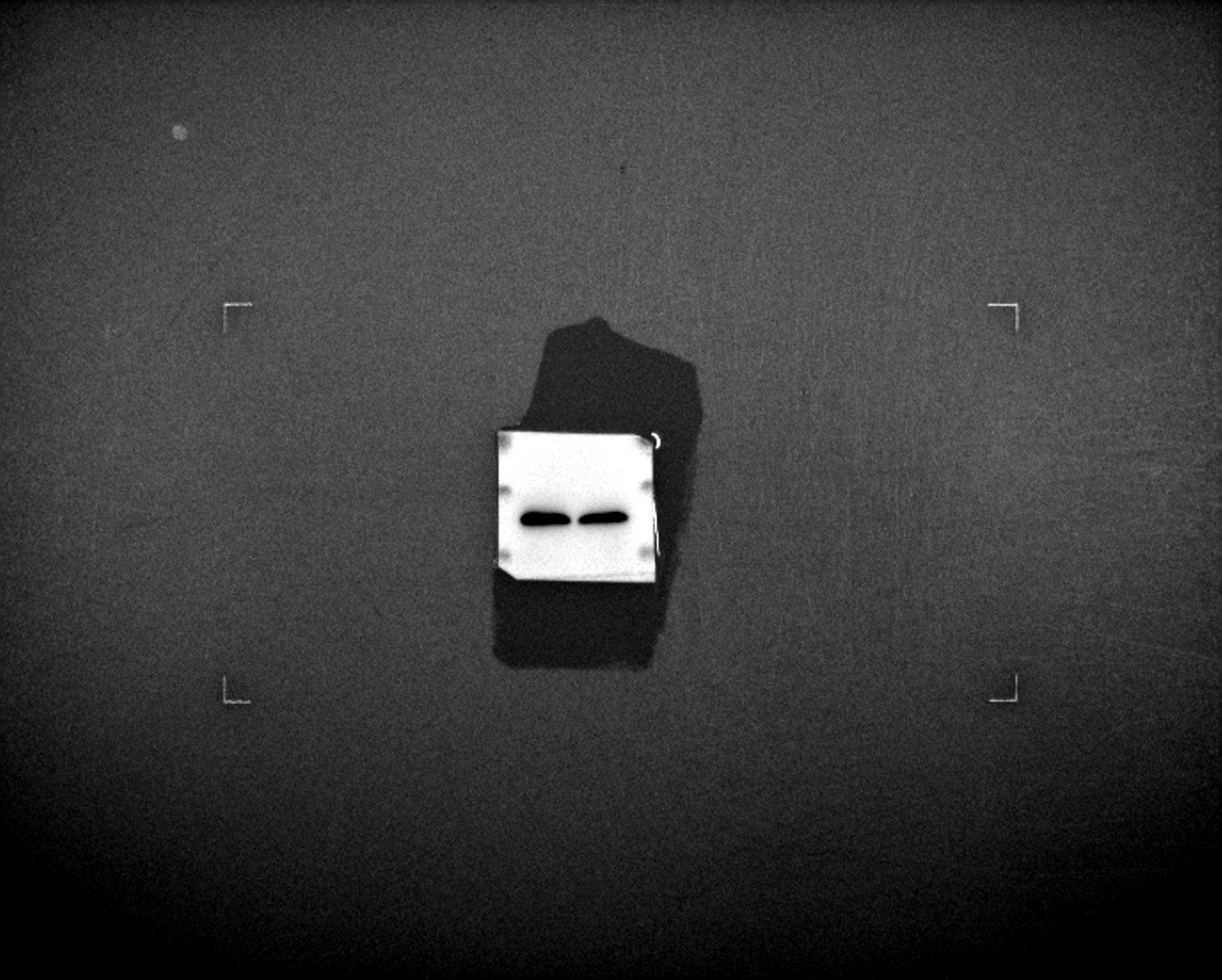

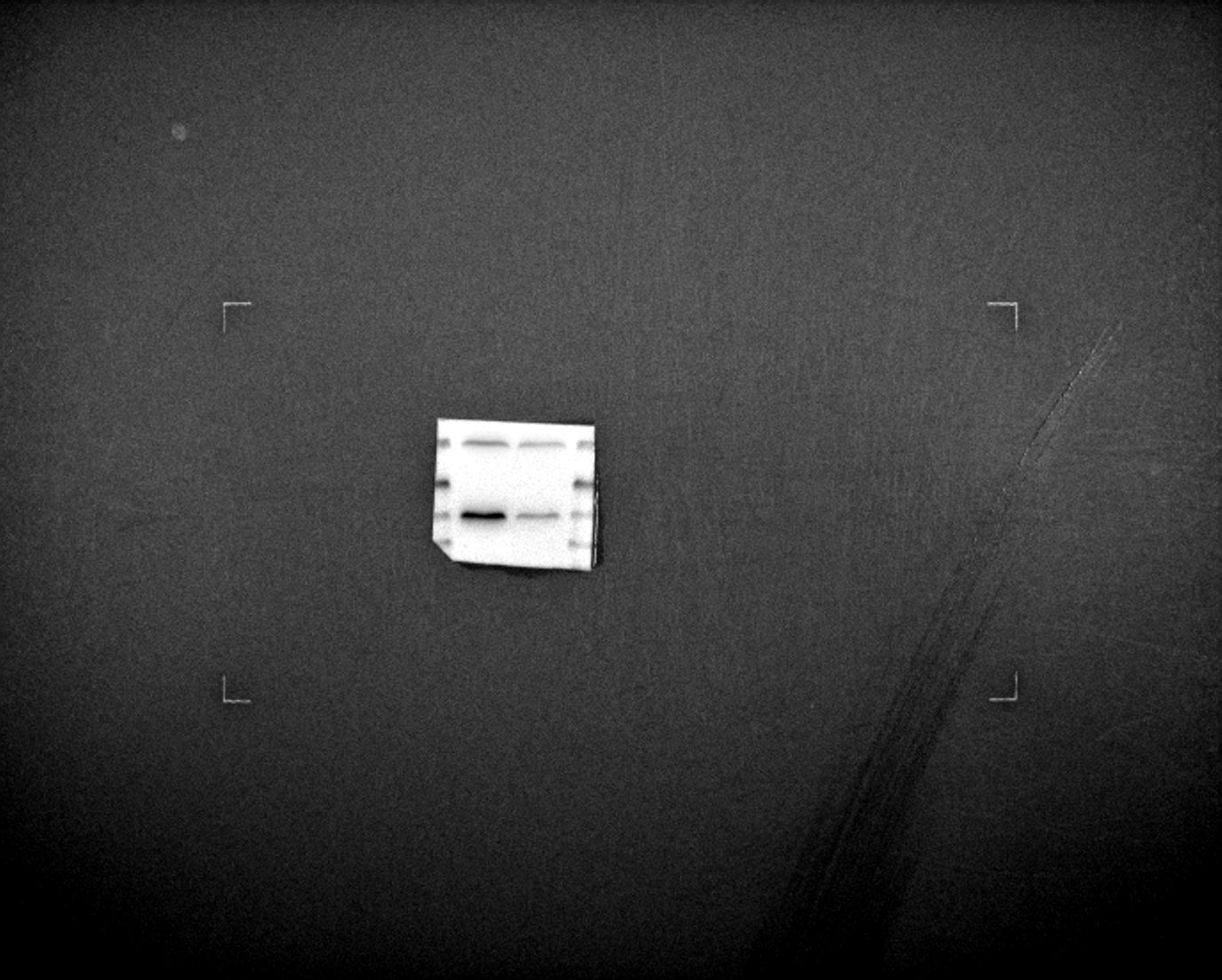


B:( USP11(anti-USP11), USP11(anti-RRM1), RRM1(anti-USP11), RRM1(anti-RRM1))


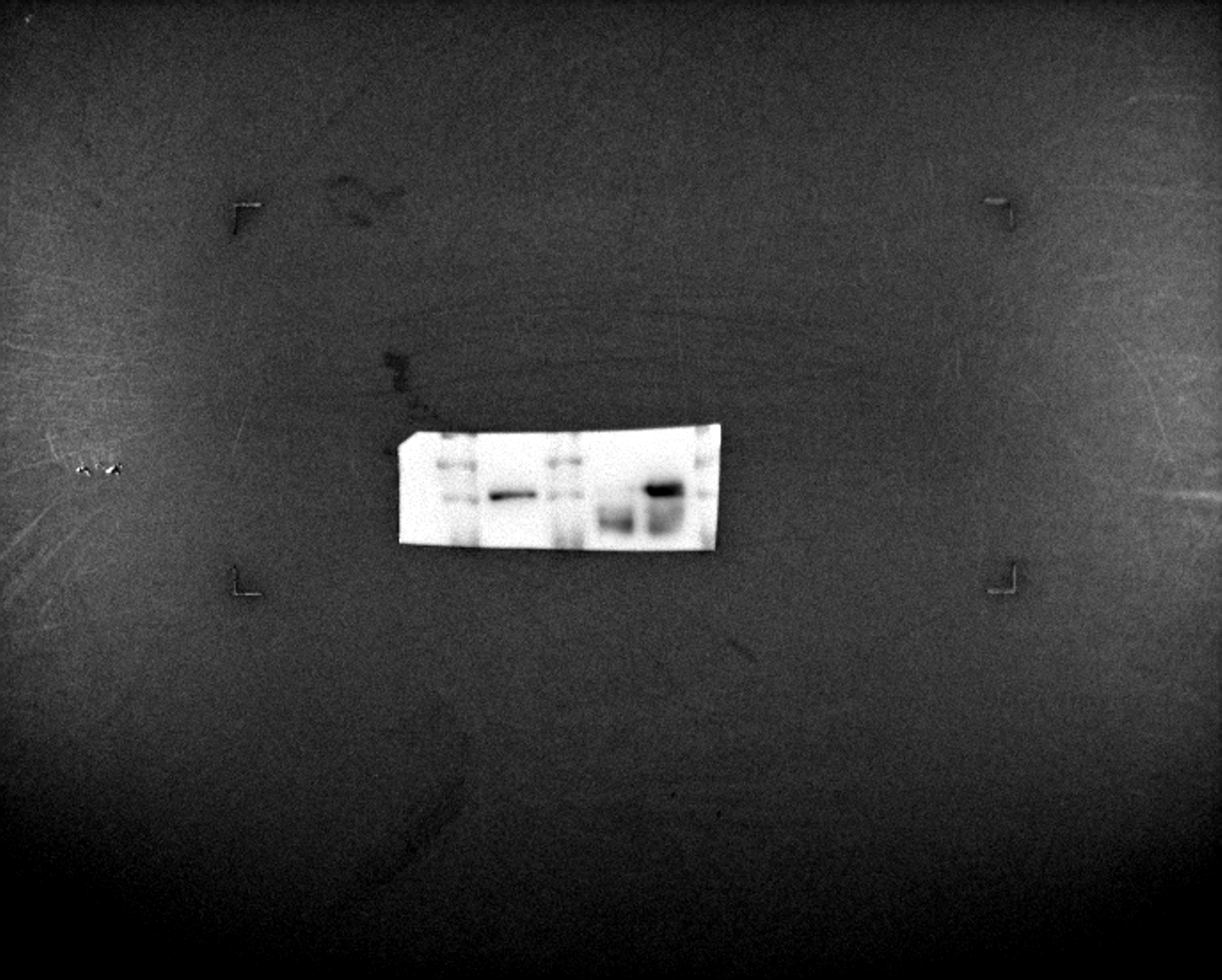

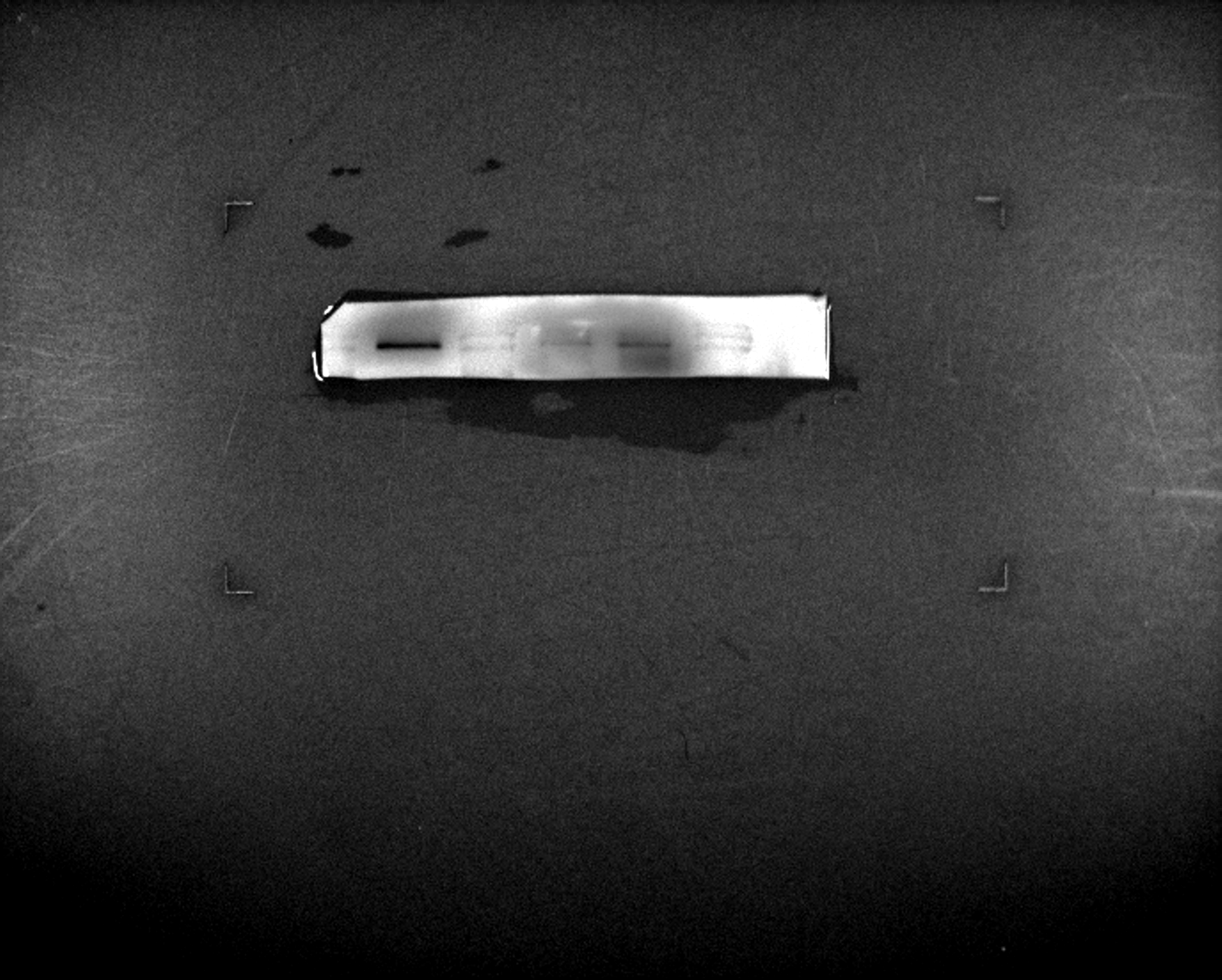


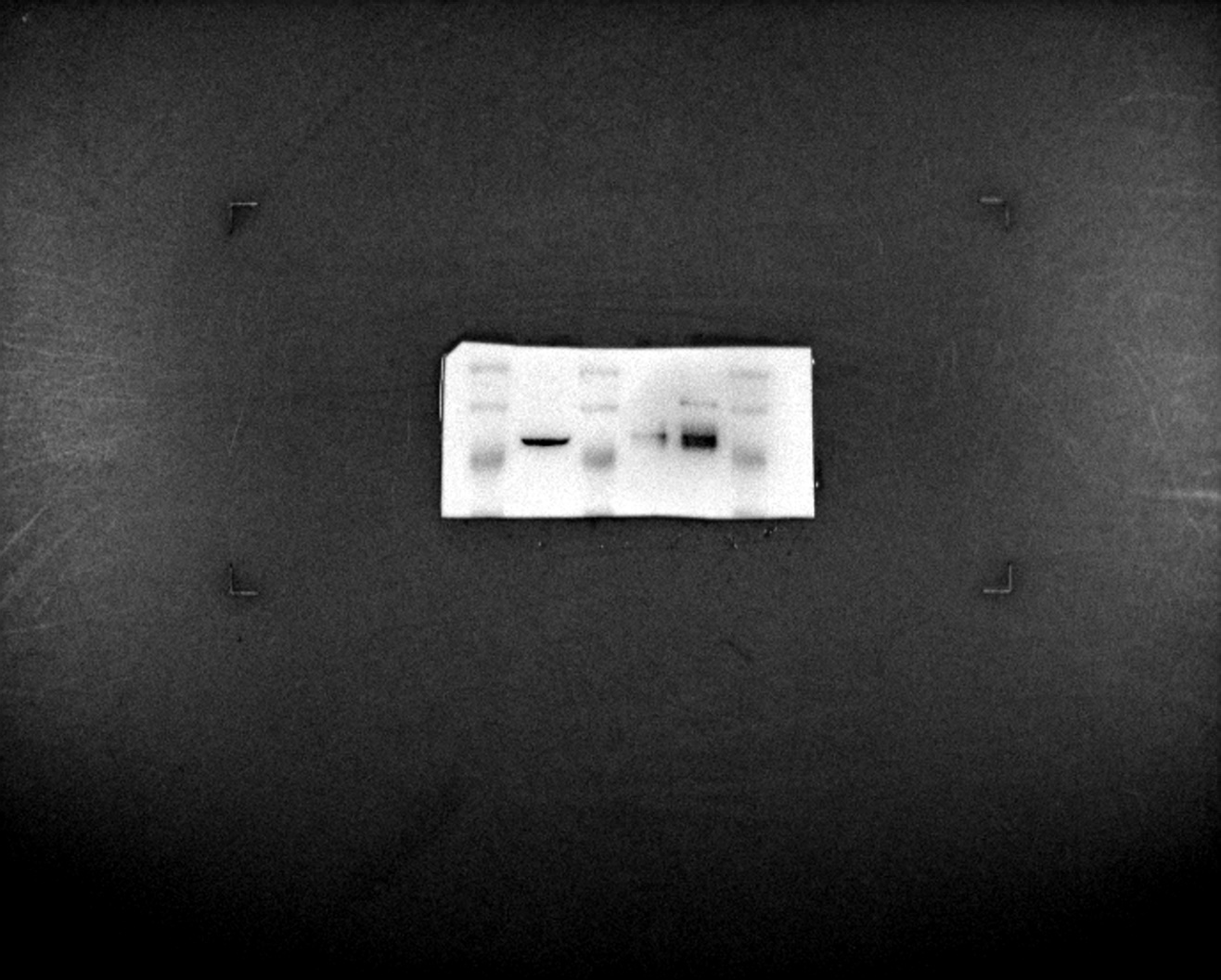

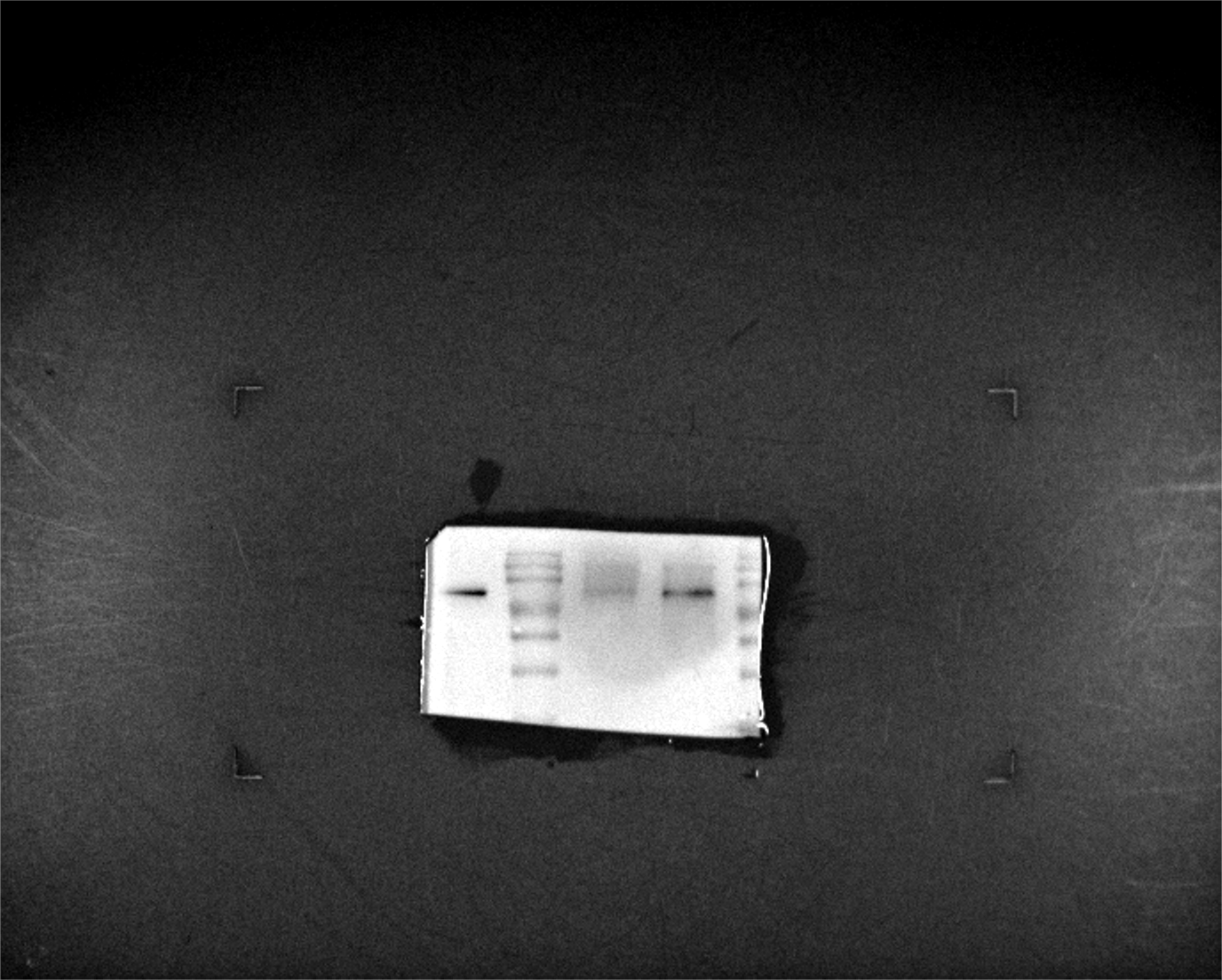


C:( IP-USP11, IP-RRM1, Input-RRM1, Actin)


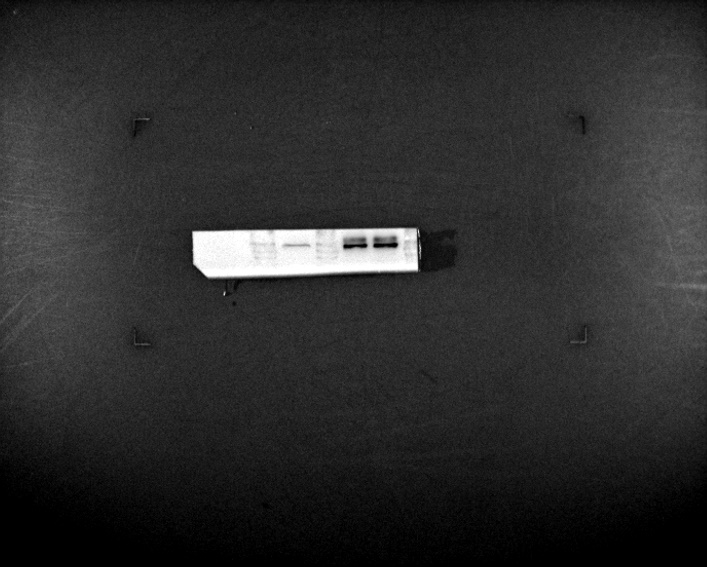

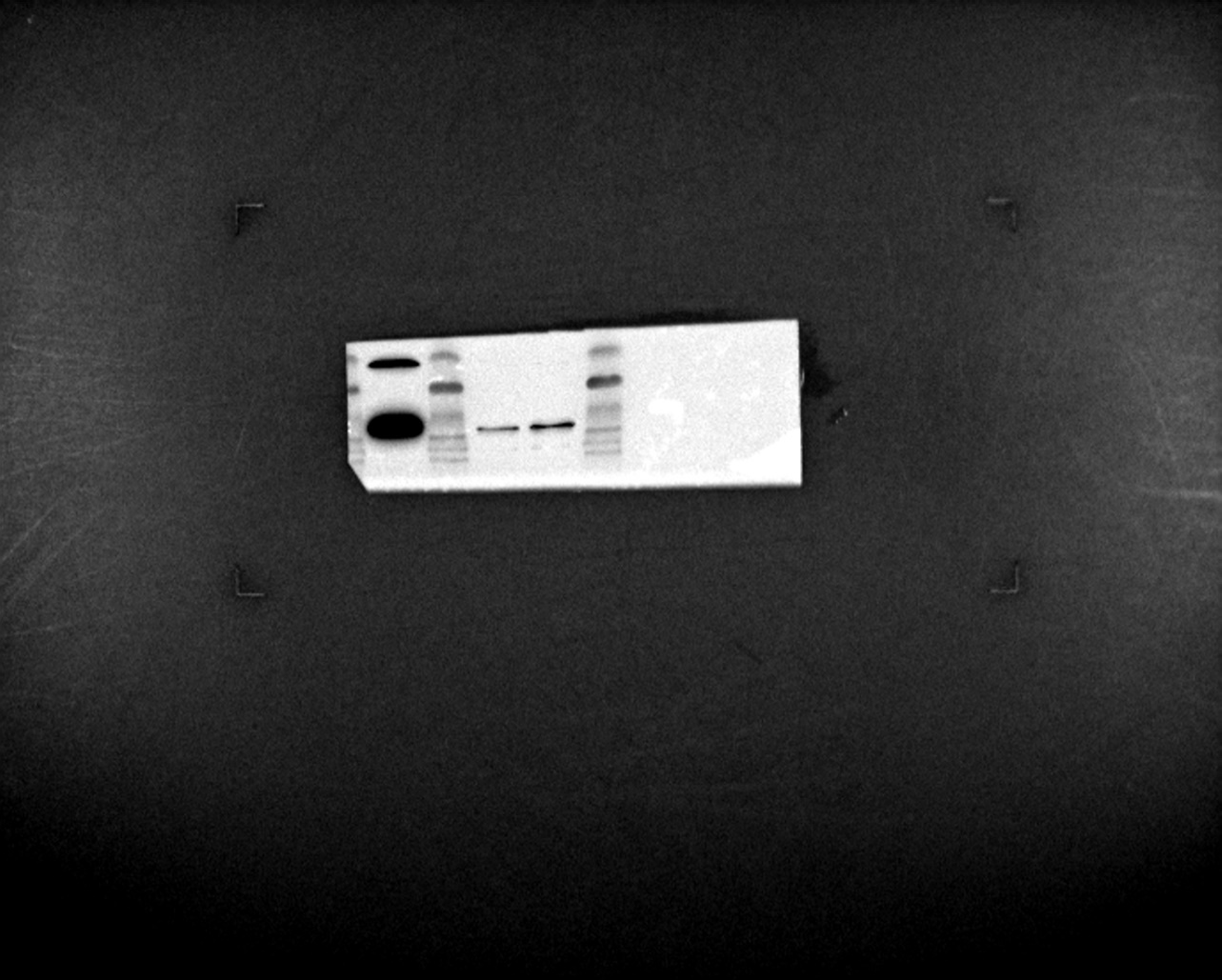


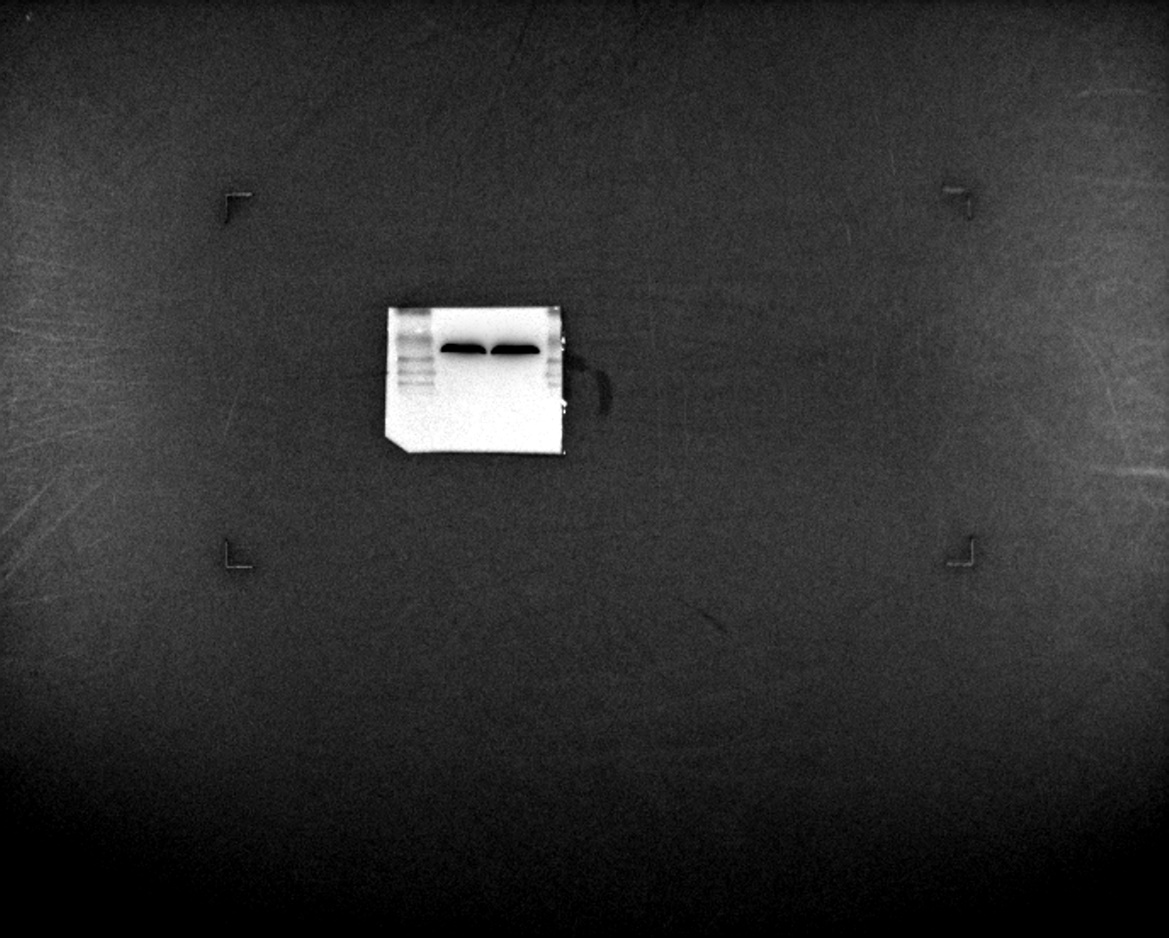

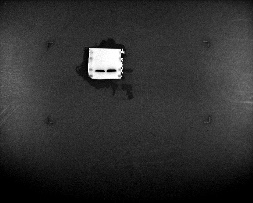


D:( USP11, RRM1, Actin)


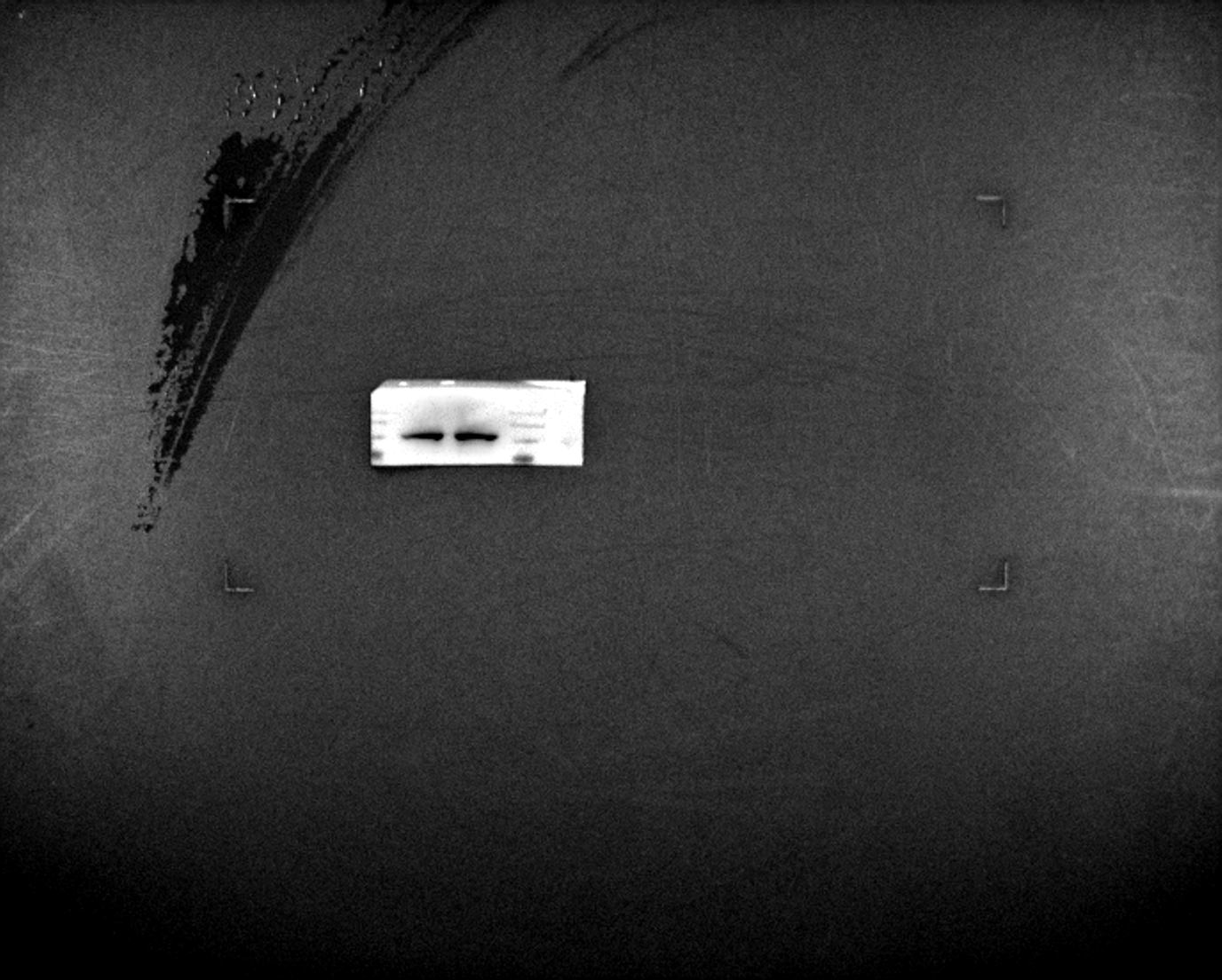

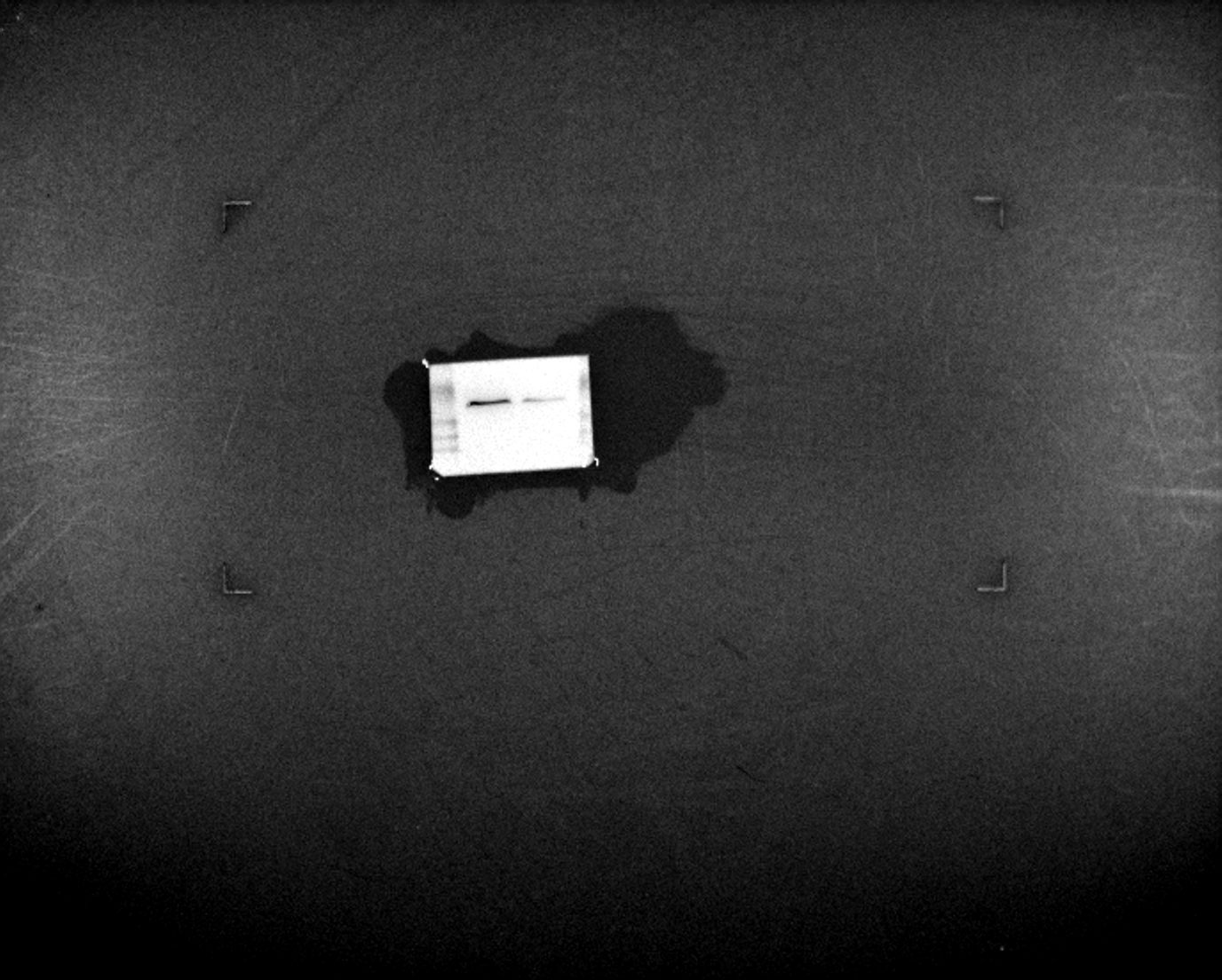

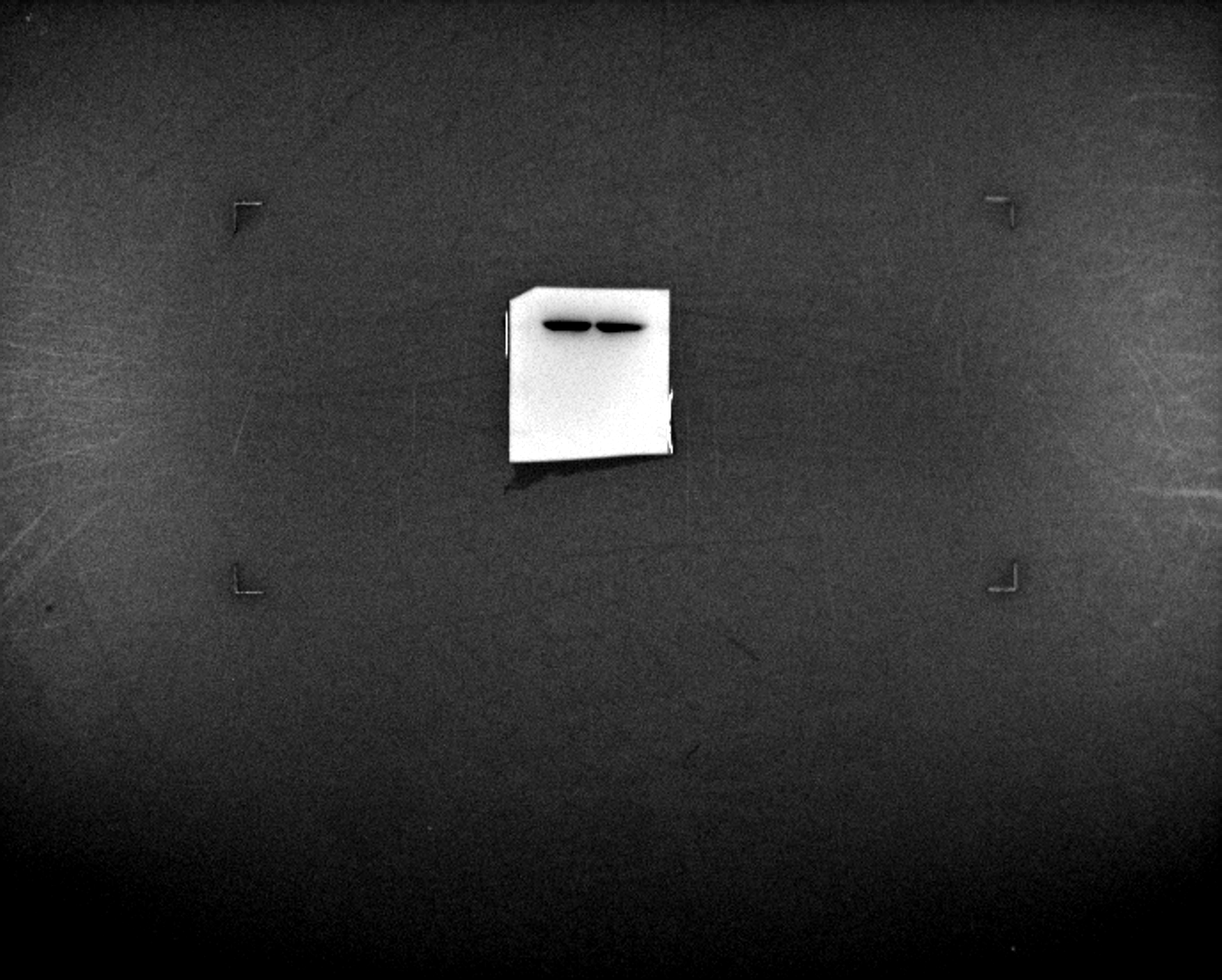


F:( IP-USP11, IP-E2F1, Input-USP11, Input-RRM1, Input-E2F1,RRM1, Input-Actin)


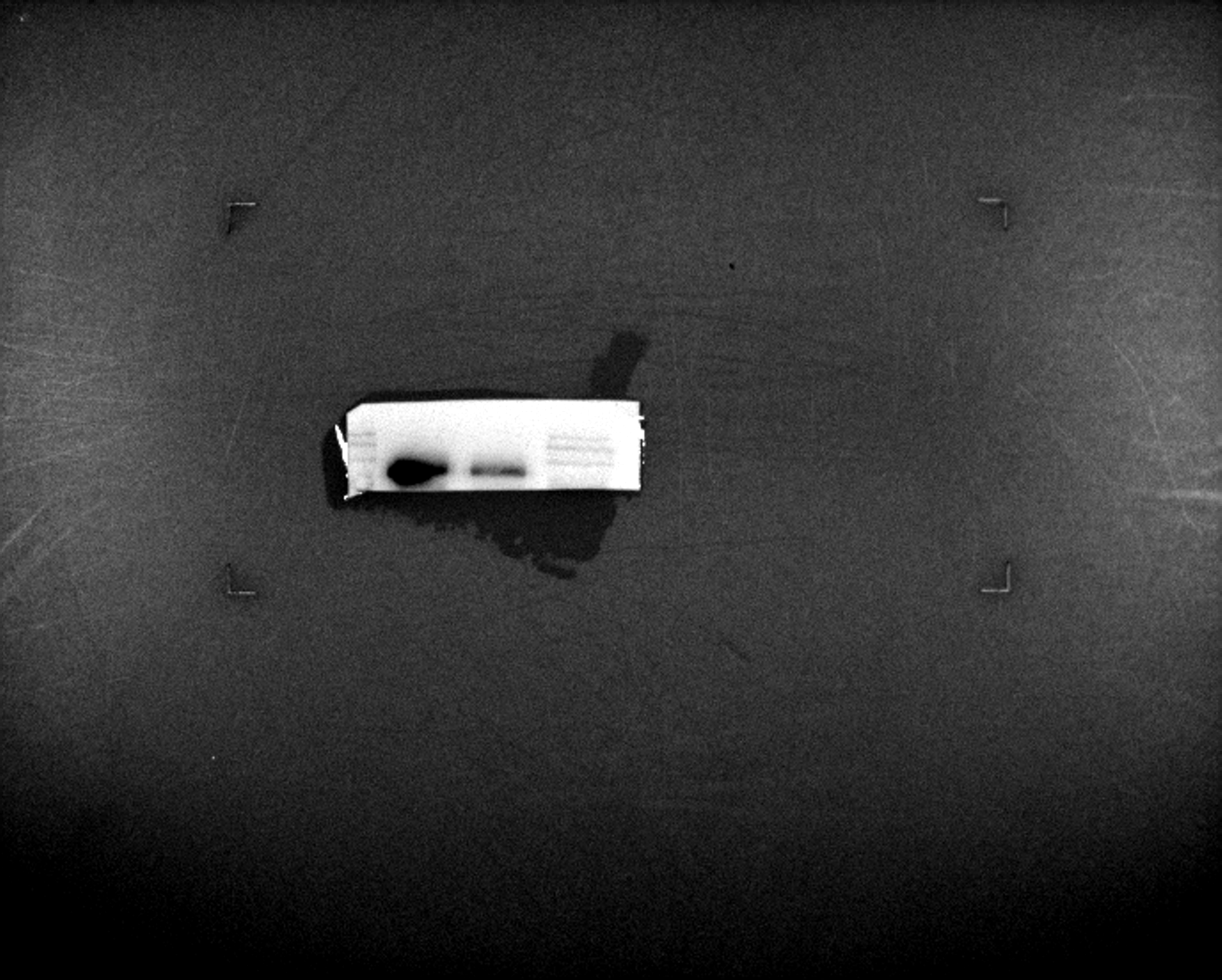

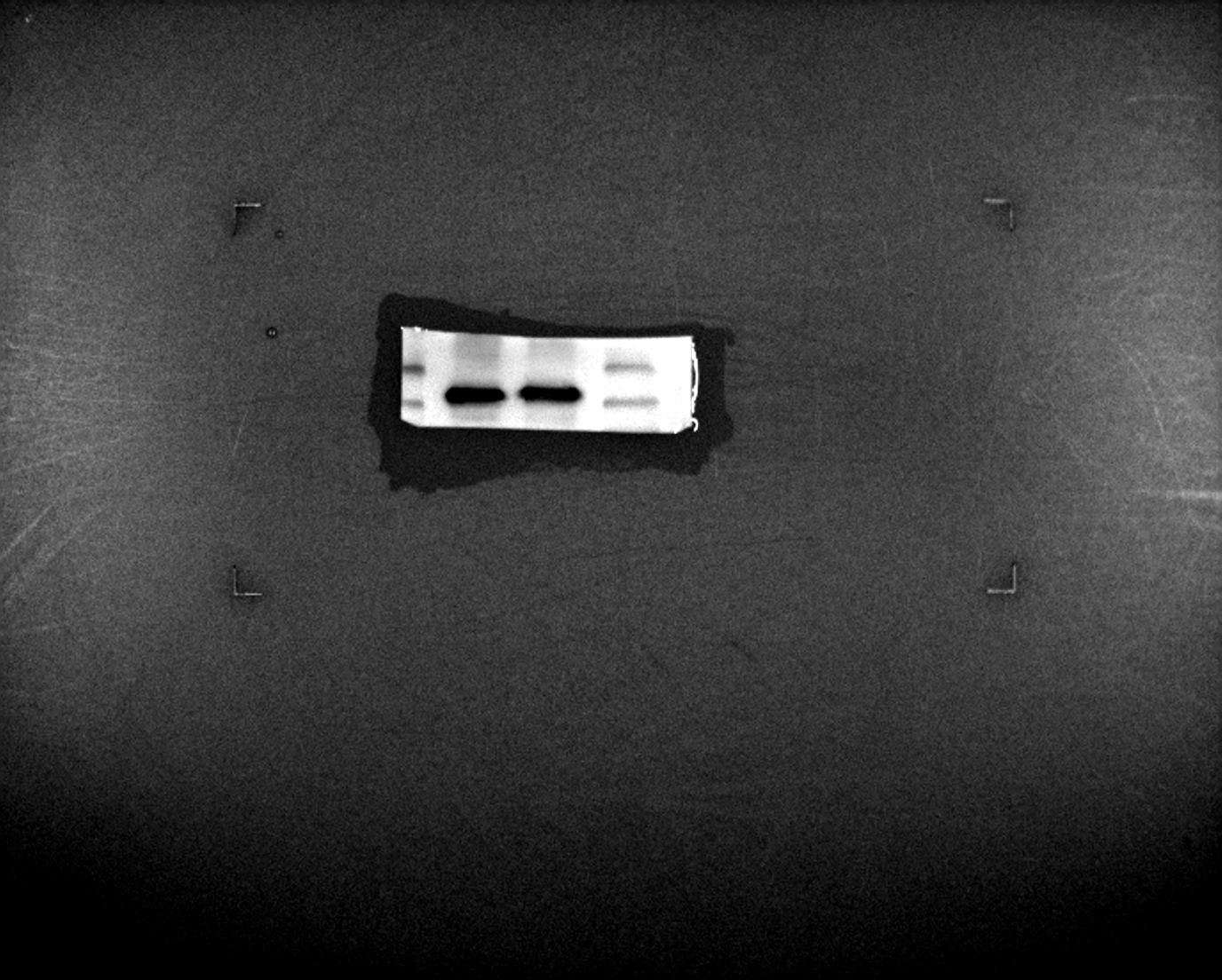

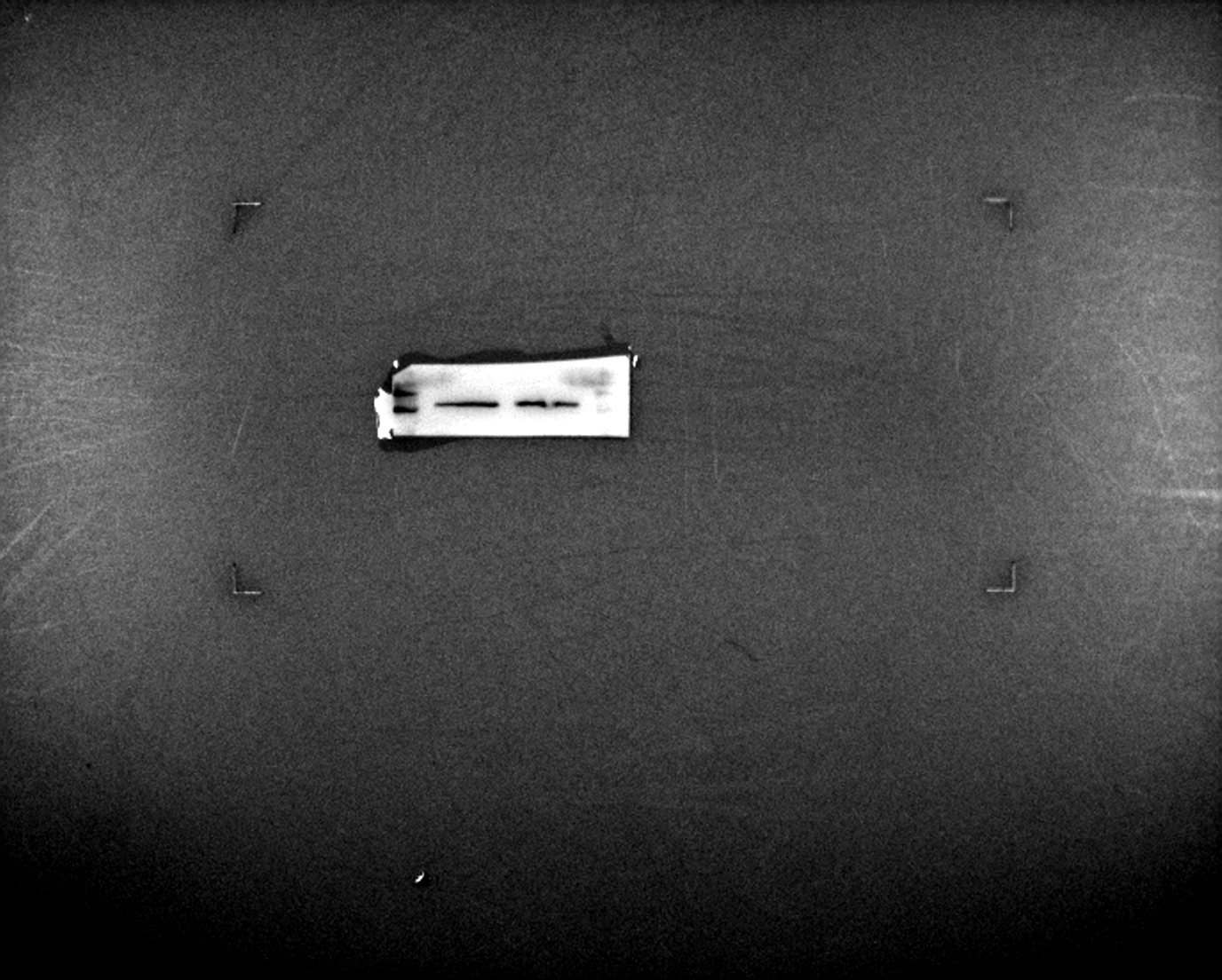


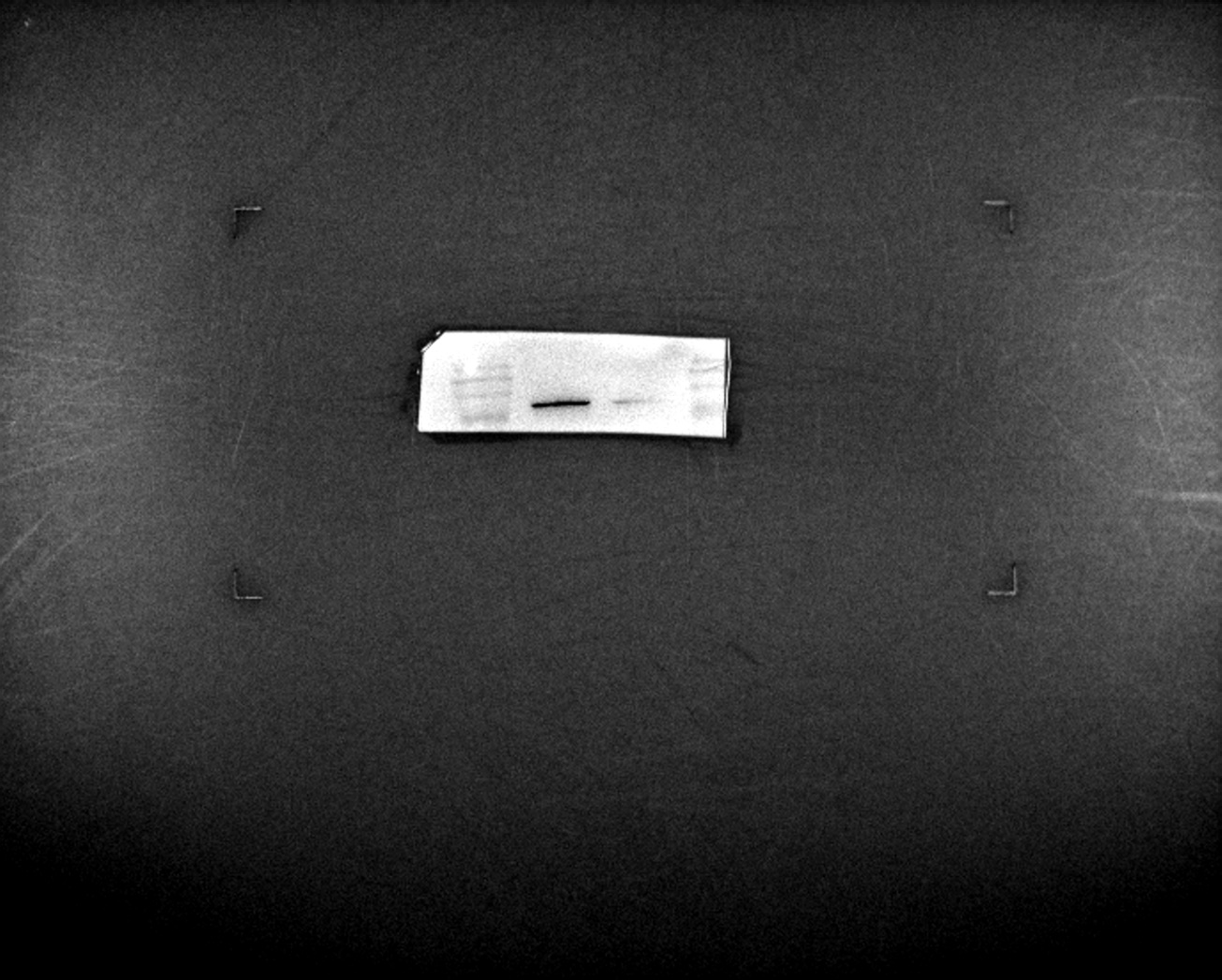

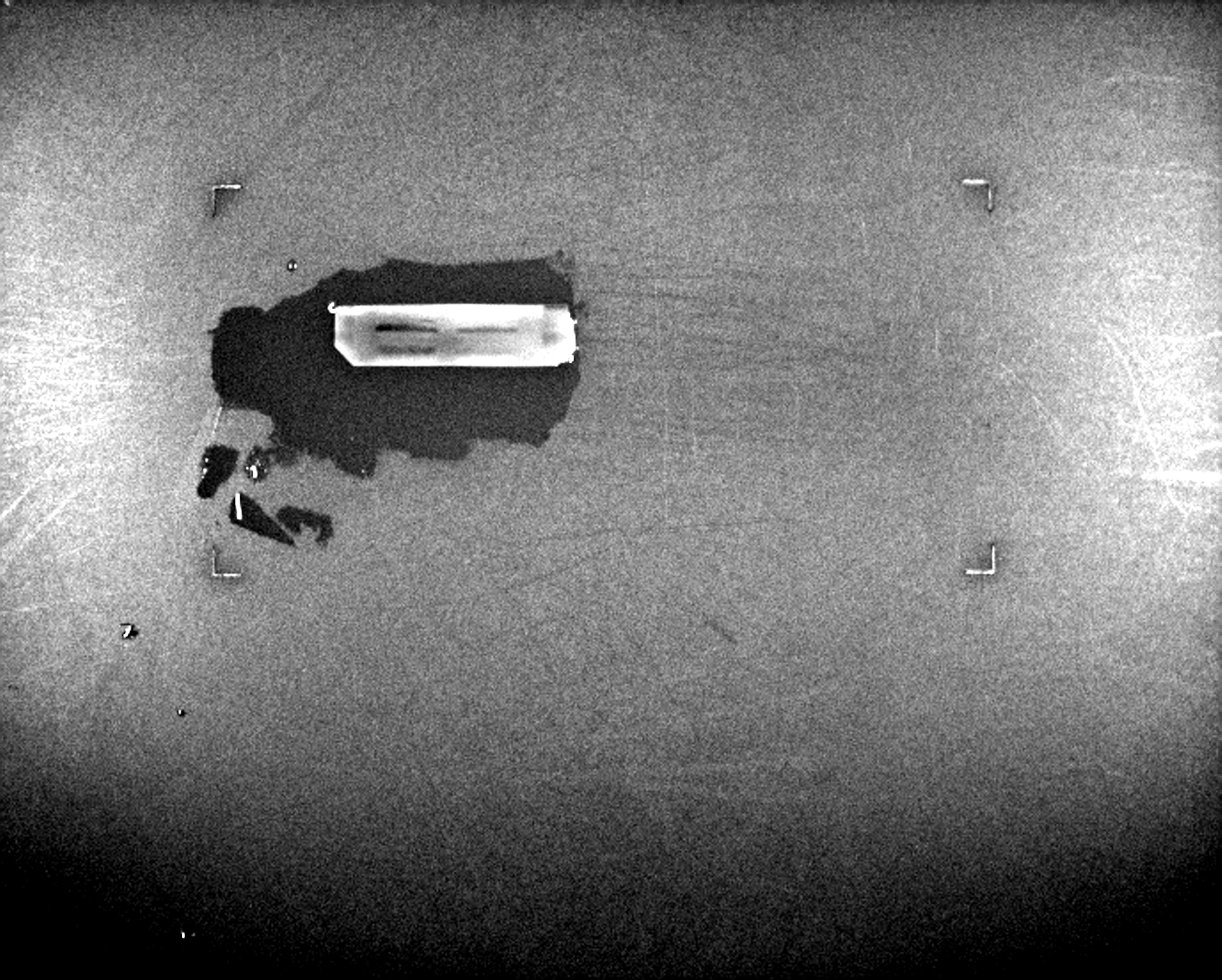

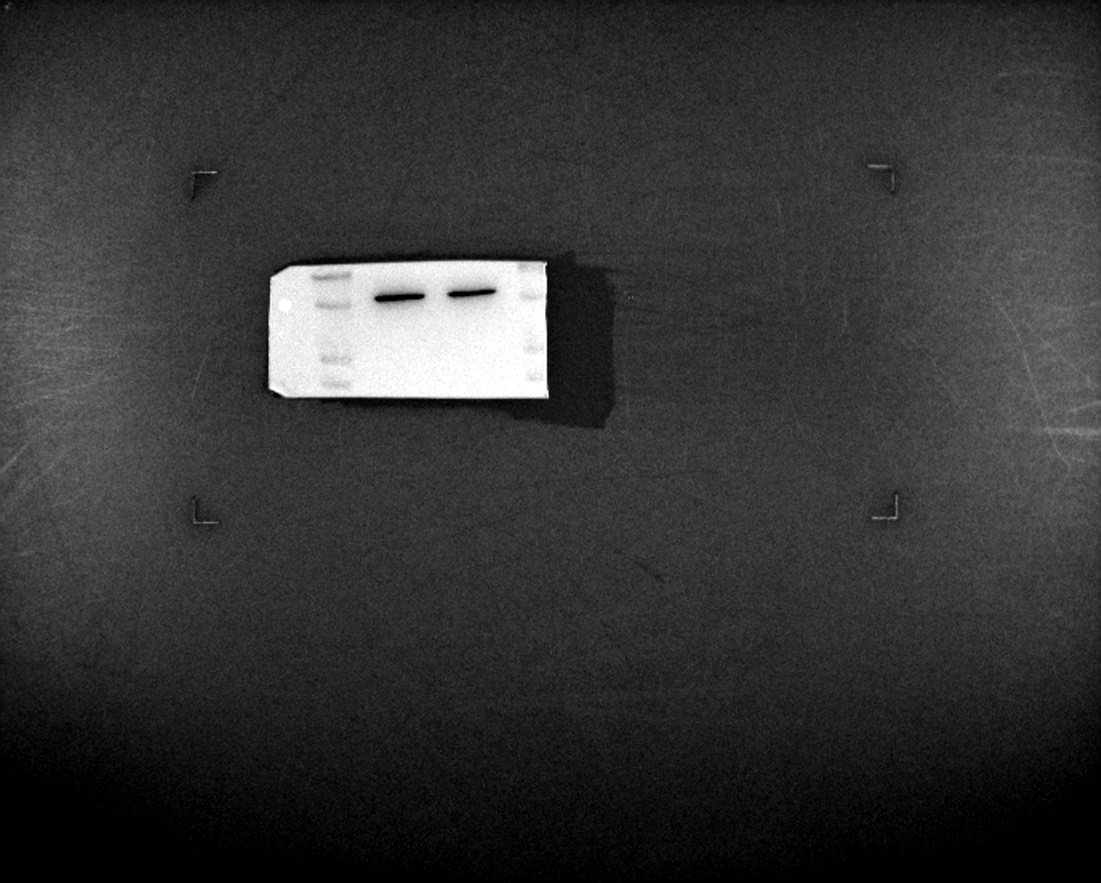


G:(USP11-N, USP11-C, H3, Actin)


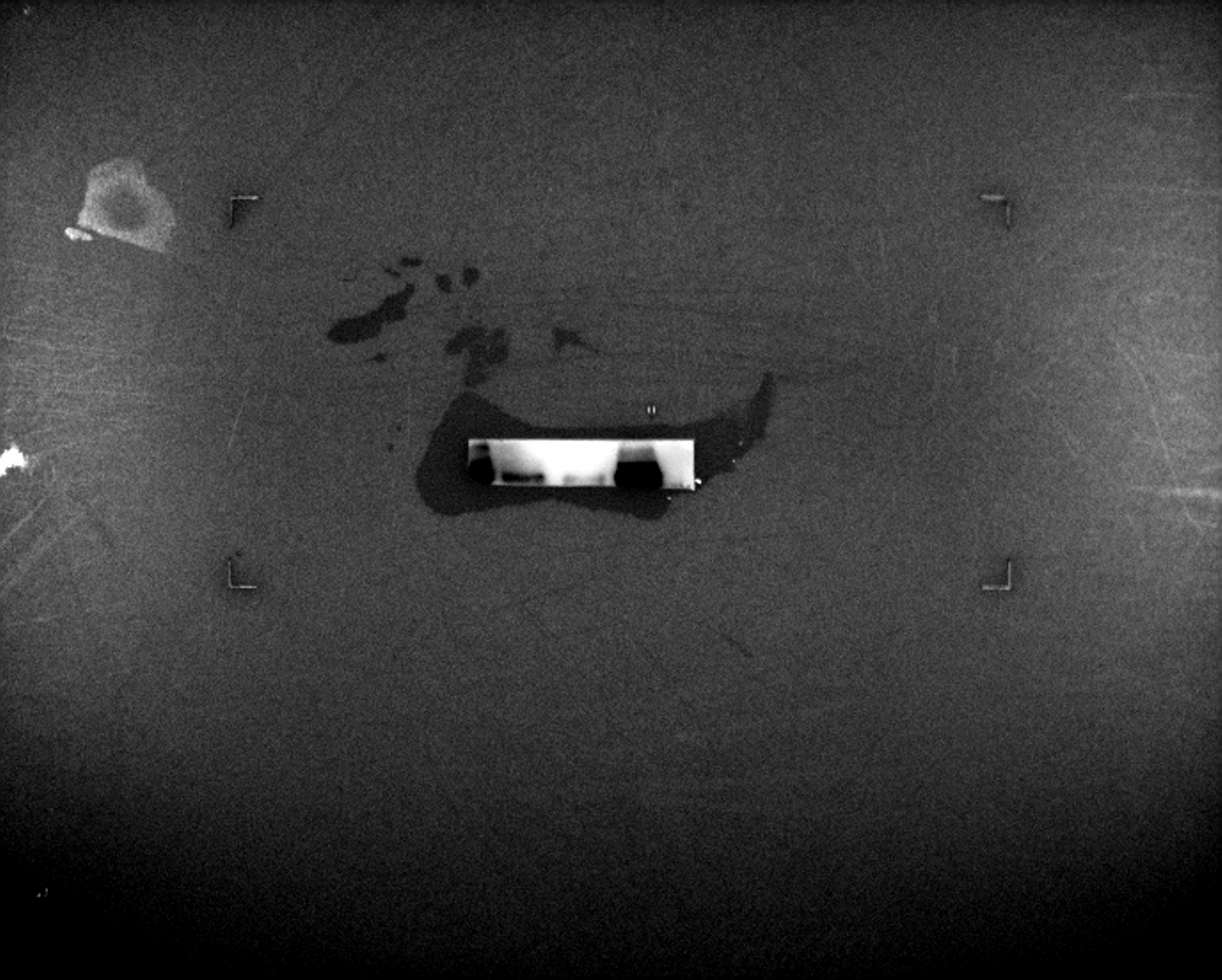

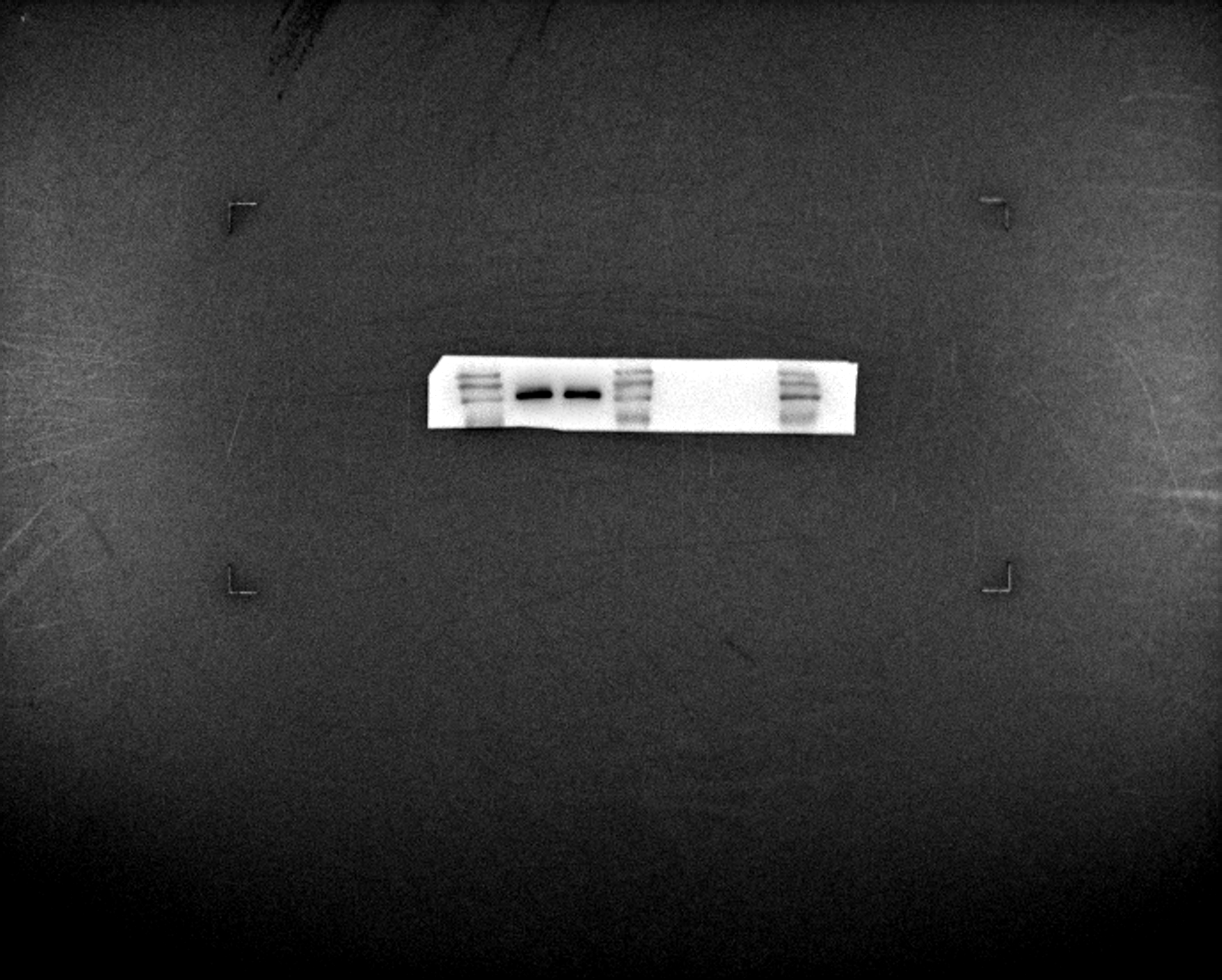


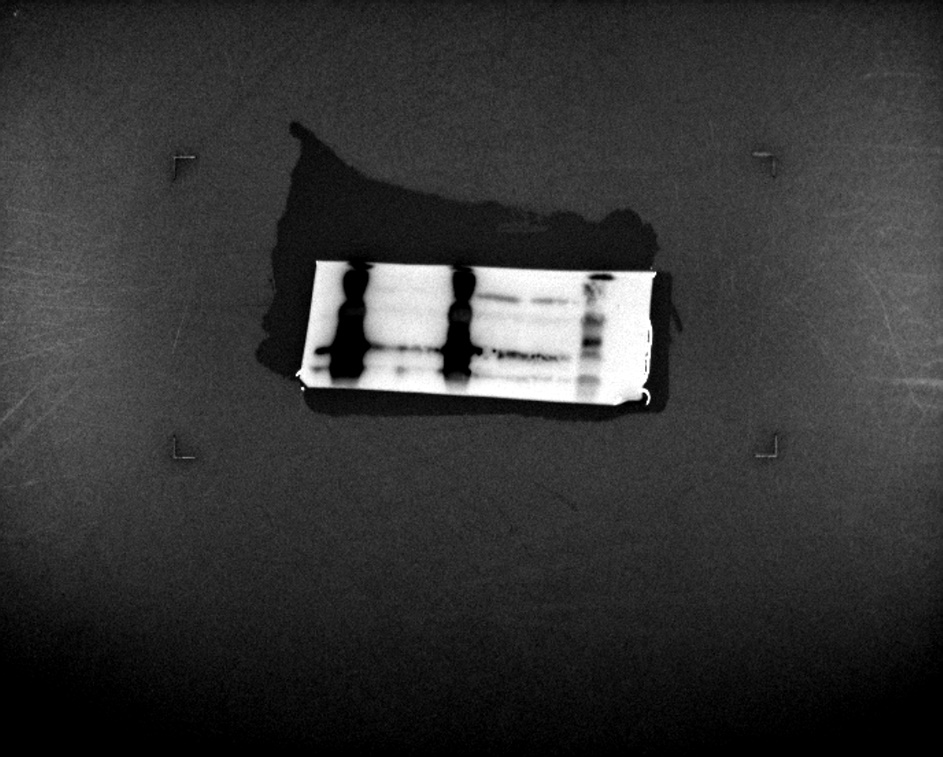

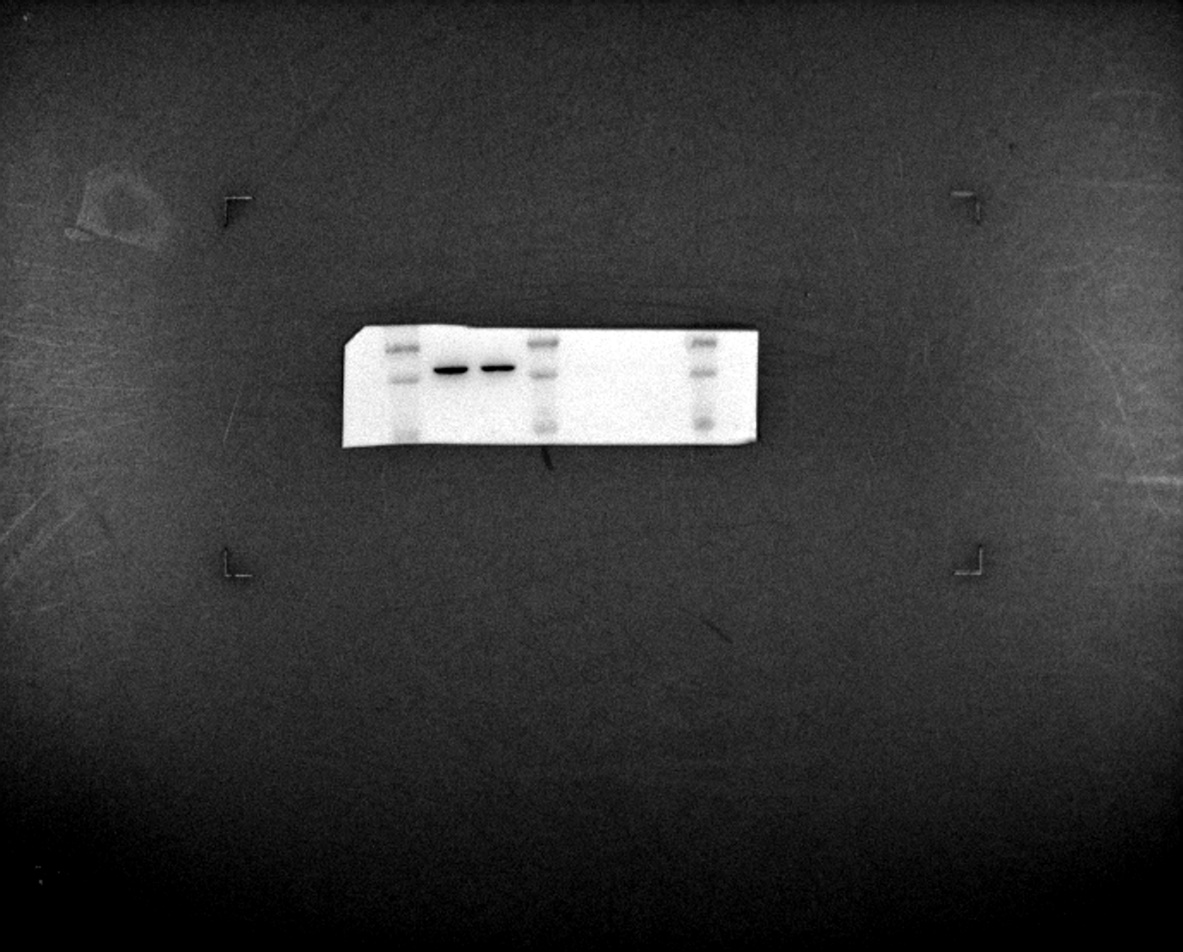


H:(USP11-C, USP11-N, H3, Actin)


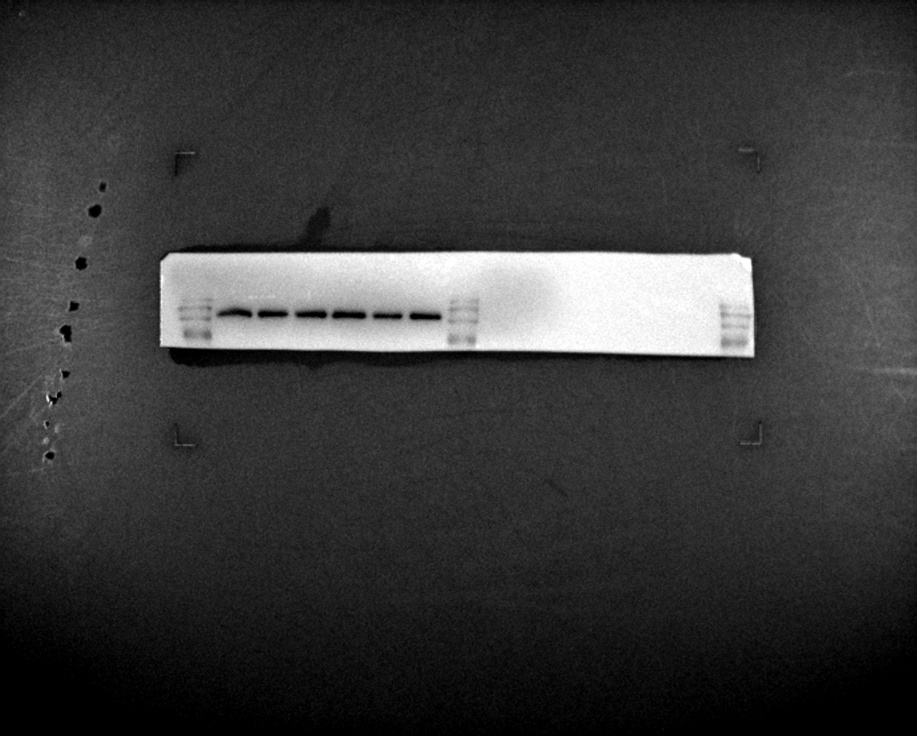

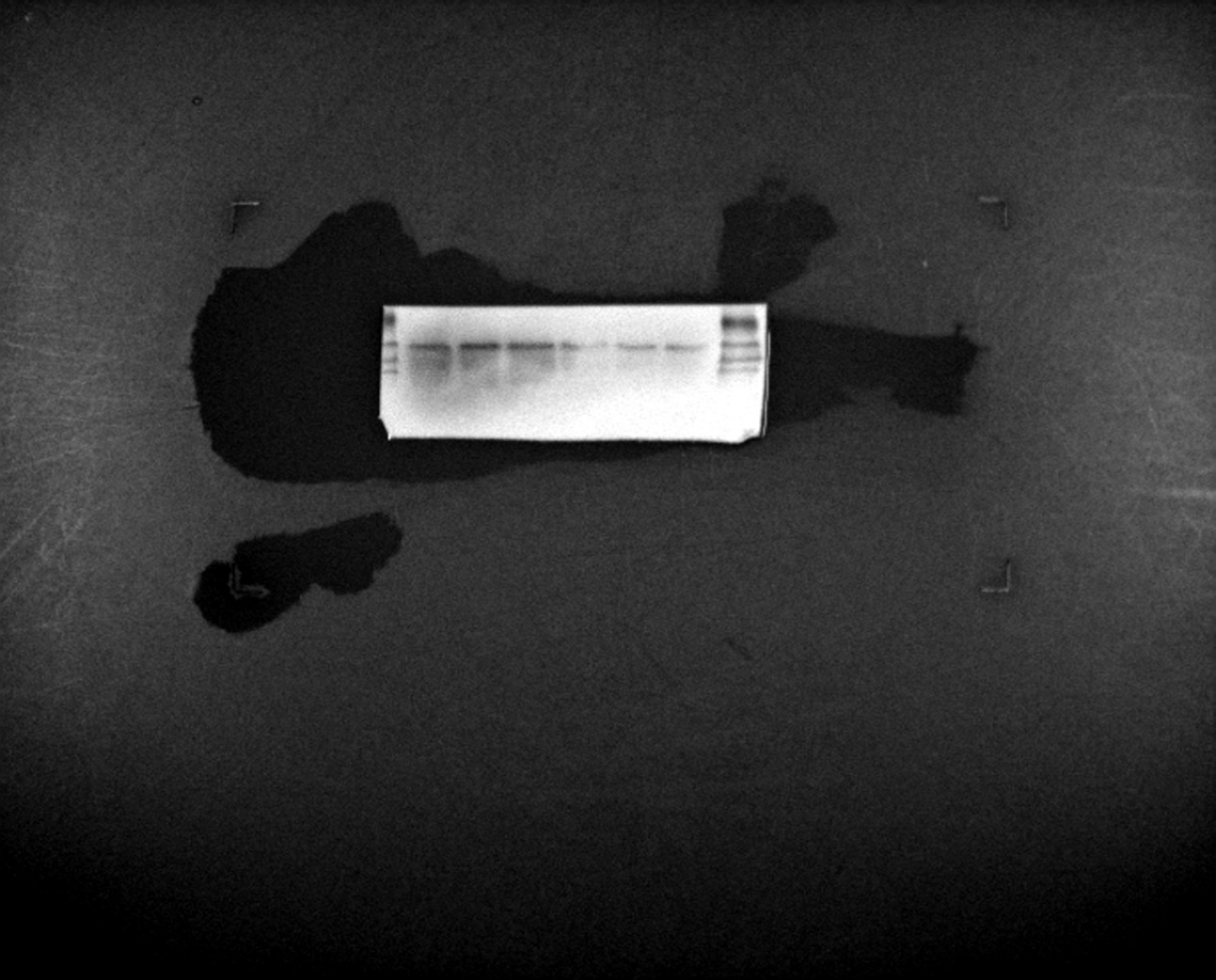

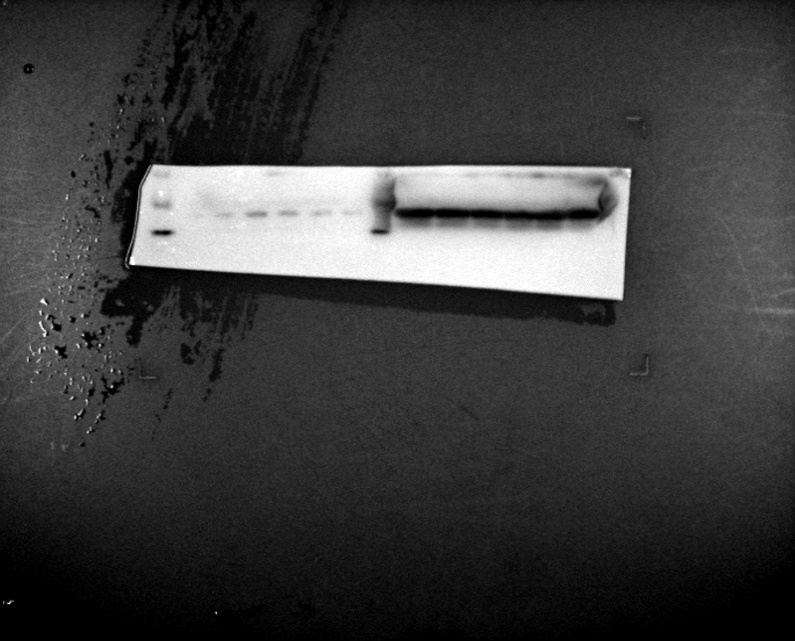

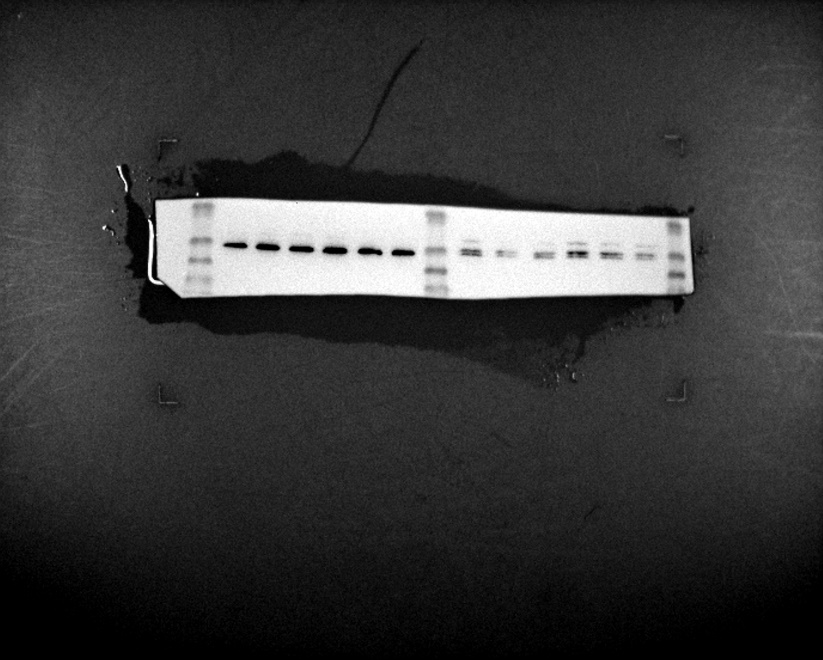


**Fig. SM5**

A:( RRM1,H3,GAPDH)


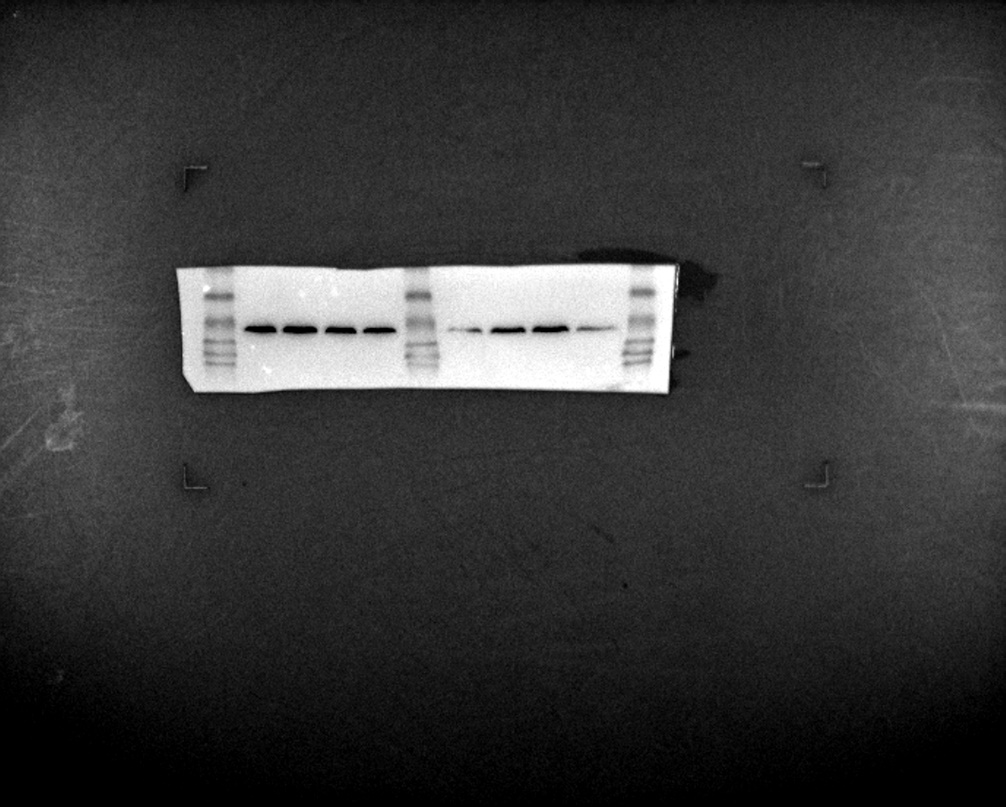

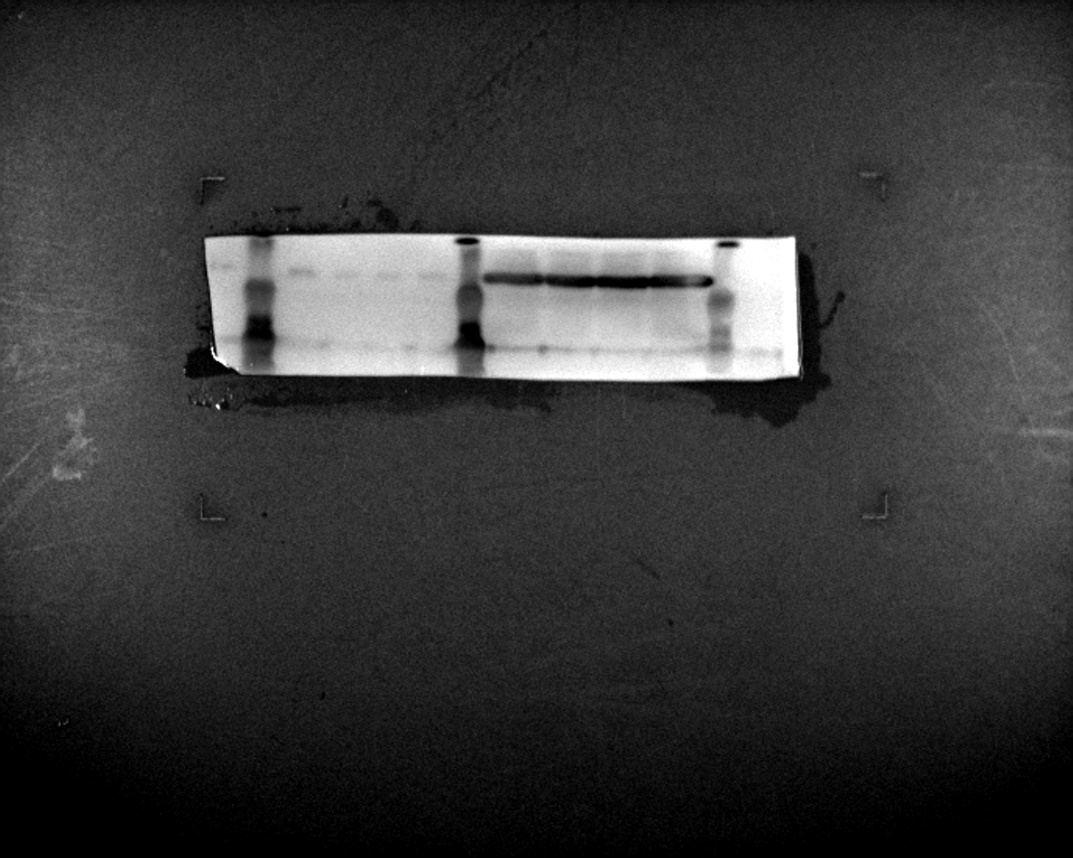

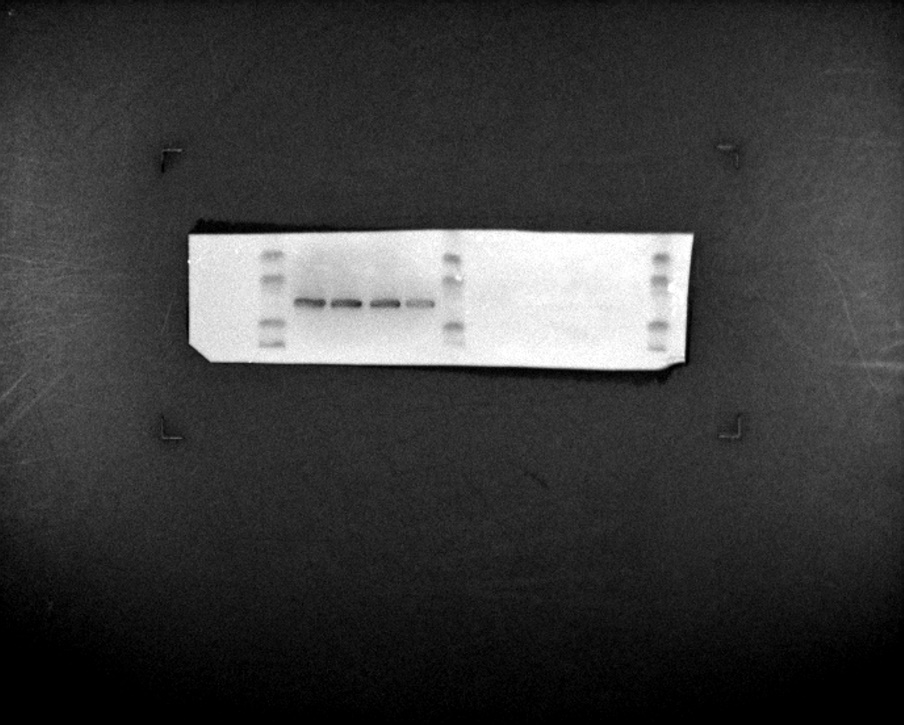


B:( RRM1,LaminB1, NUP50)


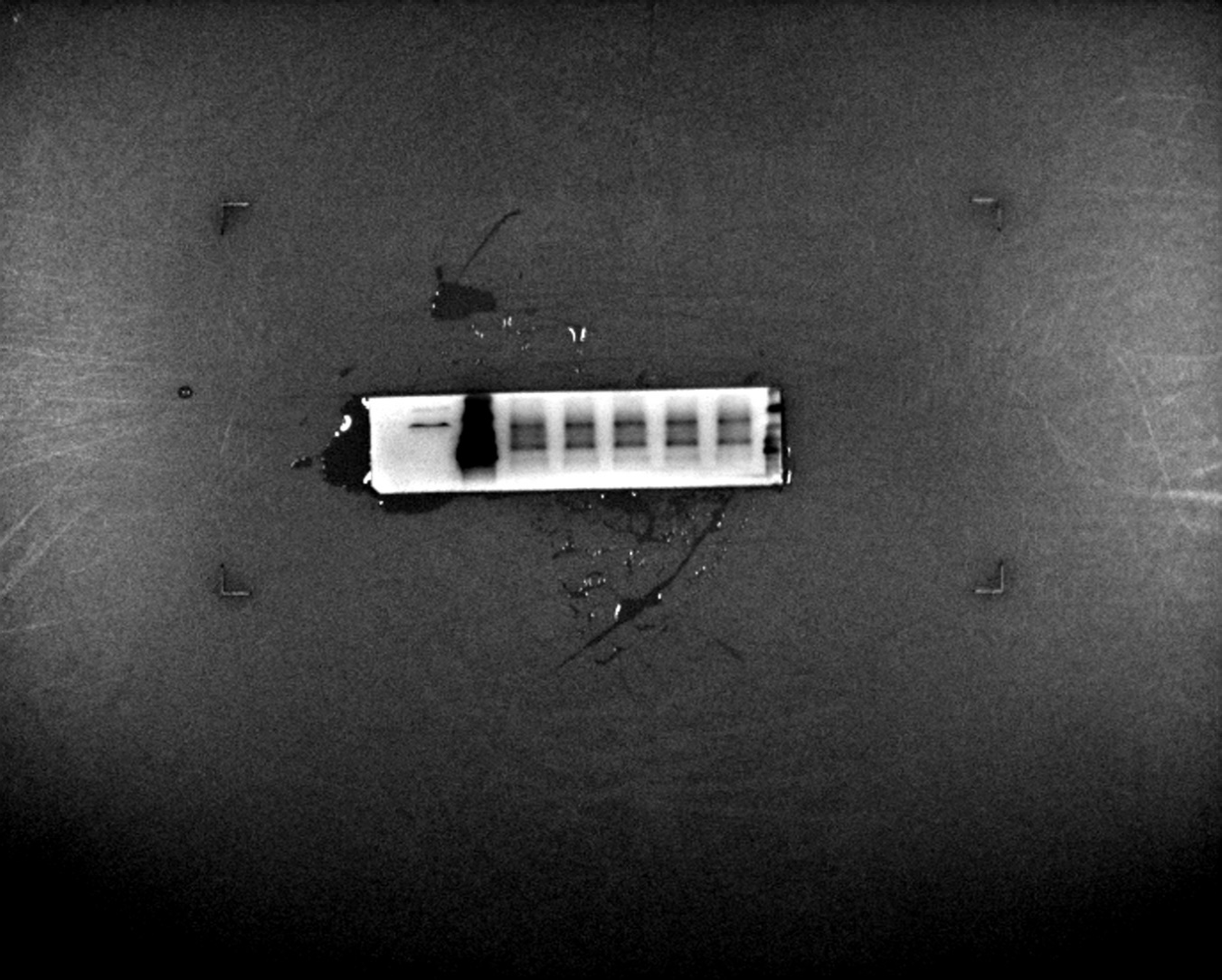

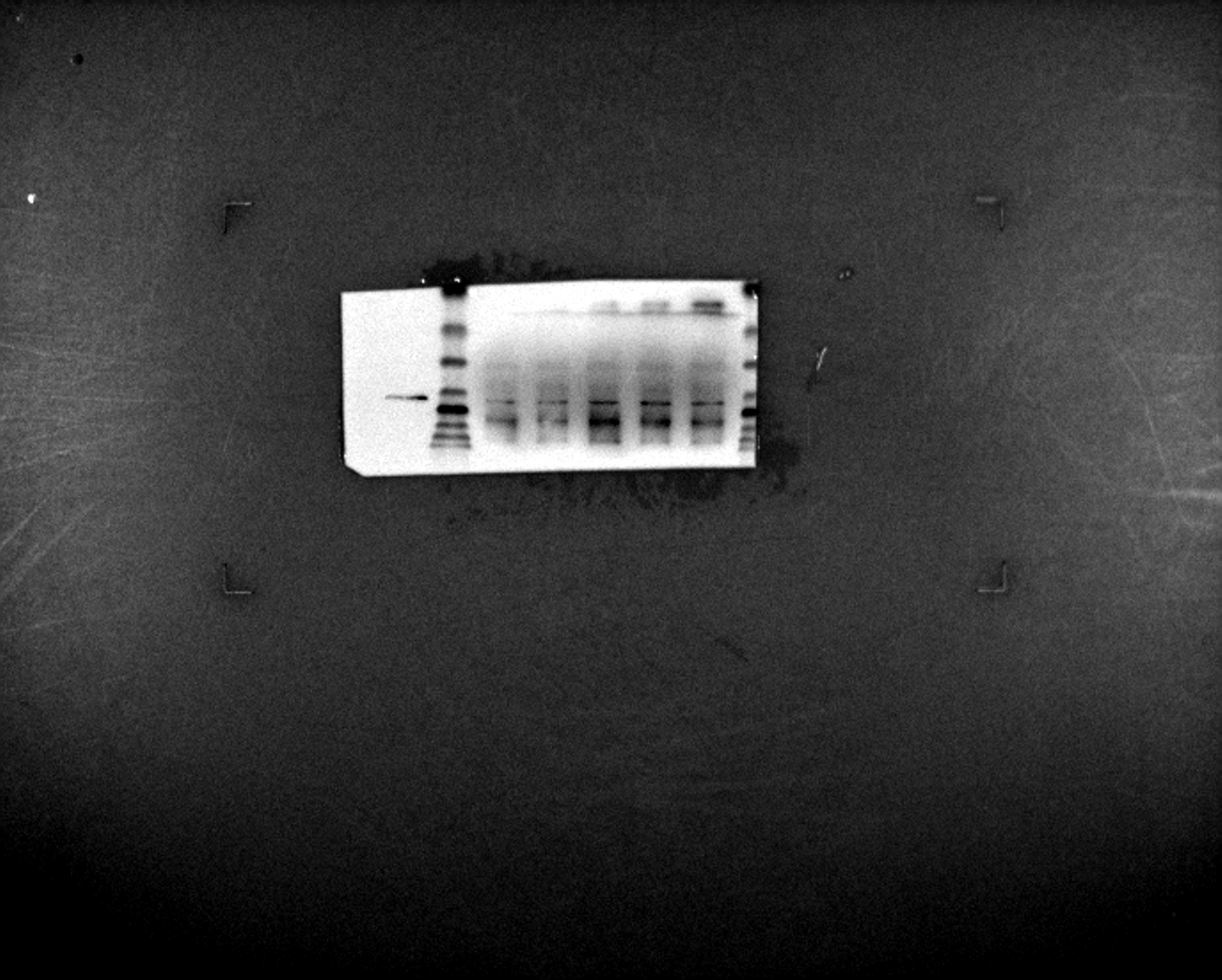


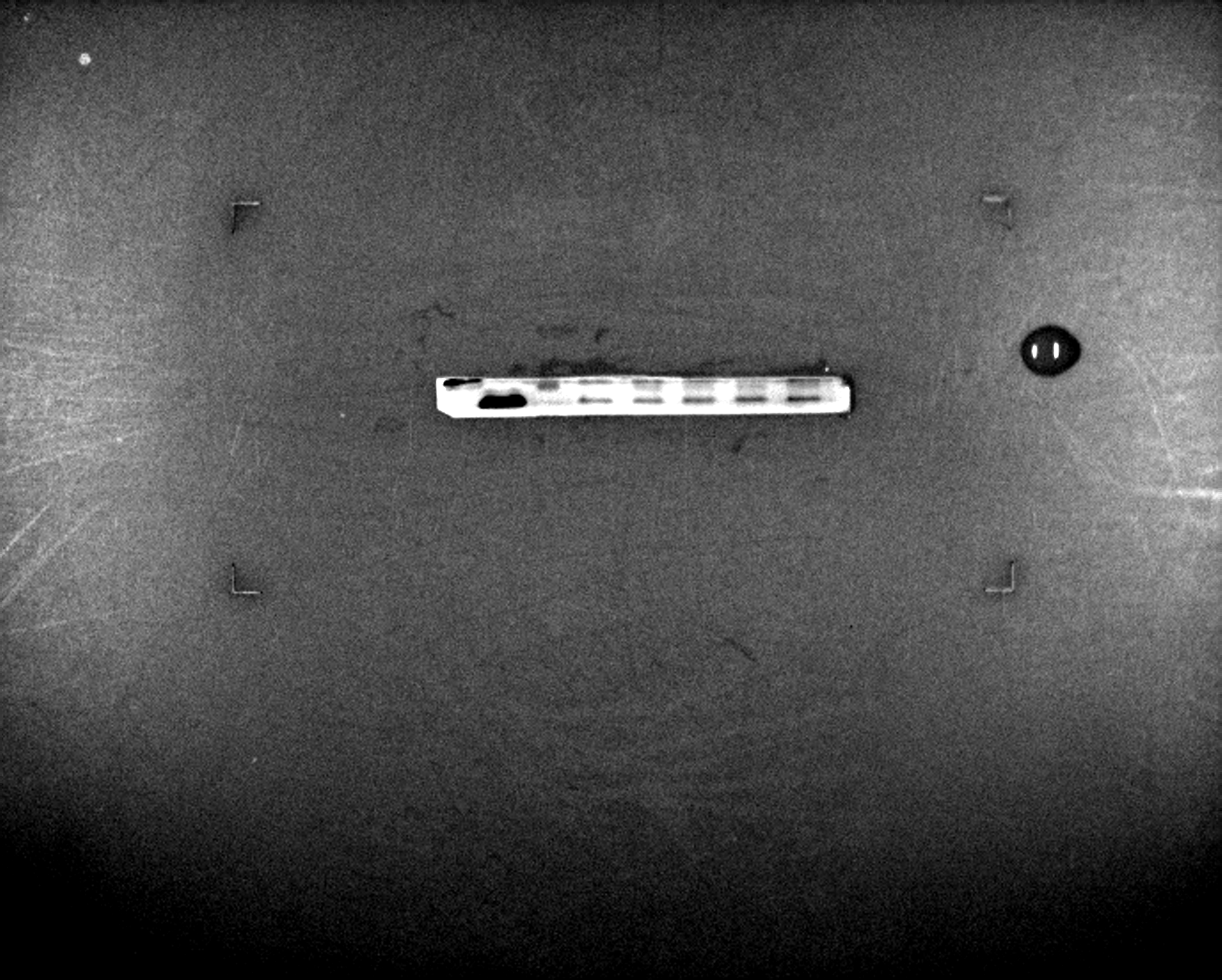


E:( USP11,RRM1,H3,Actin)


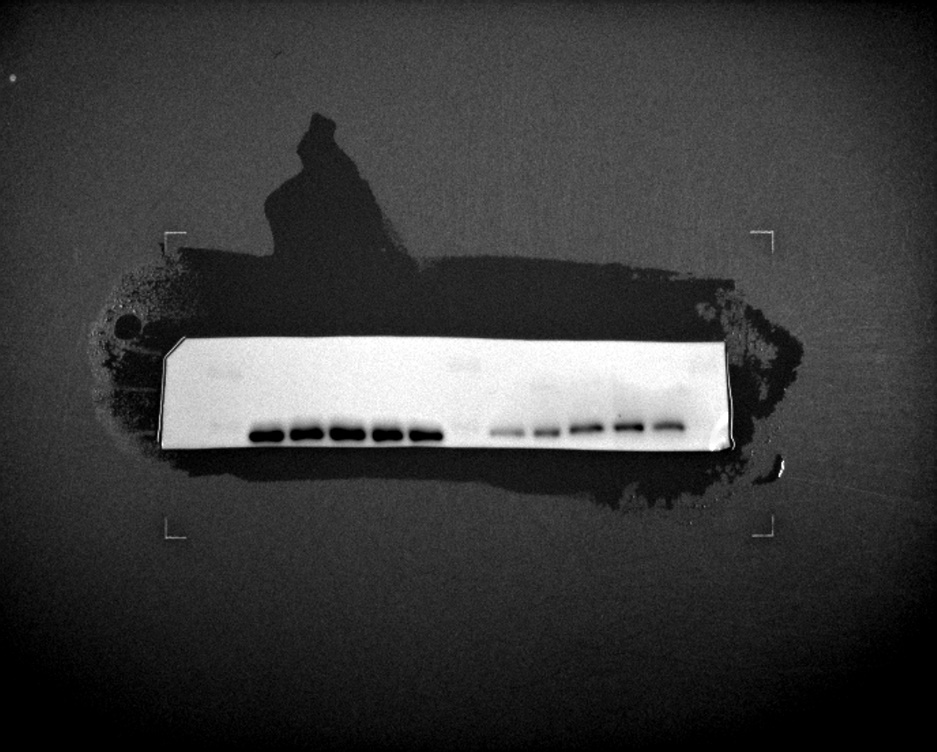

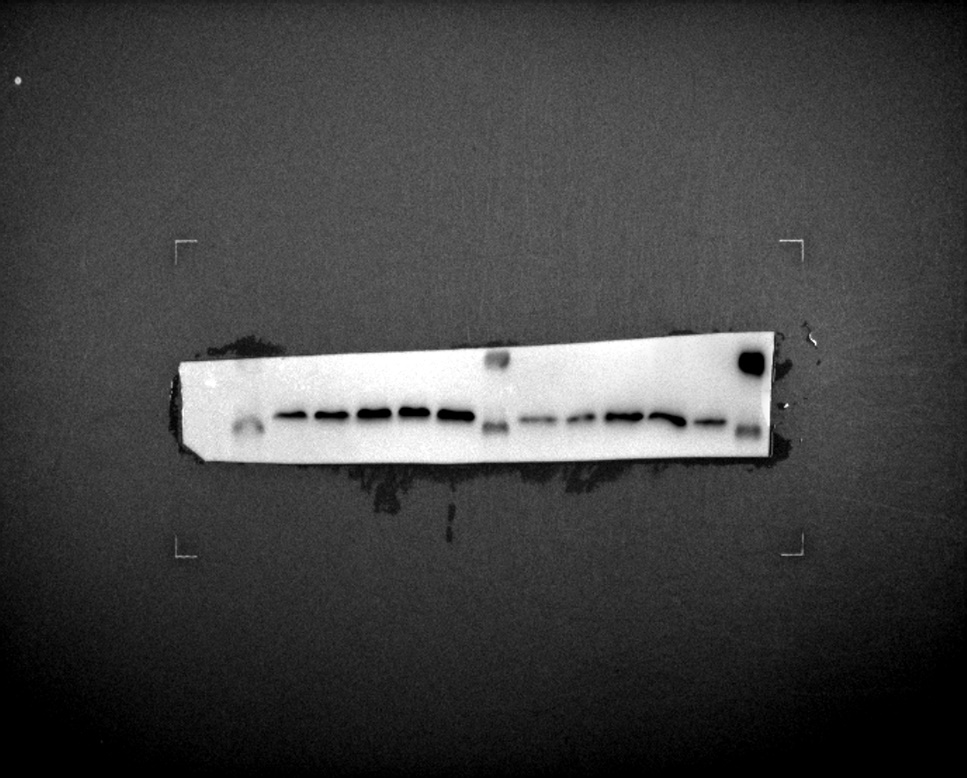


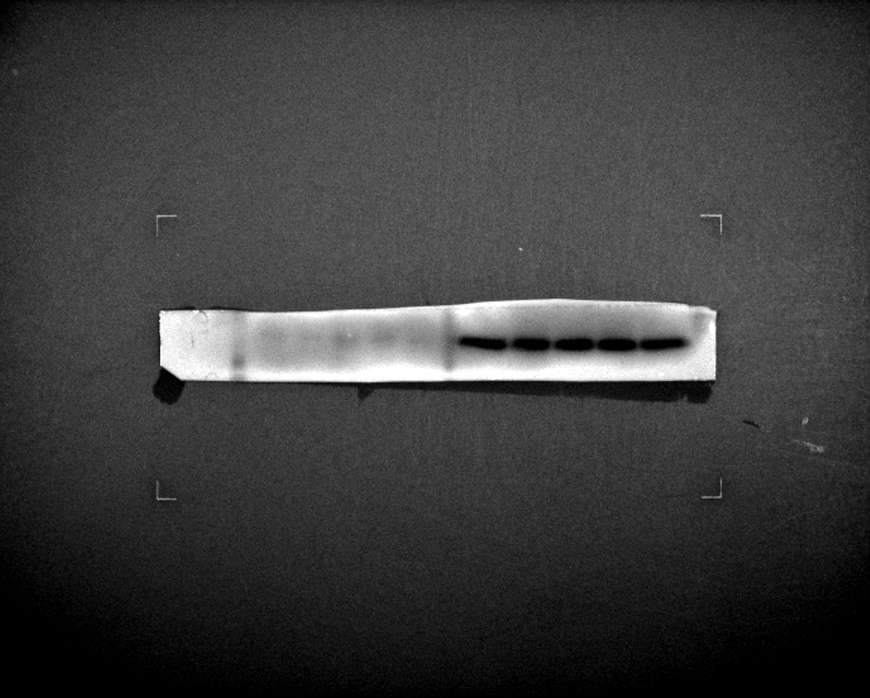

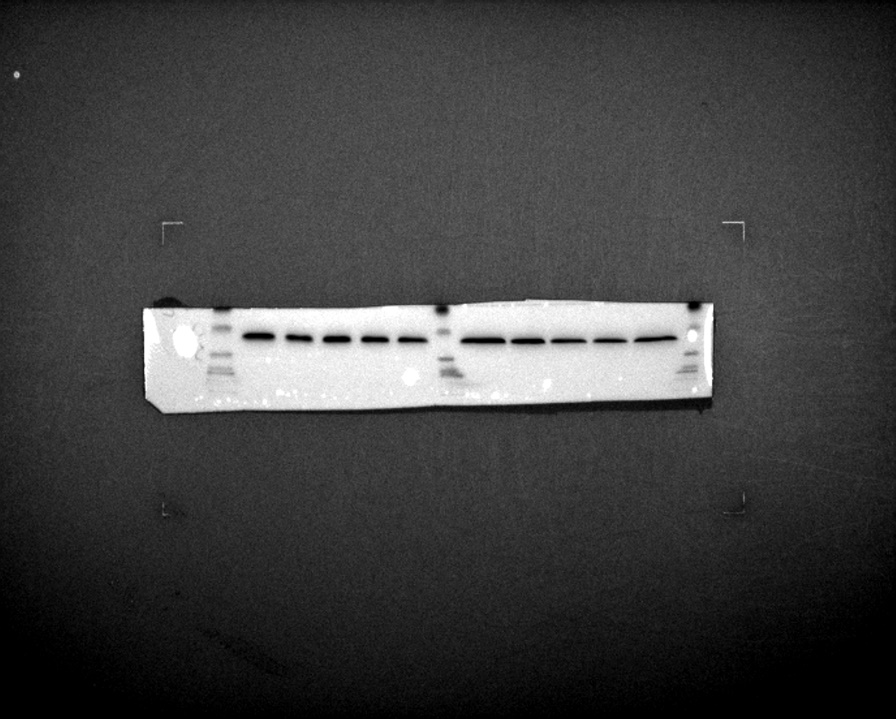


G:( USP11, SEC13, NUP85, NUP50)


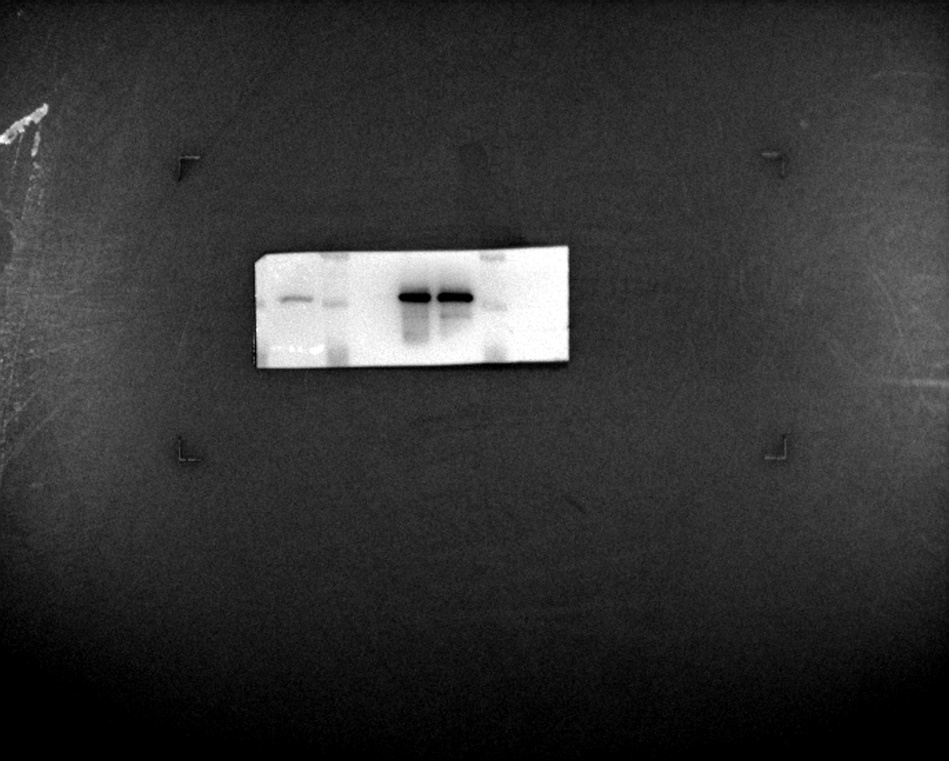

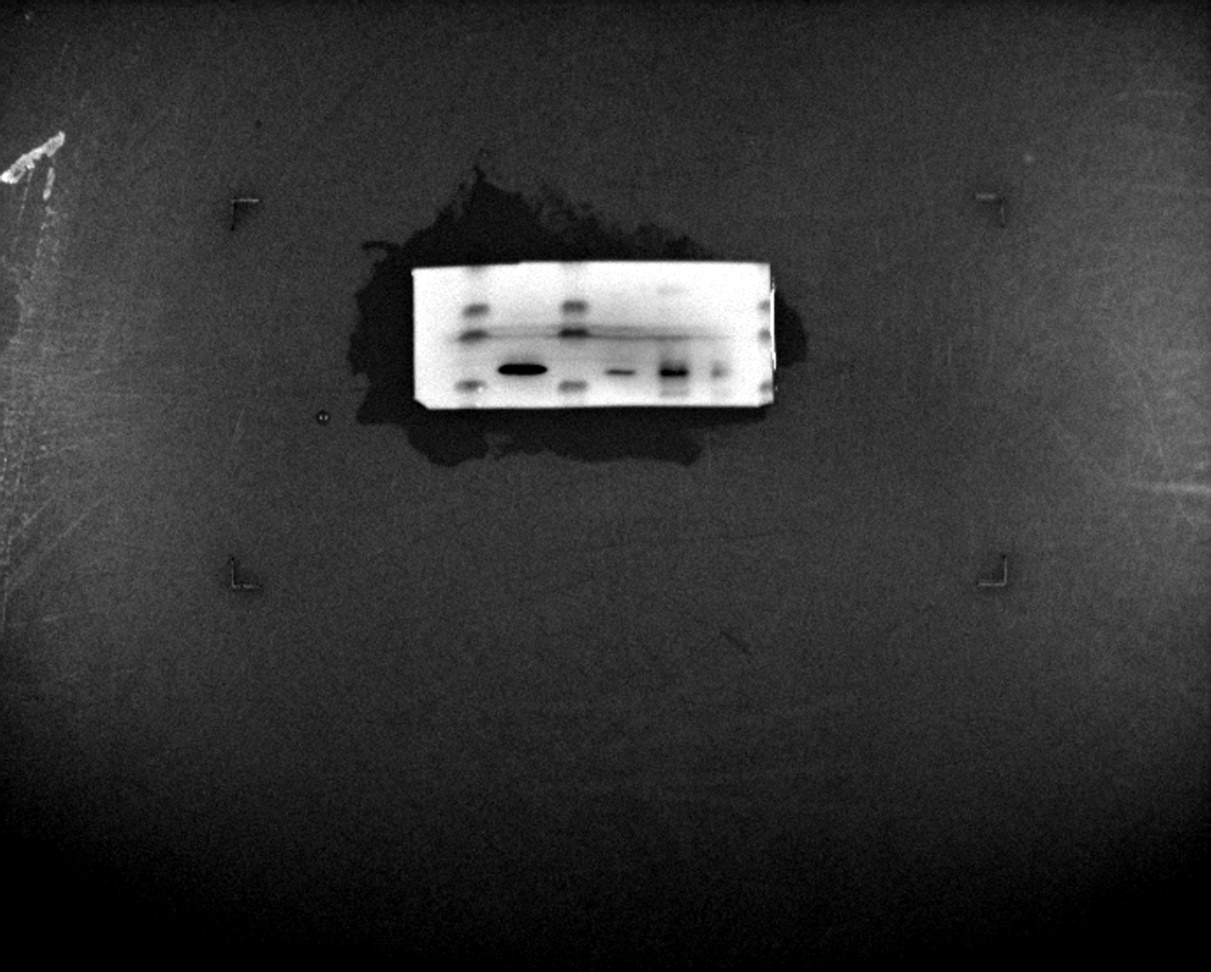

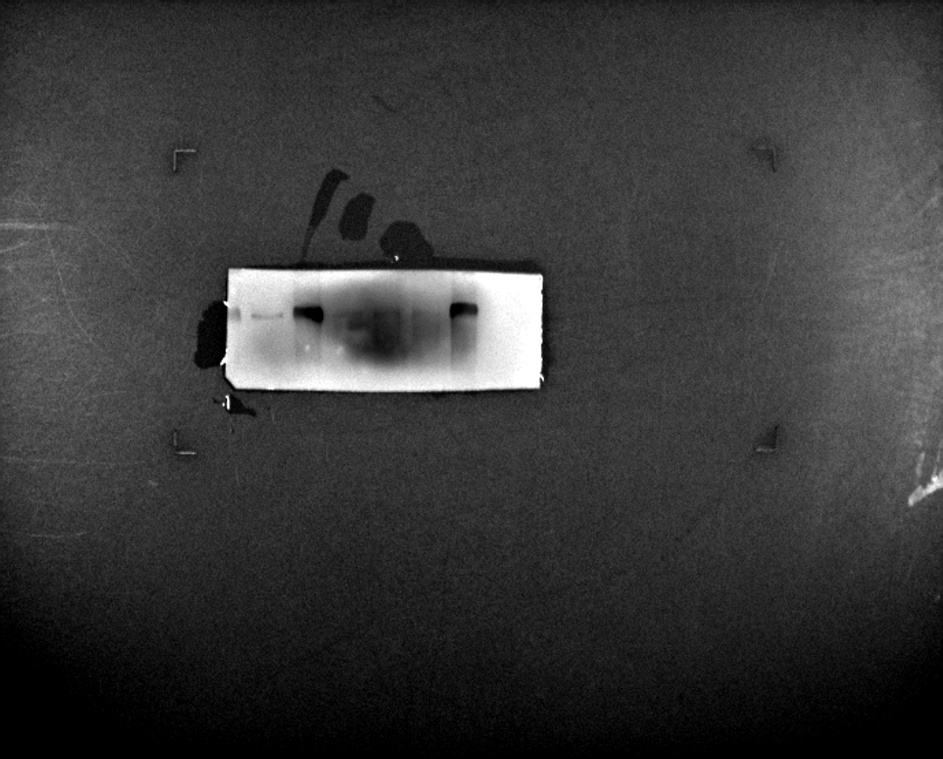


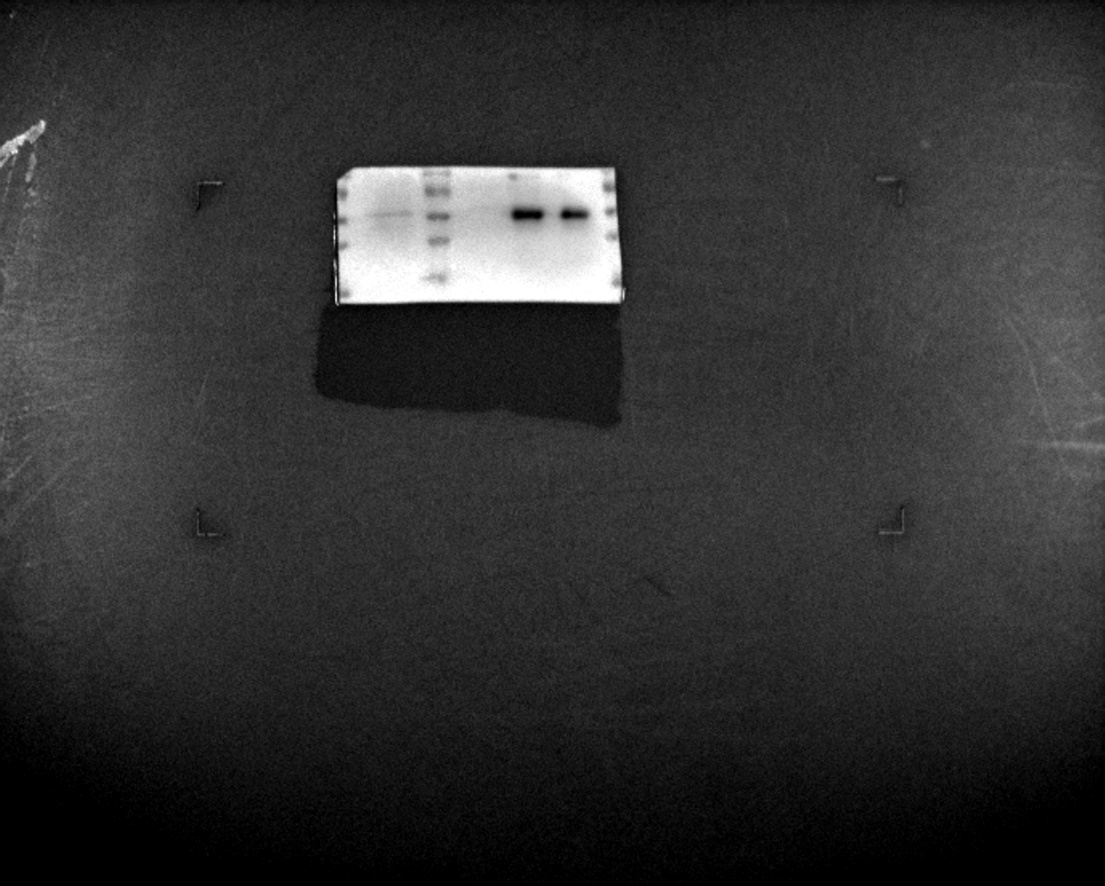


**Fig. SM6**

E:( IP-USP11, IP-Flag, Input-USP11, Input-RRM1, Input-Flag, Input-Actin, IP-LaminB1, input-LaminB1)


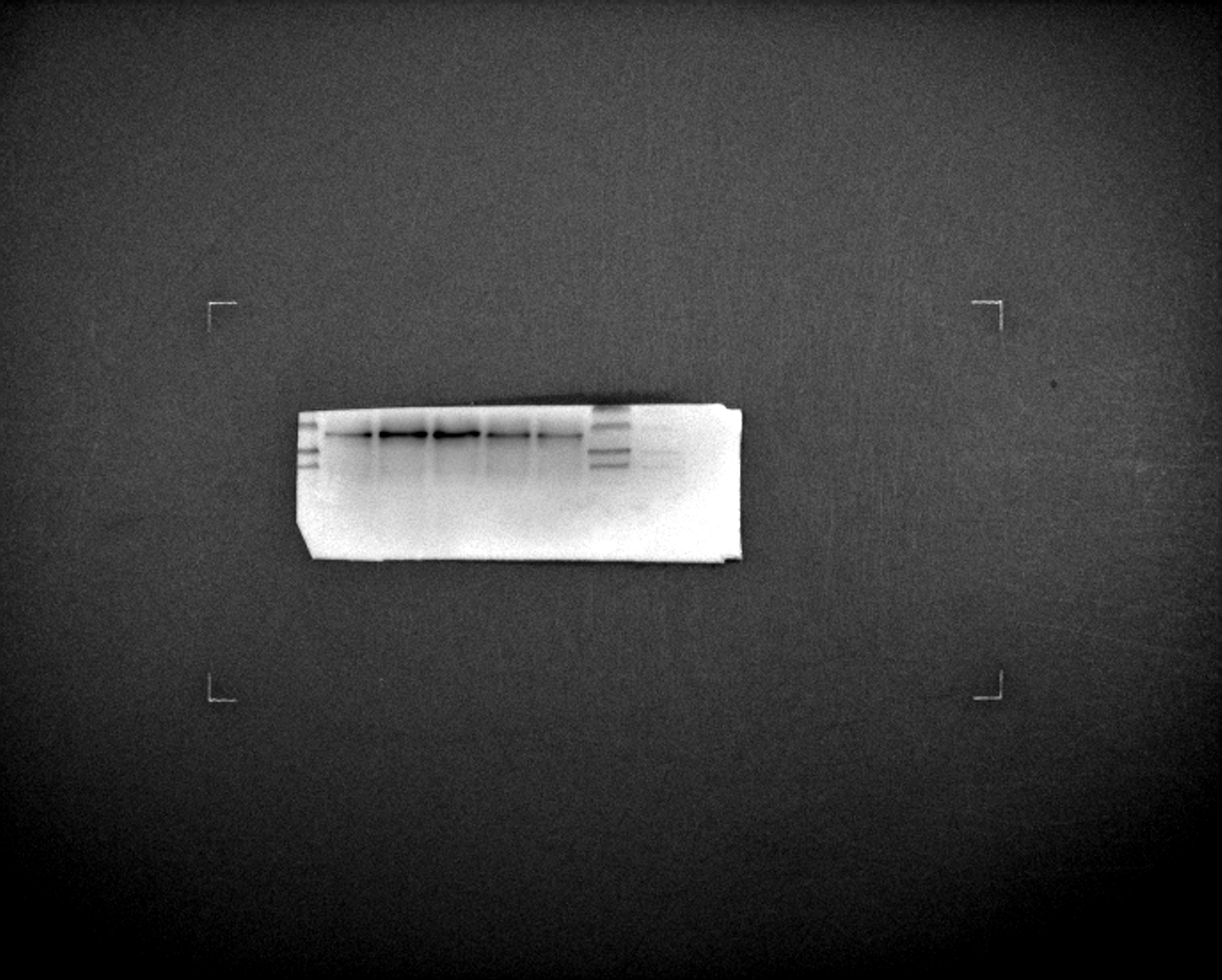

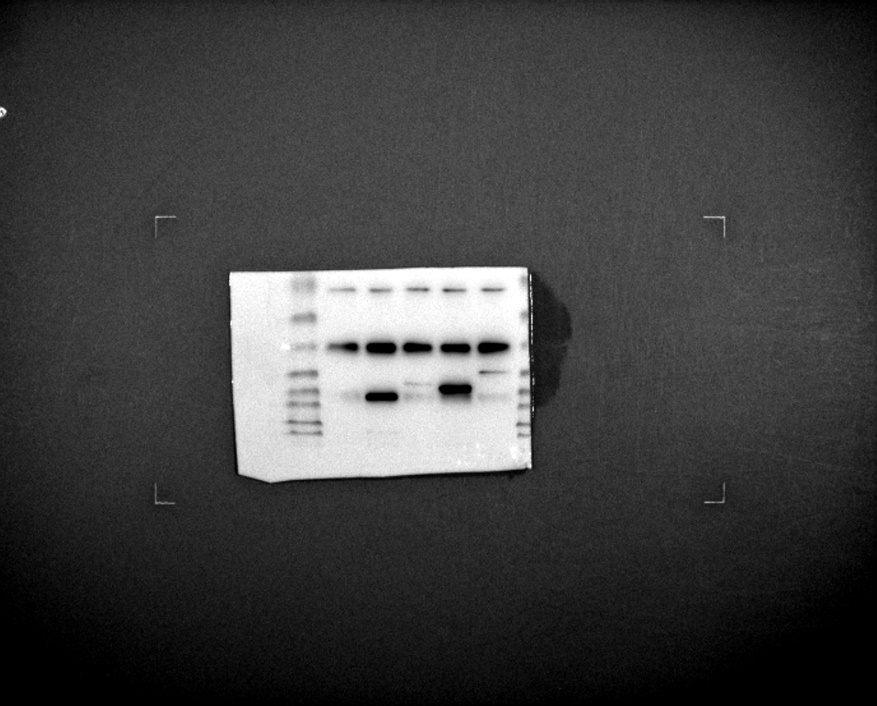

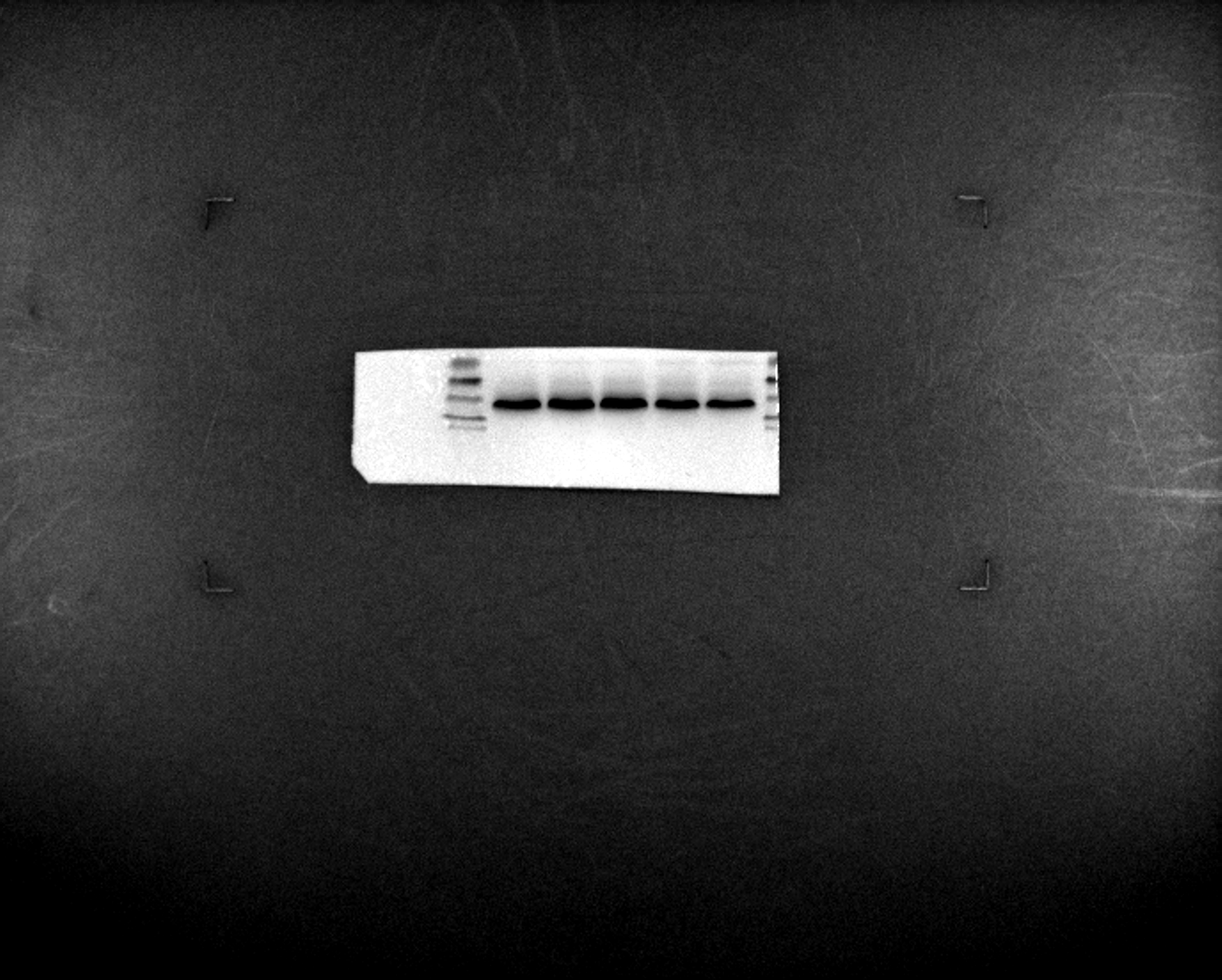

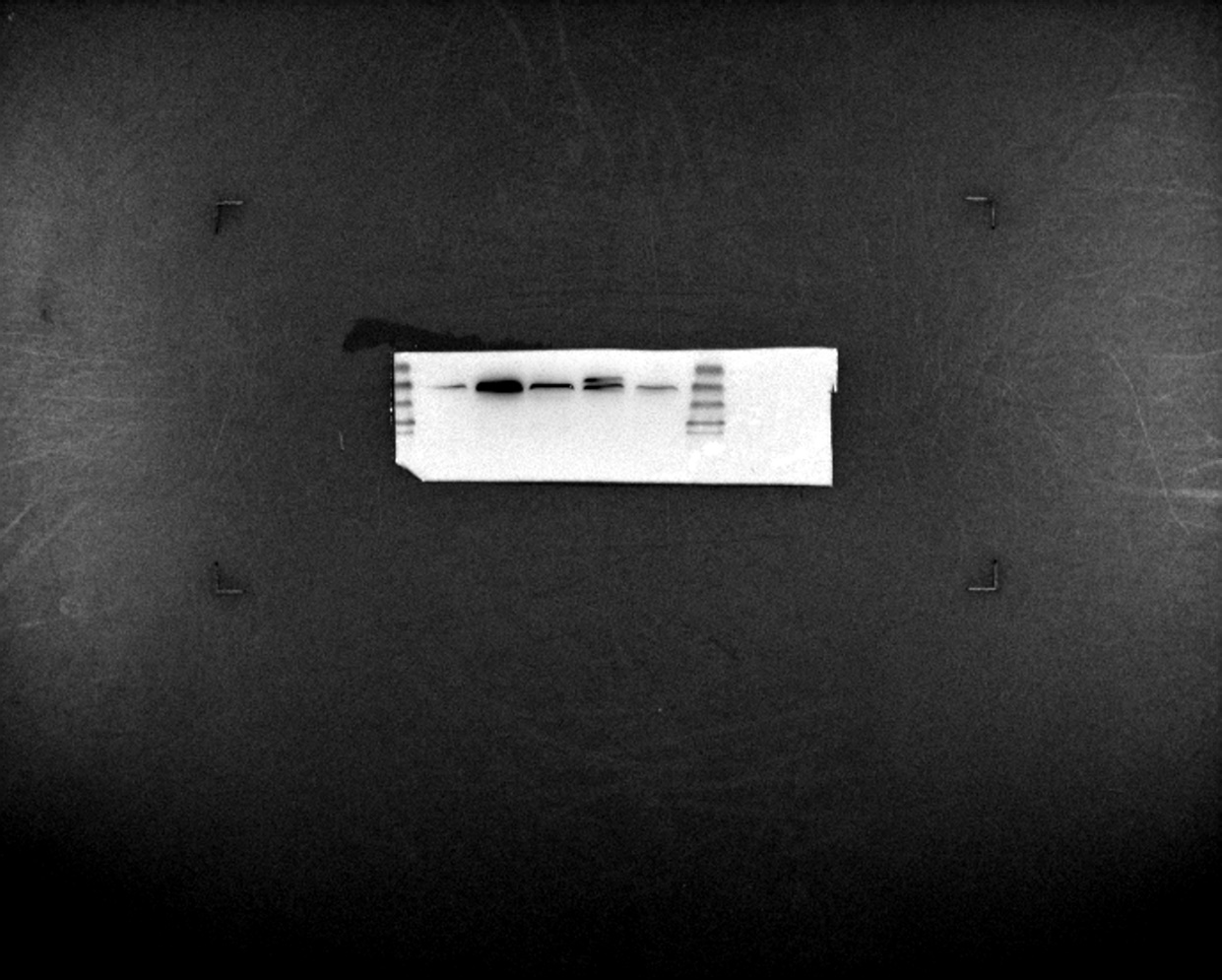

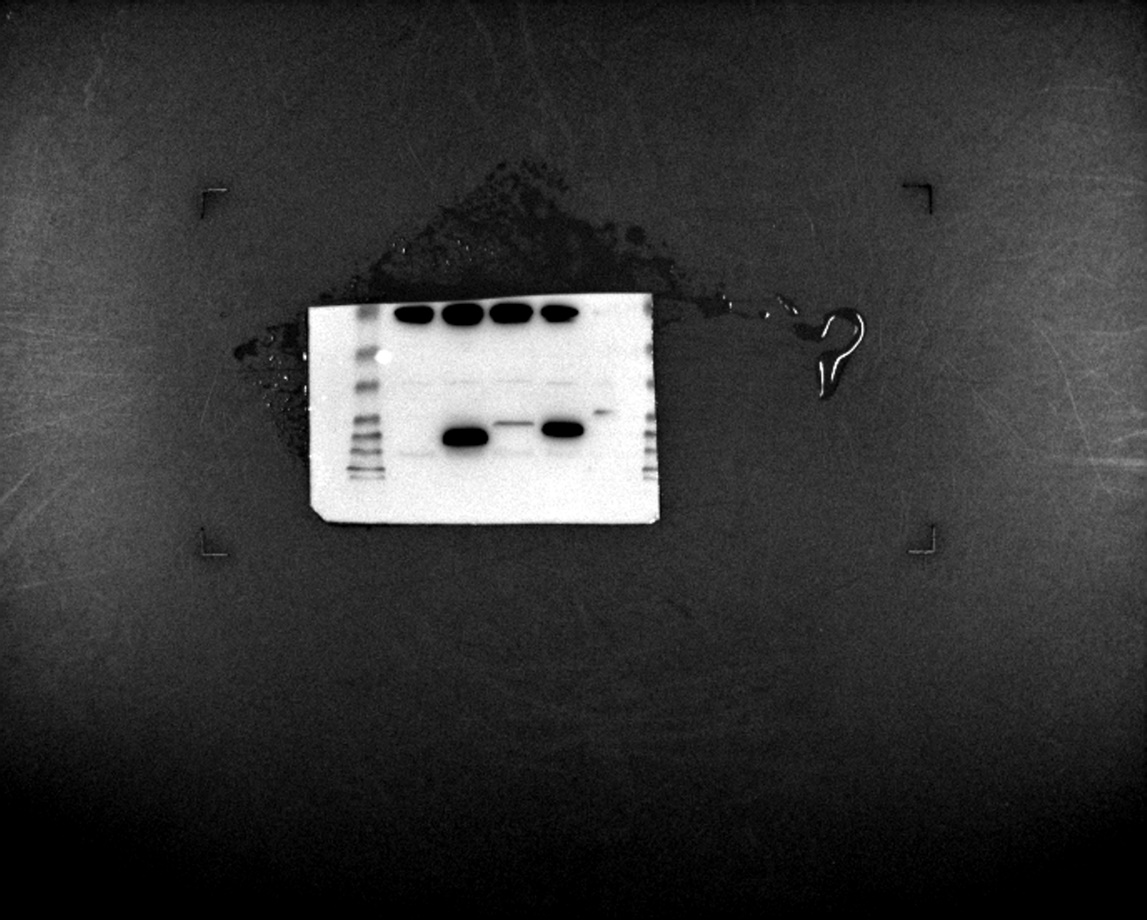

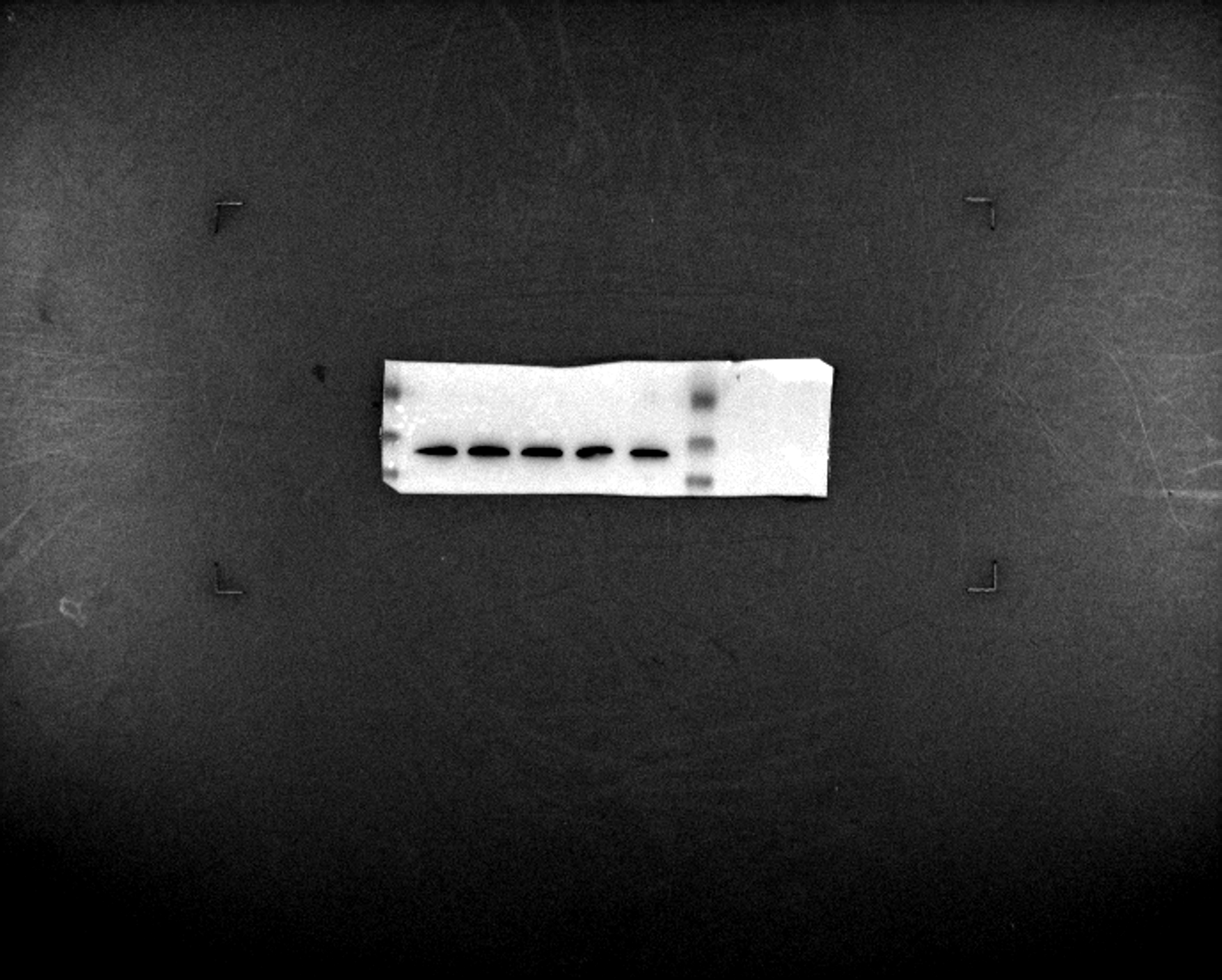


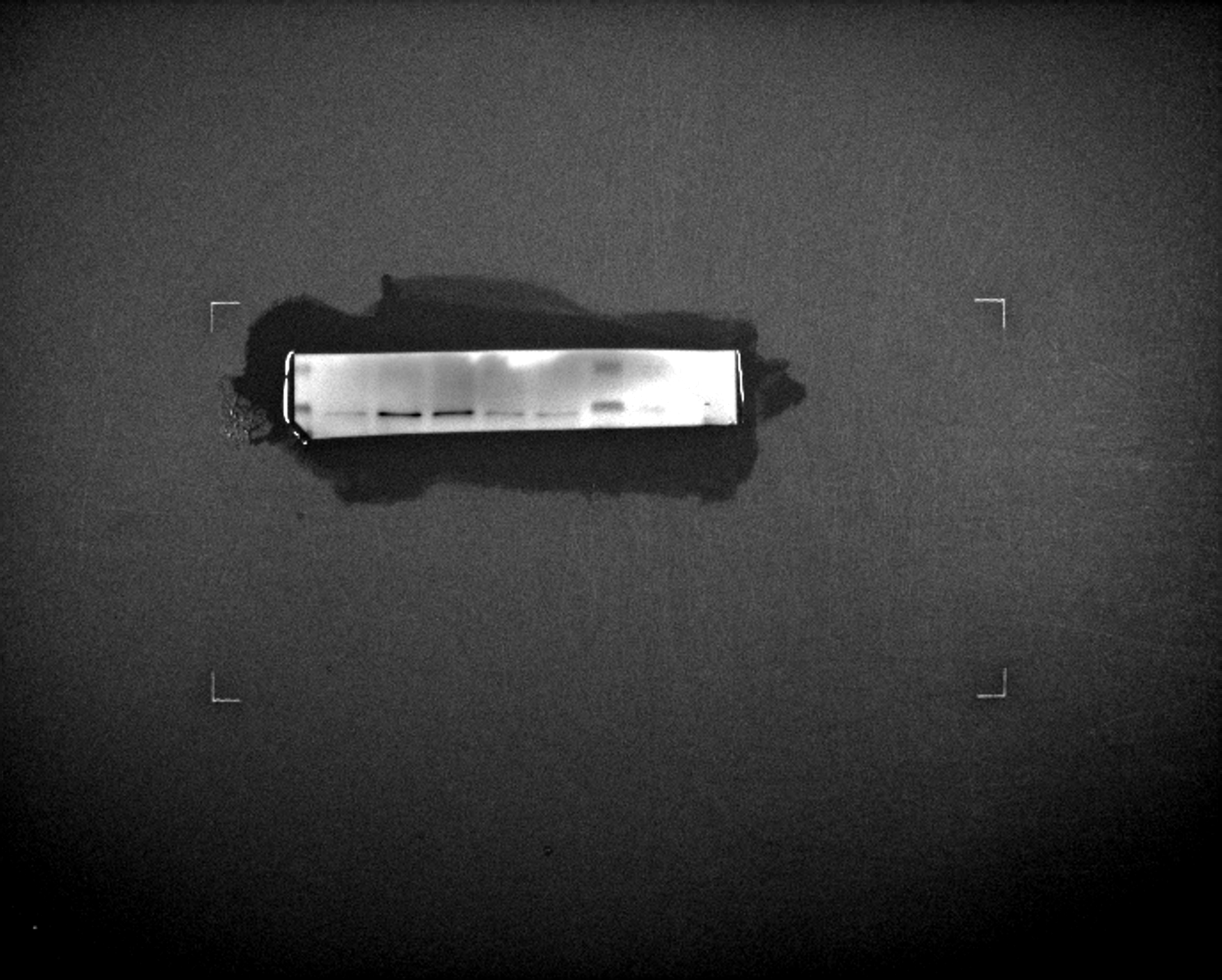

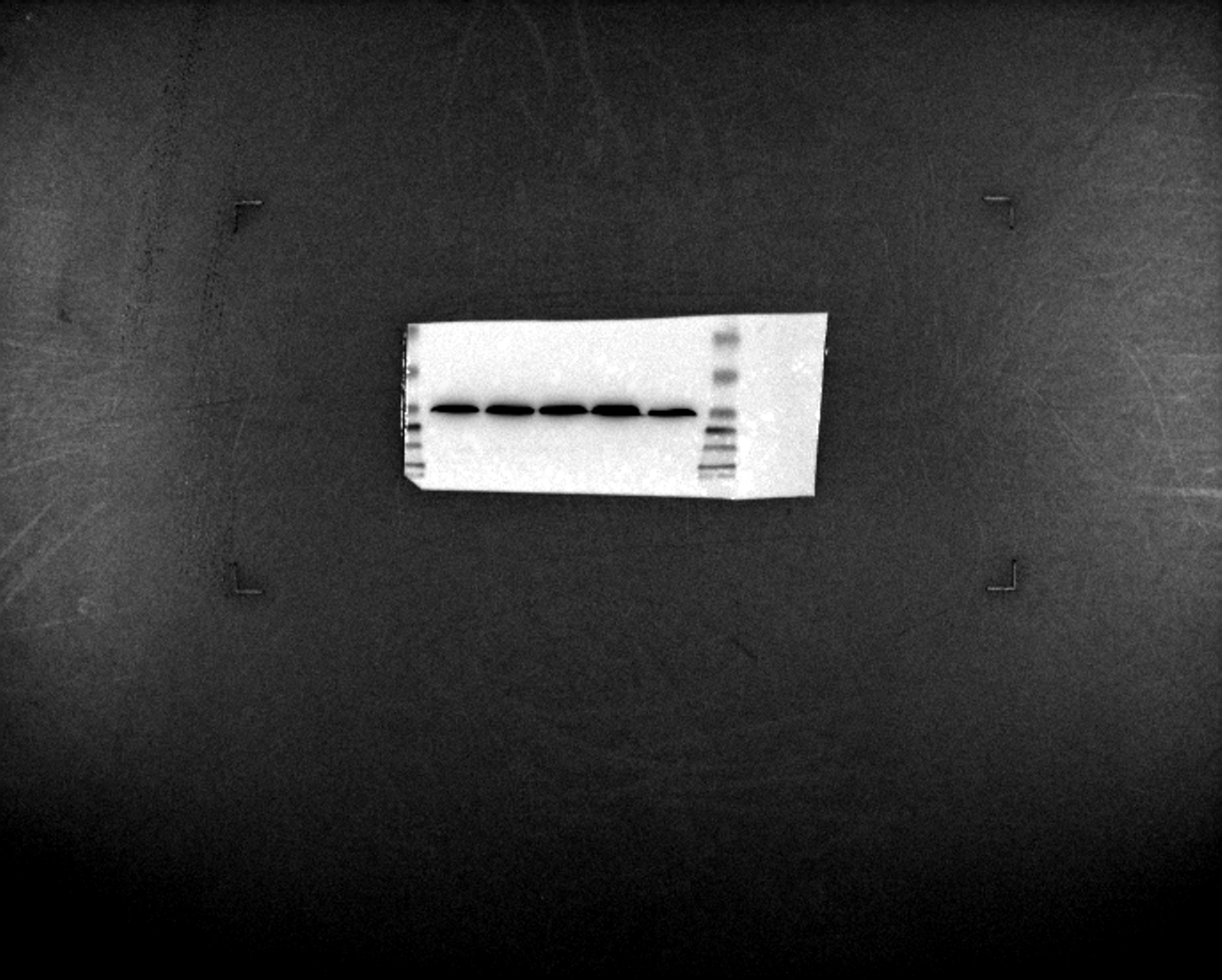


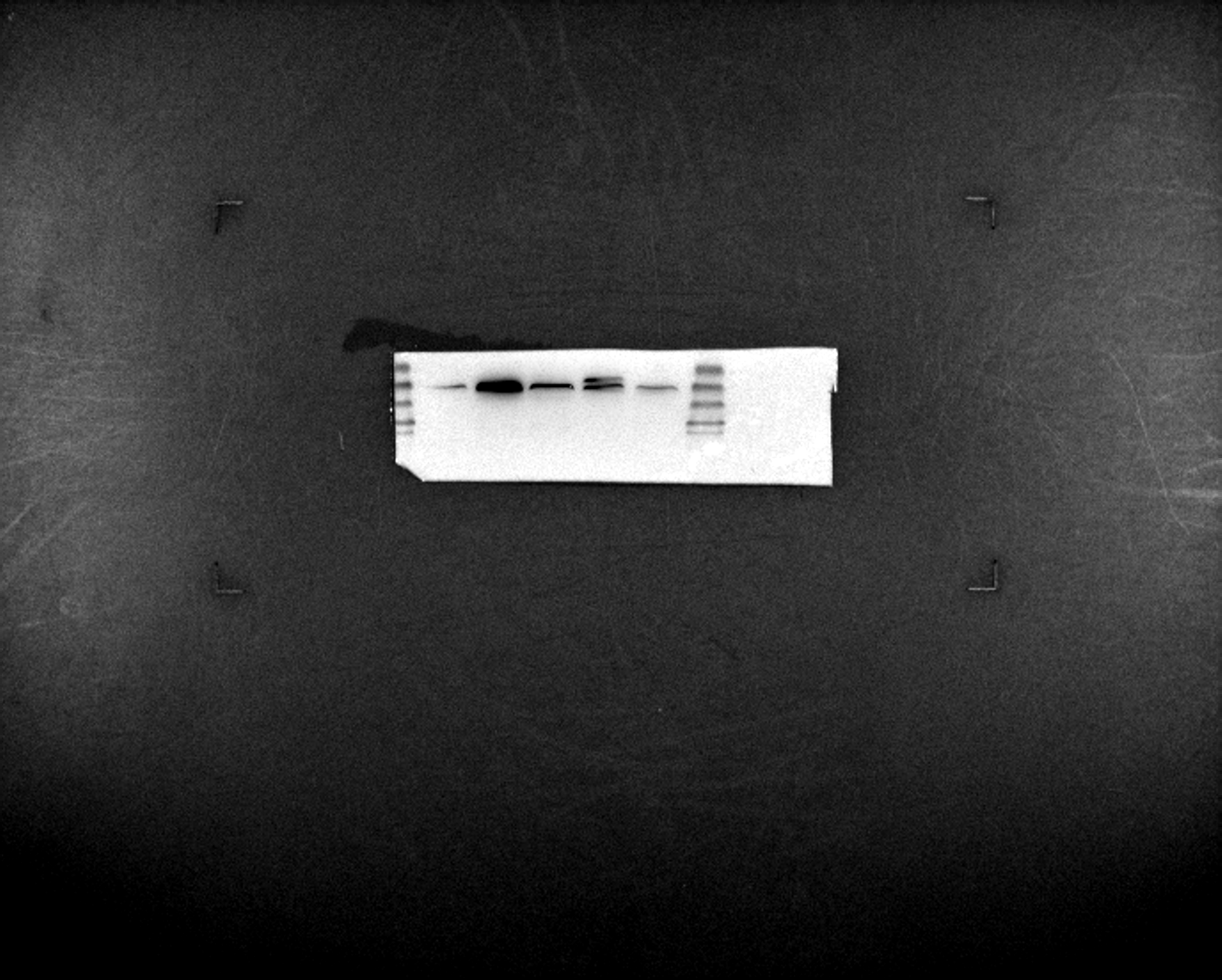


F:( RAD51AP1, E2F1, Actin)


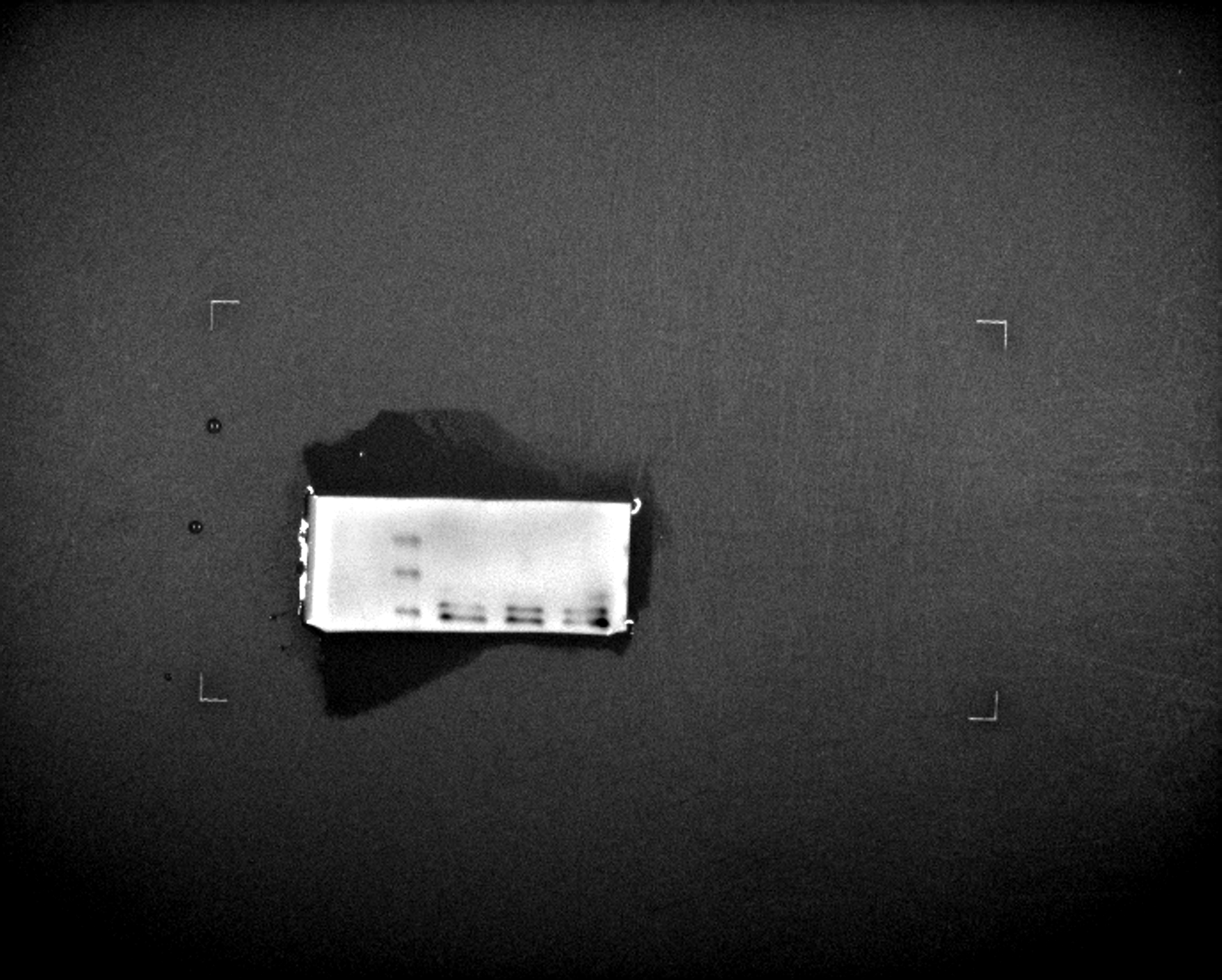

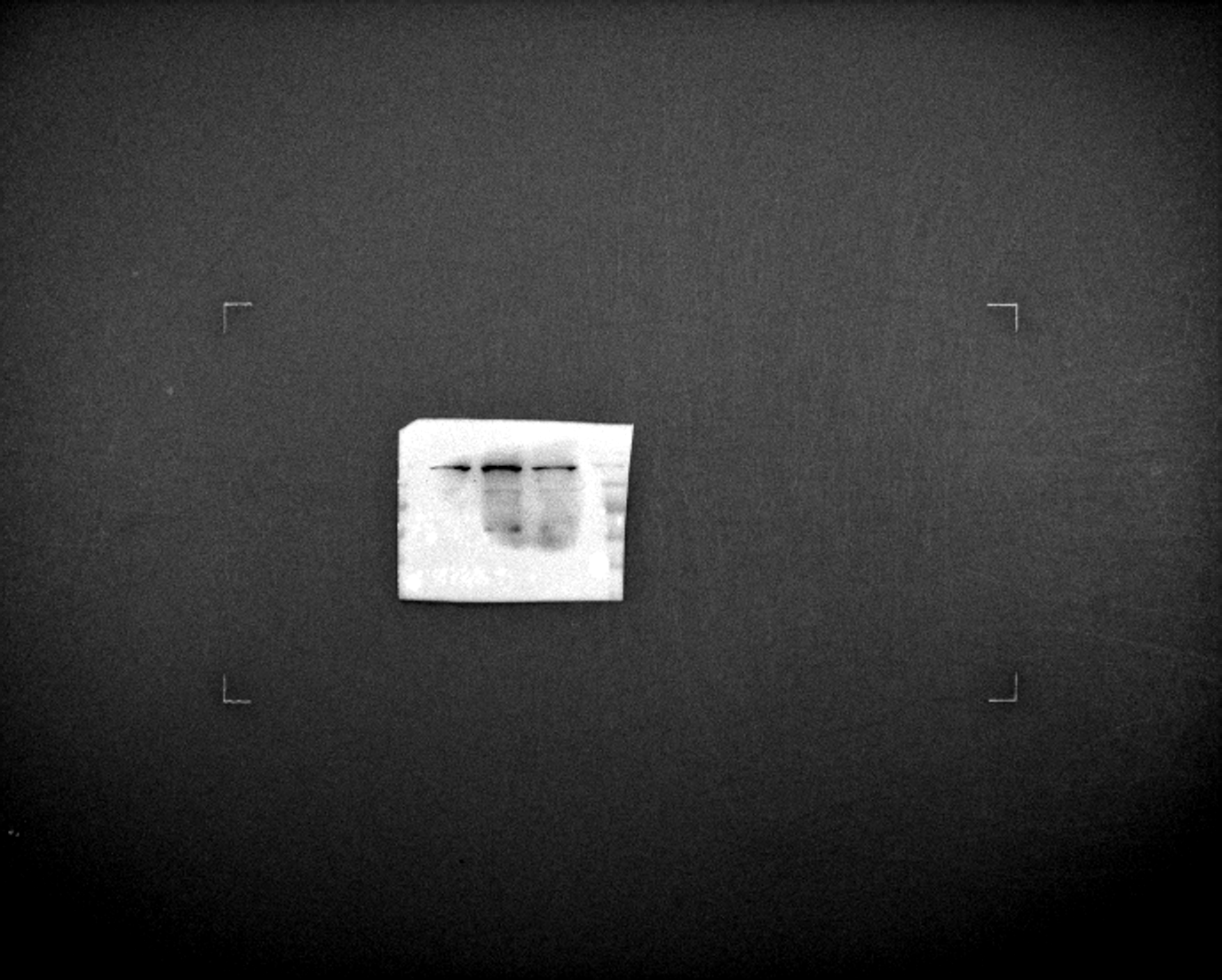

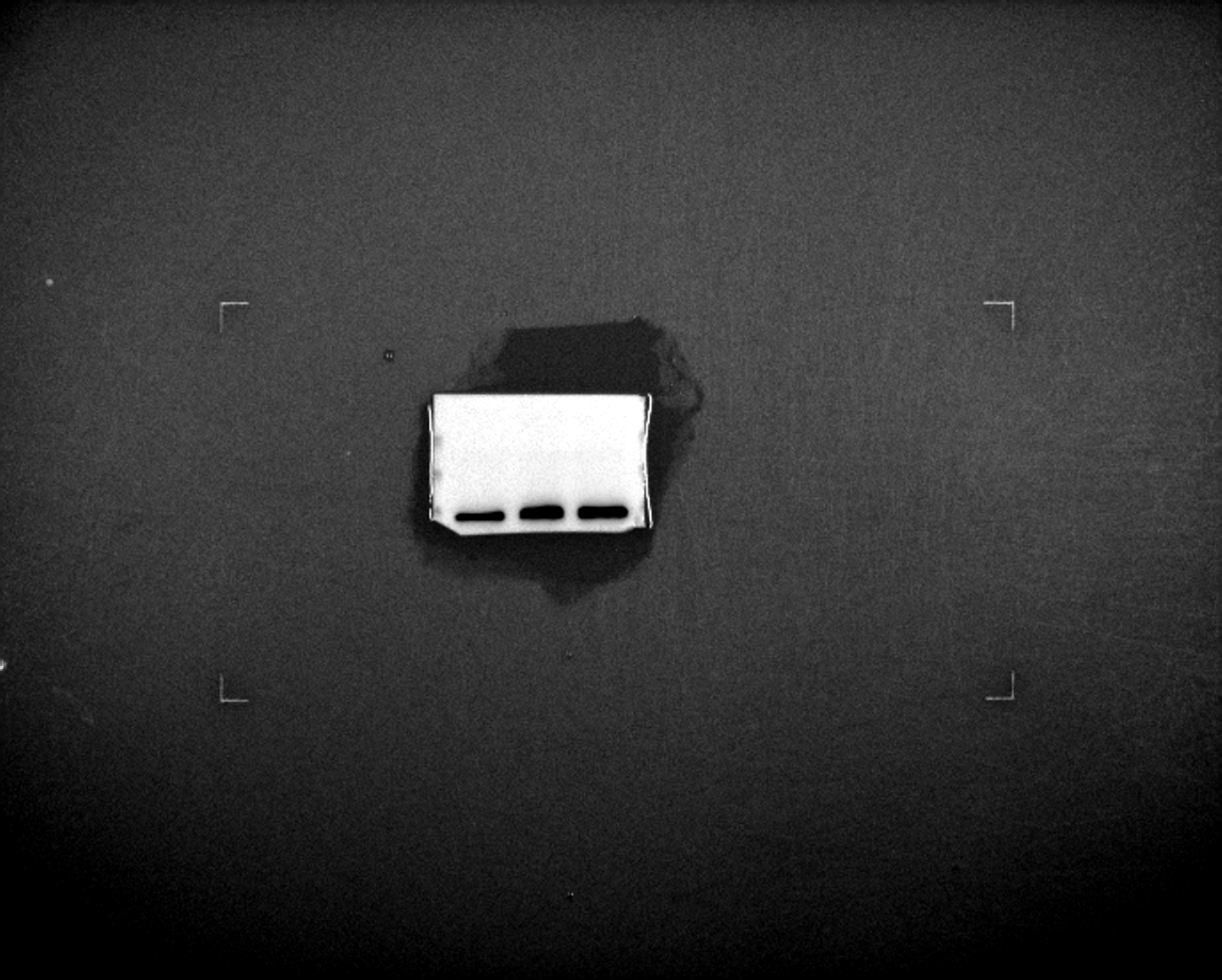


G:( RRM1, IP-USP11, IP-E2F1, Input-Flag, Actin)


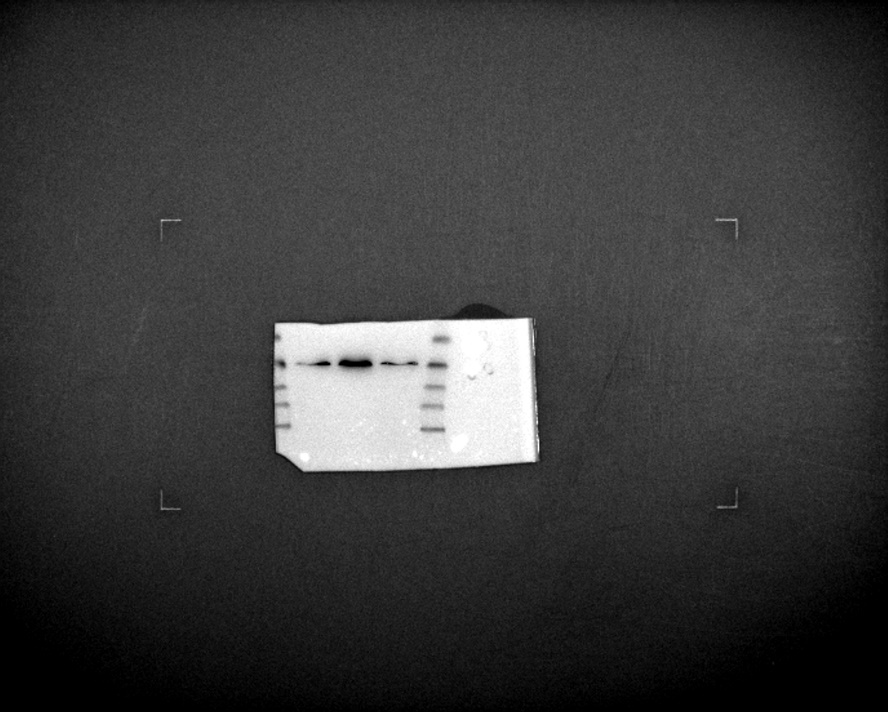

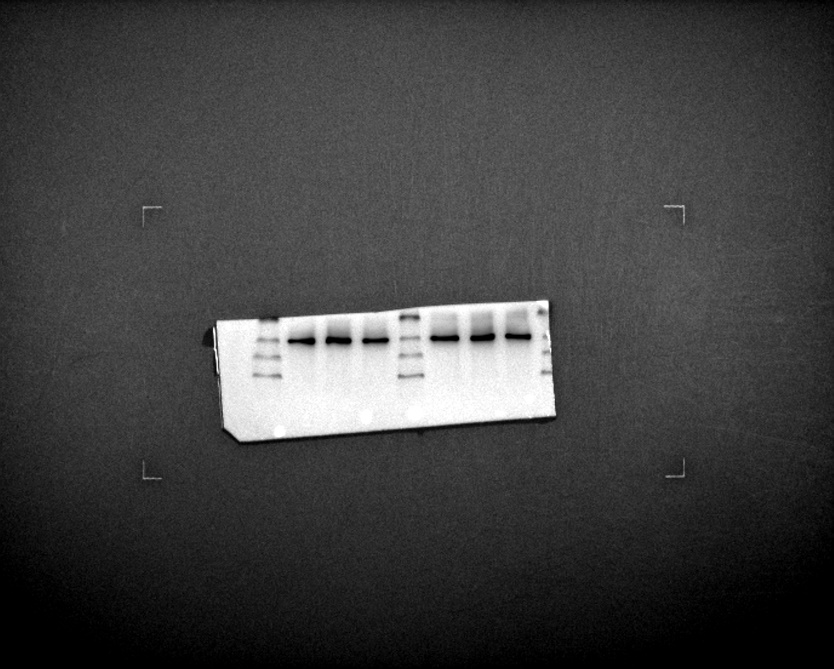

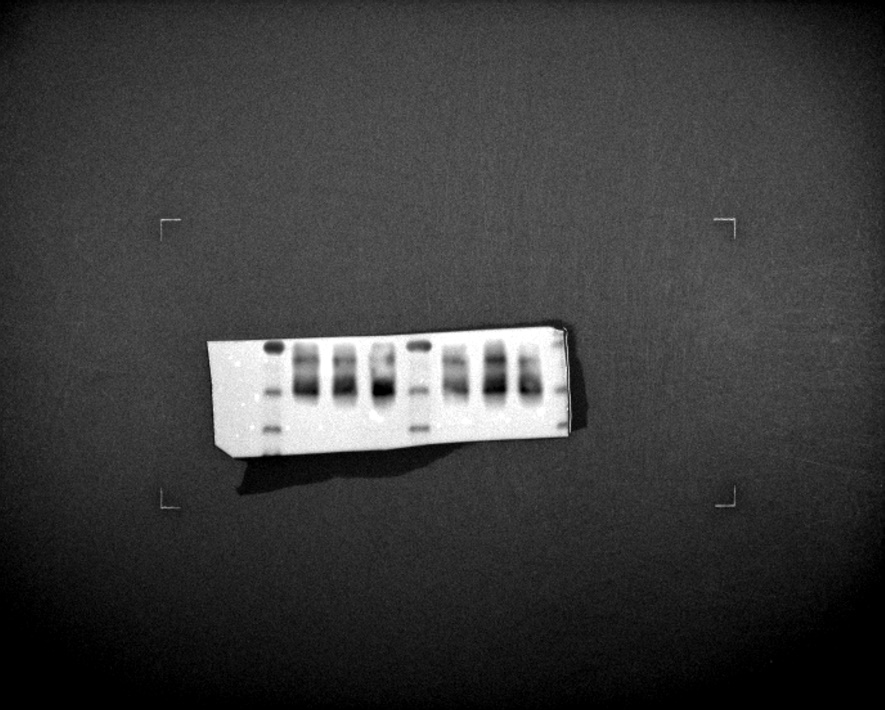

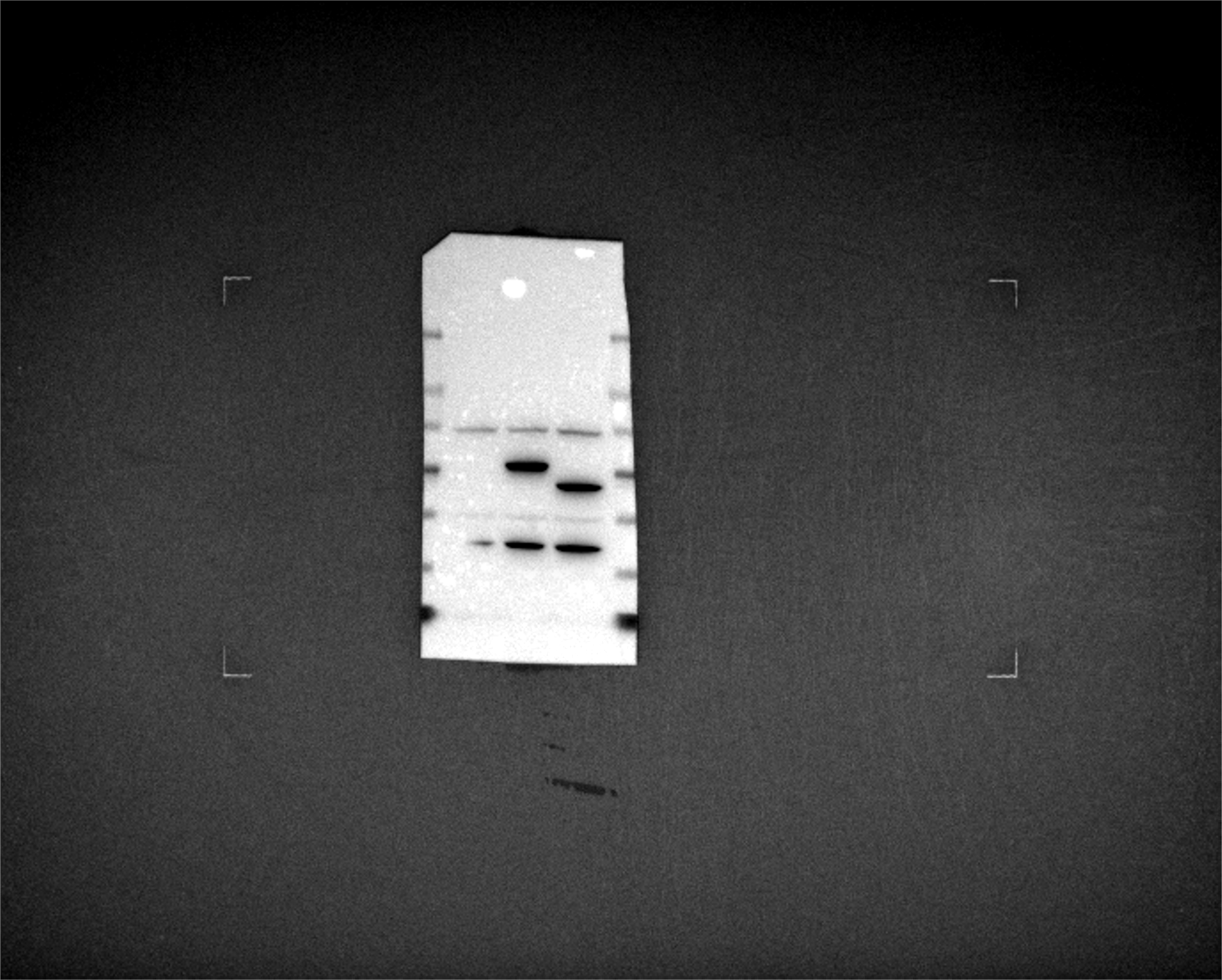

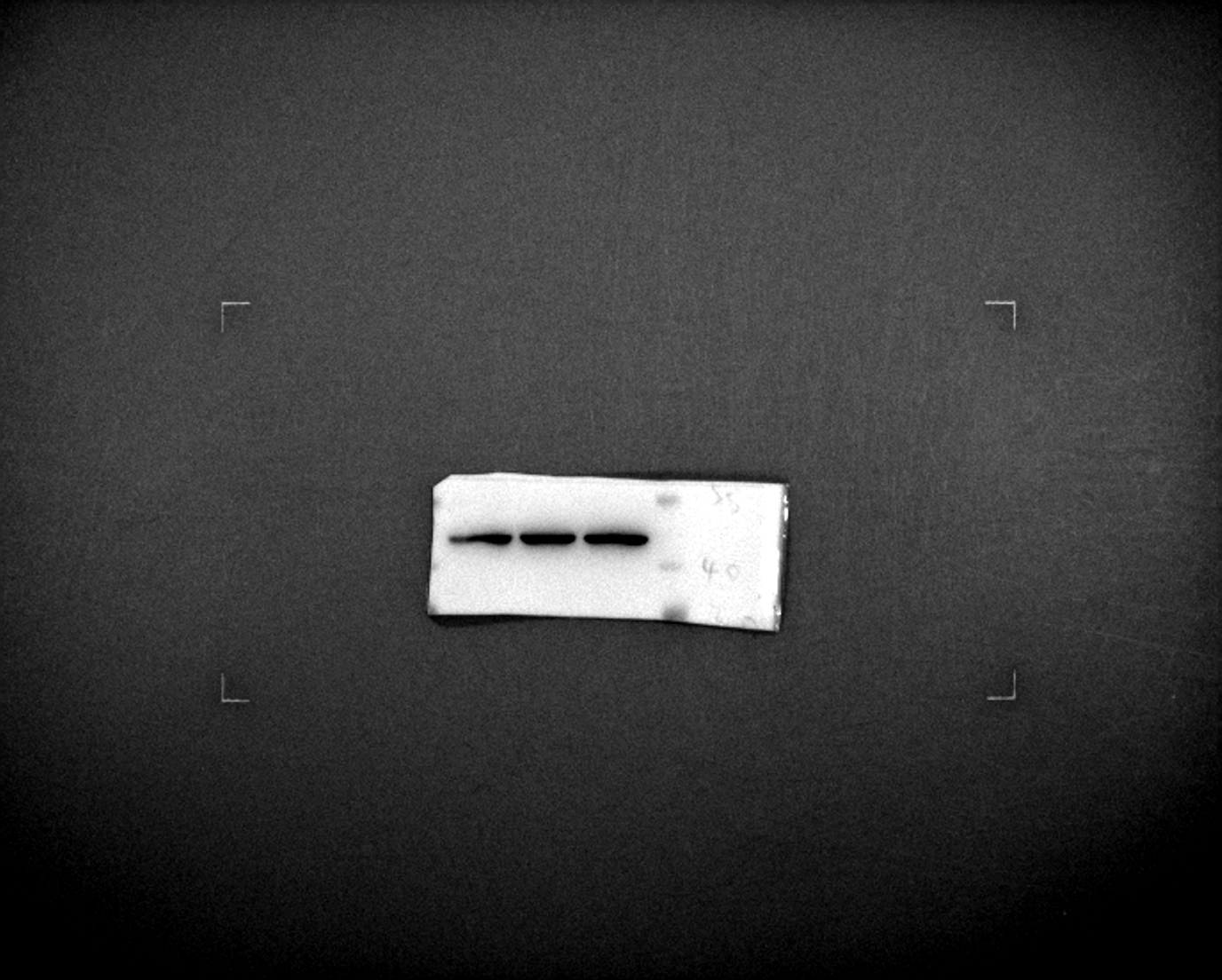


I:( Ub, IP-E2F1, Input-RRM1, Input-Flag, Input-Actin)

K:( E2F1, Actin)
